# The Influence of Age and Sex on Genetic Associations with Adult Body Size and Shape: A Large-Scale Genome-Wide Interaction Study

**DOI:** 10.1371/journal.pgen.1005378

**Published:** 2015-10-01

**Authors:** Thomas W. Winkler, Anne E. Justice, Mariaelisa Graff, Llilda Barata, Mary F. Feitosa, Su Chu, Jacek Czajkowski, Tõnu Esko, Tove Fall, Tuomas O. Kilpeläinen, Yingchang Lu, Reedik Mägi, Evelin Mihailov, Tune H. Pers, Sina Rüeger, Alexander Teumer, Georg B. Ehret, Teresa Ferreira, Nancy L. Heard-Costa, Juha Karjalainen, Vasiliki Lagou, Anubha Mahajan, Michael D. Neinast, Inga Prokopenko, Jeannette Simino, Tanya M. Teslovich, Rick Jansen, Harm-Jan Westra, Charles C. White, Devin Absher, Tarunveer S. Ahluwalia, Shafqat Ahmad, Eva Albrecht, Alexessander Couto Alves, Jennifer L. Bragg-Gresham, Anton J. M. de Craen, Joshua C. Bis, Amélie Bonnefond, Gabrielle Boucher, Gemma Cadby, Yu-Ching Cheng, Charleston W. K. Chiang, Graciela Delgado, Ayse Demirkan, Nicole Dueker, Niina Eklund, Gudny Eiriksdottir, Joel Eriksson, Bjarke Feenstra, Krista Fischer, Francesca Frau, Tessel E. Galesloot, Frank Geller, Anuj Goel, Mathias Gorski, Tanja B. Grammer, Stefan Gustafsson, Saskia Haitjema, Jouke-Jan Hottenga, Jennifer E. Huffman, Anne U. Jackson, Kevin B. Jacobs, Åsa Johansson, Marika Kaakinen, Marcus E. Kleber, Jari Lahti, Irene Mateo Leach, Benjamin Lehne, Youfang Liu, Ken Sin Lo, Mattias Lorentzon, Jian'an Luan, Pamela A. F. Madden, Massimo Mangino, Barbara McKnight, Carolina Medina-Gomez, Keri L. Monda, May E. Montasser, Gabriele Müller, Martina Müller-Nurasyid, Ilja M. Nolte, Kalliope Panoutsopoulou, Laura Pascoe, Lavinia Paternoster, Nigel W. Rayner, Frida Renström, Federica Rizzi, Lynda M. Rose, Kathy A. Ryan, Perttu Salo, Serena Sanna, Hubert Scharnagl, Jianxin Shi, Albert Vernon Smith, Lorraine Southam, Alena Stančáková, Valgerdur Steinthorsdottir, Rona J. Strawbridge, Yun Ju Sung, Ioanna Tachmazidou, Toshiko Tanaka, Gudmar Thorleifsson, Stella Trompet, Natalia Pervjakova, Jonathan P. Tyrer, Liesbeth Vandenput, Sander W van der Laan, Nathalie van der Velde, Jessica van Setten, Jana V. van Vliet-Ostaptchouk, Niek Verweij, Efthymia Vlachopoulou, Lindsay L. Waite, Sophie R. Wang, Zhaoming Wang, Sarah H. Wild, Christina Willenborg, James F. Wilson, Andrew Wong, Jian Yang, Loïc Yengo, Laura M. Yerges-Armstrong, Lei Yu, Weihua Zhang, Jing Hua Zhao, Ehm A. Andersson, Stephan J. L. Bakker, Damiano Baldassarre, Karina Banasik, Matteo Barcella, Cristina Barlassina, Claire Bellis, Paola Benaglio, John Blangero, Matthias Blüher, Fabrice Bonnet, Lori L. Bonnycastle, Heather A. Boyd, Marcel Bruinenberg, Aron S Buchman, Harry Campbell, Yii-Der Ida Chen, Peter S. Chines, Simone Claudi-Boehm, John Cole, Francis S. Collins, Eco J. C. de Geus, Lisette C. P. G. M. de Groot, Maria Dimitriou, Jubao Duan, Stefan Enroth, Elodie Eury, Aliki-Eleni Farmaki, Nita G. Forouhi, Nele Friedrich, Pablo V. Gejman, Bruna Gigante, Nicola Glorioso, Alan S. Go, Omri Gottesman, Jürgen Gräßler, Harald Grallert, Niels Grarup, Yu-Mei Gu, Linda Broer, Annelies C. Ham, Torben Hansen, Tamara B. Harris, Catharina A. Hartman, Maija Hassinen, Nicholas Hastie, Andrew T. Hattersley, Andrew C. Heath, Anjali K. Henders, Dena Hernandez, Hans Hillege, Oddgeir Holmen, Kees G Hovingh, Jennie Hui, Lise L. Husemoen, Nina Hutri-Kähönen, Pirro G. Hysi, Thomas Illig, Philip L. De Jager, Shapour Jalilzadeh, Torben Jørgensen, J. Wouter Jukema, Markus Juonala, Stavroula Kanoni, Maria Karaleftheri, Kay Tee Khaw, Leena Kinnunen, Steven J. Kittner, Wolfgang Koenig, Ivana Kolcic, Peter Kovacs, Nikolaj T. Krarup, Wolfgang Kratzer, Janine Krüger, Diana Kuh, Meena Kumari, Theodosios Kyriakou, Claudia Langenberg, Lars Lannfelt, Chiara Lanzani, Vaneet Lotay, Lenore J. Launer, Karin Leander, Jaana Lindström, Allan Linneberg, Yan-Ping Liu, Stéphane Lobbens, Robert Luben, Valeriya Lyssenko, Satu Männistö, Patrik K. Magnusson, Wendy L. McArdle, Cristina Menni, Sigrun Merger, Lili Milani, Grant W. Montgomery, Andrew P. Morris, Narisu Narisu, Mari Nelis, Ken K. Ong, Aarno Palotie, Louis Pérusse, Irene Pichler, Maria G. Pilia, Anneli Pouta, Myriam Rheinberger, Rasmus Ribel-Madsen, Marcus Richards, Kenneth M. Rice, Treva K. Rice, Carlo Rivolta, Veikko Salomaa, Alan R. Sanders, Mark A. Sarzynski, Salome Scholtens, Robert A. Scott, William R. Scott, Sylvain Sebert, Sebanti Sengupta, Bengt Sennblad, Thomas Seufferlein, Angela Silveira, P. Eline Slagboom, Jan H. Smit, Thomas H. Sparsø, Kathleen Stirrups, Ronald P. Stolk, Heather M. Stringham, Morris A Swertz, Amy J. Swift, Ann-Christine Syvänen, Sian-Tsung Tan, Barbara Thorand, Anke Tönjes, Angelo Tremblay, Emmanouil Tsafantakis, Peter J. van der Most, Uwe Völker, Marie-Claude Vohl, Judith M. Vonk, Melanie Waldenberger, Ryan W. Walker, Roman Wennauer, Elisabeth Widén, Gonneke Willemsen, Tom Wilsgaard, Alan F. Wright, M. Carola Zillikens, Suzanne C. van Dijk, Natasja M. van Schoor, Folkert W. Asselbergs, Paul I. W. de Bakker, Jacques S. Beckmann, John Beilby, David A. Bennett, Richard N. Bergman, Sven Bergmann, Carsten A. Böger, Bernhard O. Boehm, Eric Boerwinkle, Dorret I. Boomsma, Stefan R. Bornstein, Erwin P. Bottinger, Claude Bouchard, John C. Chambers, Stephen J. Chanock, Daniel I. Chasman, Francesco Cucca, Daniele Cusi, George Dedoussis, Jeanette Erdmann, Johan G. Eriksson, Denis A. Evans, Ulf de Faire, Martin Farrall, Luigi Ferrucci, Ian Ford, Lude Franke, Paul W. Franks, Philippe Froguel, Ron T. Gansevoort, Christian Gieger, Henrik Grönberg, Vilmundur Gudnason, Ulf Gyllensten, Per Hall, Anders Hamsten, Pim van der Harst, Caroline Hayward, Markku Heliövaara, Christian Hengstenberg, Andrew A Hicks, Aroon Hingorani, Albert Hofman, Frank Hu, Heikki V. Huikuri, Kristian Hveem, Alan L. James, Joanne M. Jordan, Antti Jula, Mika Kähönen, Eero Kajantie, Sekar Kathiresan, Lambertus A. L. M. Kiemeney, Mika Kivimaki, Paul B. Knekt, Heikki A. Koistinen, Jaspal S. Kooner, Seppo Koskinen, Johanna Kuusisto, Winfried Maerz, Nicholas G Martin, Markku Laakso, Timo A. Lakka, Terho Lehtimäki, Guillaume Lettre, Douglas F. Levinson, Lars Lind, Marja-Liisa Lokki, Pekka Mäntyselkä, Mads Melbye, Andres Metspalu, Braxton D. Mitchell, Frans L. Moll, Jeffrey C. Murray, Arthur W. Musk, Markku S. Nieminen, Inger Njølstad, Claes Ohlsson, Albertine J. Oldehinkel, Ben A. Oostra, Lyle J Palmer, James S. Pankow, Gerard Pasterkamp, Nancy L. Pedersen, Oluf Pedersen, Brenda W. Penninx, Markus Perola, Annette Peters, Ozren Polašek, Peter P. Pramstaller, Bruce M. Psaty, Lu Qi, Thomas Quertermous, Olli T. Raitakari, Tuomo Rankinen, Rainer Rauramaa, Paul M. Ridker, John D. Rioux, Fernando Rivadeneira, Jerome I. Rotter, Igor Rudan, Hester M. den Ruijter, Juha Saltevo, Naveed Sattar, Heribert Schunkert, Peter E. H. Schwarz, Alan R. Shuldiner, Juha Sinisalo, Harold Snieder, Thorkild I. A. Sørensen, Tim D. Spector, Jan A. Staessen, Bandinelli Stefania, Unnur Thorsteinsdottir, Michael Stumvoll, Jean-Claude Tardif, Elena Tremoli, Jaakko Tuomilehto, André G. Uitterlinden, Matti Uusitupa, André L. M. Verbeek, Sita H. Vermeulen, Jorma S. Viikari, Veronique Vitart, Henry Völzke, Peter Vollenweider, Gérard Waeber, Mark Walker, Henri Wallaschofski, Nicholas J. Wareham, Hugh Watkins, Eleftheria Zeggini, CHARGE Consortium, DIAGRAM Consortium, GLGC Consortium, Global-BPGen Consortium, ICBP Consortium, MAGIC Consortium, Aravinda Chakravarti, Deborah J. Clegg, L. Adrienne Cupples, Penny Gordon-Larsen, Cashell E. Jaquish, D. C. Rao, Goncalo R. Abecasis, Themistocles L. Assimes, Inês Barroso, Sonja I. Berndt, Michael Boehnke, Panos Deloukas, Caroline S. Fox, Leif C. Groop, David J. Hunter, Erik Ingelsson, Robert C. Kaplan, Mark I. McCarthy, Karen L. Mohlke, Jeffrey R. O'Connell, David Schlessinger, David P. Strachan, Kari Stefansson, Cornelia M. van Duijn, Joel N. Hirschhorn, Cecilia M. Lindgren, Iris M. Heid, Kari E. North, Ingrid B. Borecki, Zoltán Kutalik, Ruth J. F. Loos

**Affiliations:** 1 Department of Genetic Epidemiology, Institute of Epidemiology and Preventive Medicine, University Regensburg, Regensburg, Germany; 2 Department of Epidemiology, University of North Carolina at Chapel Hill, Chapel Hill, North Carolina, United States of America; 3 Division of Statistical Genomics, Department of Genetics, Washington University School of Medicine, St. Louis, Missouri, United States of America; 4 Center for Statistical Genetics, Department of Biostatistics, University of Michigan, Ann Arbor, Michigan, United States of America; 5 Broad Institute of the Massachusetts Institute of Technology and Harvard University, Cambridge, Massachusetts, United States of America; 6 Divisions of Endocrinology and Genetics and Center for Basic and Translational Obesity Research, Boston Children's Hospital, Boston, Massachusetts, United States of America; 7 Estonian Genome Center, Univeristy of Tartu, Tartu, Estonia; 8 Department of Genetics, Harvard Medical School, Boston, Massachusetts, United States of America; 9 Science for Life Laboratory, Uppsala University, Uppsala, Sweden; 10 Department of Medical Sciences, Molecular Epidemiology, Uppsala University, Uppsala, Sweden; 11 Novo Nordisk Foundation Center for Basic Metabolic Research, Section of Metabolic Genetics, Faculty of Health and Medical Sciences, University of Copenhagen, Copenhagen, Denmark; 12 MRC Epidemiology Unit, Institute of Metabolic Science, University of Cambridge, Cambridge, United Kingdom; 13 The Charles Bronfman Institute for Personalized Medicine, The Icahn School of Medicine at Mount Sinai, New York, New York, United States of America; 14 The Department of Preventive Medicine, The Icahn School of Medicine at Mount Sinai, New York, New York, United States of America; 15 Wellcome Trust Centre for Human Genetics, University of Oxford, Oxford, United Kingdom; 16 Medical and Population Genetics Program, Broad Institute of MIT and Harvard, Cambridge, Massachusetts, United States of America; 17 Swiss Institute of Bioinformatics, Lausanne, Switzerland; 18 Institute of Social and Preventive Medicine, University Hospital Lausanne (CHUV), Lausanne, Switzerland; 19 Institute for Community Medicine, University Medicine Greifswald, Greifswald, Germany; 20 Interfaculty Institute for Genetics and Functional Genomics, University Medicine Greifswald, Greifswald, Germany; 21 Department of Specialties of Internal Medicine, Geneva University Hospital, Geneva, Switzerland; 22 Center for Complex Disease Genomics, McKusick-Nathans Institute of Genetic Medicine, Johns Hopkins University School of Medicine, Baltimore, Maryland, United States of America; 23 Department of Neurology, Boston University School of Medicine, Boston, Massachusetts, United States of America; 24 National Heart, Lung, and Blood Institute, the Framingham Heart Study, Framingham, Massachusetts, United States of America; 25 University of Groningen, University Medical Center Groningen, Department of Genetics, Groningen, The Netherlands; 26 Oxford Centre for Diabetes, Endocrinology and Metabolism, University of Oxford, Churchill Hospital, Oxford, United Kingdom; 27 Department of Internal Medicine, University of Texas Southwestern Medical Center, Dallas, Texas, United States of America; 28 Hammersmith Hospital, London, United Kingdom; 29 Department of Genomics of Common Diseases, School of Public Health, Imperial College London, London, United Kingdom; 30 Division of Biostatistics, Washington University School of Medicine, St. Louis, Missouri, United States of America; 31 Department of Psychiatry and EMGO Institute for Health and Care Research, VU University Medical Center, Amsterdam, the Netherlands; 32 Program in Medical and Population Genetics, Broad Institute of Harvard and Massachusetts Institute of Technology, Cambridge, Massachusetts, United States of America; 33 Divisions of Genetics and Rheumatology, Department of Medicine, Brigham and Women’s Hospital and Harvard Medical School, Boston, Massachusetts, United States of America; 34 Partners Center for Personalized Genetic Medicine, Boston, Massachusetts, United States of America; 35 Department of Biostatistics, Boston University School of Public Health, Boston, Massachusetts, United States of America; 36 HudsonAlpha Institute for Biotechnology, Huntsville, Alabama, United States of America; 37 Steno Diabetes Center A/S, Gentofte, Denmark; 38 COPSAC, Copenhagen Prospective Studies on Asthma in Childhood, Herlev and Gentofte Hospital, University of Copenhagen, Copenhagen, Denmark; 39 Department of Clinical Sciences, Genetic and Molecular Epidemiology Unit, Skåne University Hospital Malmö, Malmö, Sweden; 40 Institute of Genetic Epidemiology, Helmholtz Zentrum München—German Research Center for Environmental Health, Neuherberg, Germany; 41 Department of Epidemiology and Biostatistics, MRC Health Protection Agency (HPA) Centre for Environment and Health, School of Public Health, Imperial College, London, United Kingdom; 42 Department of Gerontology and Geriatrics, Leiden University Medical Center, Leiden, The Netherlands; 43 Cardiovascular Health Research Unit, University of Washington, Seattle, Washington, United States of America; 44 Department of Medicine, University of Washington, Seattle, Washington, United States of America; 45 CNRS UMR 8199, Lille, France; 46 European Genomic Institute for Diabetes, Lille, France; 47 Université de Lille 2, Lille, France; 48 Montreal Heart Institute, Montréal, Québec, Canada; 49 Centre for Genetic Origins of Health and Disease, University of Western Australia, Crawley, Western Australia, Australia; 50 VA Maryland Health Care System, Baltimore, Maryland, United States of America; 51 University of Maryland School of Medicine, Department of Medicine, Baltimore, Maryland, United States of America; 52 Department of Ecology and Evolutionary Biology, University of California, Los Angeles, Los Angeles, California, United States of America; 53 Vth Department of Medicine, Mannheim Medical Faculty, University of Heidelberg, Mannheim, Germany; 54 Genetic Epidemiology Unit, Department of Epidemiology, Erasmus University Medical Center, Rotterdam, The Netherlands; 55 Universiy of Maryland School of Medicine, Department of Epidemiology & Public Health, Baltimore, Maryland, United States of America; 56 National Institute for Health and Welfare, Department of Chronic Disease Prevention, Helsinki, Finland; 57 National Institute for Health and Welfare, Public Health Genomics Unit, Helsinki, Finland; 58 Institute for Molecular Medicine Finland, University of Helsinki, Helsinki, Finland; 59 Icelandic Heart Association, Kopavogur, Iceland; 60 Centre for Bone and Arthritis Research, Department of Internal Medicine and Clinical Nutrition, Institute of Medicine, Sahlgrenska Academy, University of Gothenburg, Gothenburg, Sweden; 61 Department of Epidemiology Research, Statens Serum Institut, Copenhagen, Denmark; 62 Department of Health Sciences, University of Milan, Milan, Italy; 63 Filarete Foundation, Genomic and Bioinformatics Unit, Milano, Italy; 64 Radboud university medical center, Radboud Institute for Health Sciences, Department for Health Evidence, Nijmegen, The Netherlands; 65 Division of Cardiovacular Medicine, Radcliffe Department of Medicine, University of Oxford, Oxford, United Kingdom; 66 Department of Nephrology, University Hospital Regensburg, Regensburg, Germany; 67 Experimental Cardiology and laboratory of clinical chemistry, UMCU, Utrecht, The Netherlands; 68 Department of Biological Psychology, VU University, Amsterdam, The Netherlands; 69 MRC Human Genetics Unit, Institute of Genetics and Molecular Medicine, University of Edinburgh, Edinburgh, Scotland; 70 Division of Cancer Epidemiology and Genetics, National Cancer Institute, National Institutes of Health, Bethesda, Maryland, United States of America; 71 Core Genotyping Facility, SAIC-Frederick, Inc., NCI-Frederick, Frederick, Maryland, United States of America; 72 Department of Immunology, Genetics and Pathology, Uppsala University, Uppsala, Sweden; 73 Institute of Health Sciences, University of Oulu, Oulu, Finland; 74 Folkhälsan Research Centre, Helsinki, Finland; 75 Institute of Behavioural Sciences, University of Helsinki, Helsinki, Finland; 76 University of Groningen, University Medical Center Groningen, Department of Cardiology, Groningen, Netherlands; 77 Department of Epidemiology and Biostatistics, Imperial College London, London, United Kingdom; 78 Thurston Arthritis Research Center, University of North Carolina at Chapel Hill, Chaper Hill, North Carolina, United States of America; 79 Washington University Medical School, St. Louis, Missouri, United States of America; 80 Department of Twin Research and Genetic Epidemiology, King's College London, London, United Kingdom; 81 Program in Biostatistics and Biomathematics, Divison of Public Health Sciences, Fred Hutchinson Cancer Research Center, Seattle, Washington, United States of America; 82 Department of Biostatistics, University of Washington, Seattle, Washington, United States of America; 83 Netherlands Genomics Initiative (NGI)-sponsored Netherlands Consortium for Healthy Aging (NCHA), The Netherlands; 84 Department of Epidemiology, Erasmus Medical Center, Rotterdam, The Netherlands; 85 Department of Internal Medicine, Erasmus Medical Center, Rotterdam, The Netherlands; 86 The Center for Observational Research, Amgen Inc., Thousand Oaks, California, United States of America; 87 Program for Personalized and Genomic Medicine, Division of Endocrinology, Diabetes & Nutrition, Dept of Medicine, University of Maryland School of Medicine, Baltimore, Maryland, United States of America; 88 Center for Evidence Based Healthcare, University of Dresden, Medical Faculty Carl Gustav Carus, Dresden, Germany; 89 Department of Medicine I, University Hospital Grosshadern, Ludwig-Maximilians-Universität, Munich, Germany; 90 Institute of Medical Informatics, Biometry and Epidemiology, Chair of Genetic Epidemiology, Ludwig-Maximilians-Universität, Munich, Germany; 91 DZHK (German Centre for Cardiovascular Research), partnersite Munich Heart Alliance, Munich, Germany; 92 University of Groningen, University Medical Center Groningen, Department of Epidemiology, Groningen, The Netherlands; 93 Wellcome Trust Sanger Institute, Human Genetics, Hinxton, Cambridge, United Kingdom; 94 Institute of Cell & Molecular Biosciences, Newcastle University, Newcastle, United Kingdom; 95 MRC Integrative Epidemiology Unit, School of Social and Community Medicine, University of Bristol, Bristol, United Kingdom; 96 Division of Preventive Medicine, Brigham and Women's Hospital, Boston, Massachusetts, United States of America; 97 Istituto di Ricerca Genetica e Biomedica, CNR, Monserrato, Italy; 98 Clinical Institute of Medical and Chemical Laboratory Diagnostics, Medical University of Graz, Graz, Austria; 99 National Cancer Institute, Bethesda, Maryland, United States of America; 100 Faculty of Medicine, University of Iceland, Reykjavik, Iceland; 101 Department of Medicine, University of Eastern Finland and Kuopio University Hospital, Kuopio, Finland; 102 deCODE Genetics, Amgen inc., Reykjavik, Iceland; 103 Atherosclerosis Research Unit, Department of Medicine Solna, Karolinska Institutet, Stockholm, Sweden; 104 Center for Molecular Medicine, Karolinska University Hospital, Stockholm, Sweden; 105 Translational Gerontology Branch, National Institute on Aging, Baltimore, Maryland, United States of America; 106 Department of Cardiology, Leiden University Medical Center, Leiden, The Netherlands; 107 Institute of Cell and Molecular Biology, Department of Biotechnology, University of Tartu, Tartu, Estonia; 108 University of Helsinki, Helsinki, Finland; 109 Department of Oncology, University of Cambridge, Cambridge, United Kingdom; 110 Department of Internal Medicine, Section of Geriatric Medicine, Academic Medical Center, Amsterdam, The Netherlands; 111 Department of Medical Genetics, University Medical Center Utrecht, Utrecht, Netherlands; 112 University of Groningen, University Medical Center Groningen, Department of Endocrinology, Groningen, The Netherlands; 113 Transplantation Laboratory, Haartman Institute, University of Helsinki, Helsinki, Finland; 114 Division of Endocrinology, Boston Children's Hospital, Boston, Massachusetts, United States of America; 115 Divisions of Genetics and Endocrinology and Program in Genomics, Boston's Children's Hospital, Boston, Massachusetts, United States of America; 116 Centre for Population Health Sciences, Usher Institute of Population Health Sciences and Informatics, University of Edinburgh, Edinburgh, Scotland; 117 DZHK (German Centre for Cardiovascular Research), partner site Hamburg/Kiel/Lübeck, Lübeck, Germany; 118 Institut für Integrative und Experimentelle Genomik, Universität zu Lübeck, Lübeck, Germany; 119 Centre for Global Health Research, Usher Institute of Population Health Sciences and Informatics, University of Edinburgh, Edinburgh, Scotland; 120 MRC Unit for Lifelong Health & Ageing at UCL, London, United Kingdom; 121 Queensland Brain Institute, The University of Queensland, Brisbane, Queensland, Australia; 122 Rush Alzheimer's Disease Center, Rush University Medical Center, Chicago, Illinois, United States of America; 123 Ealing Hospital NHS Trust, Middlesex, United Kingdom; 124 University of Groningen, University Medical Center Groningen, Department of Medicine, Groningen, Netherlands; 125 Centro Cardiologico Monzino, IRCCS, Milan, Italy; 126 Dipartimento di Scienze Farmacologiche e Biomolecolari, Università di Milano, Milan, Italy; 127 Department of Genetics, Texas Biomedical Research Institute, San Antonio, Texas, United States of America; 128 Genomics Research Centre, Institute of Health and Biomedical Innovation, Queensland University of Technology, Brisbane, Queensland, Australia; 129 Department of Pediatrics, University of California San Diego, La Jolla, California, United States of America; 130 Department of Medical Genetics, University of Lausanne, Lausanne, Switzerland; 131 University of Leipzig, IFB Adiposity Diseases, Leipzig, Germany; 132 University of Leipzig, Department of Medicine, Leipzig, Germany; 133 University Rennes 1, Rennes, France; 134 Medical Genomics and Metabolic Genetics Branch, National Human Genome Research Institute, NIH, Bethesda, Maryland, United States of America; 135 University of Groningen, University Medical Center Groningen, The LifeLines Cohort Study, Groningen, The Netherlands; 136 Los Angeles BioMedical Resesarch Institute at Harbor-UCLA Medical Center, Torrance, California, United States of America; 137 Department of Internal Medicine I, Ulm University Medical Centre, Ulm, Germany; 138 Universiy of Maryland School of Medicine, Department of Neurology, Baltimore, Maryland, United States of America; 139 EMGO Institute for Health and Care Research, VU University Medical Center, Amsterdam, The Netherlands; 140 Department of Human Nutrition, Wageningen University, Wageningen, The Netherlands; 141 Department of Dietetics-Nutrition, Harokopio University, Athens, Greece; 142 NorthShore University HealthSystem, Evanston, Illinois, United States of America; 143 University of Chicago, Chicago, Illinois, United States of America; 144 Department of Nutrition and Dietetics, School of Health Science and Education, Harokopio University, Athens, Greece; 145 Institute of Clinical Chemistry and Laboratory Medicine, University Medicine Greifswald, Greifswald, Germany; 146 Division of Cardiovascular Epidemiology, Institute of Environmental Medicine, Karolinska Institutet, Stockholm, Sweden; 147 Hypertension and Related Disease Centre, AOU-University of Sassari, Sassari, Italy; 148 Kaiser Permanente, Division of Research, Oakland, California, United States of America; 149 The Department of Medicine, The Icahn School of Medicine at Mount Sinai, New York, New York, United States of America; 150 Department of Medicine III, Pathobiochemistry, University of Dresden, Dresden, Germany; 151 Research Unit of Molecular Epidemiology, Helmholtz Zentrum München—German Research Center for Environmental Health, Neuherberg, Germany; 152 Institute of Epidemiology II, Helmholtz Zentrum München—German Research Center for Environmental Health, Neuherberg, Germany; 153 German Center for Diabetes Research (DZD), Neuherberg, Germany; 154 Research Unit Hypertension and Cardiovascular Epidemiology, KU Leuven Department of Cardiovascular Sciences, University of Leuven, Leuven, Belgium; 155 Faculty of Health Sciences, University of Southern Denmark, Odense, Denmark; 156 Laboratory of Epidemiology and Population Sciences, National Institute on Aging, Bethesda, Maryland, United States of America; 157 National Institute on Aging, National Institutes of Health, Bethesda, Maryland, United States of America; 158 University of Groningen, University Medical Center Groningen, Department of Psychiatry, Groningen, The Netherlands; 159 Kuopio Research Institute of Exercise Medicine, Kuopio, Finland; 160 Institue of Biomedical & Clinical Science, University of Exeter, Exeter, United Kingdom; 161 QIMR Bergofer Medical Research Institute, Brisbane, Queensland, Australia; 162 Laboratory of Neurogenetics, National Institute on Aging, National Institutes of Health, Bethesda, Maryland, United States of America; 163 Department of Public Health and General Practice, Norwegian University of Science and Technology, Trondheim, Norway; 164 Department Vascular Medicine, Academic Medical Center, Amsterdam, The Netherlands; 165 Pathwest Laboratory Medicine of Western Australia, Nedlands, Western Australia,Australia; 166 School of Pathology and Laboratory Medicine, University of Western Australia, Nedlands, Western Australia, Australia; 167 School of Population Health, University of Western Australia, Nedlands, Western Australia, Australia; 168 Research Centre for Prevention and Health, Glostrup Hospital, Glostrup, Denmark; 169 Department of Pediatrics, University of Tampere School of Medicine, Tampere, Finland; 170 Department of Pediatrics,Tampere University Hospital, Tampere, Finland; 171 Hannover Unified Biobank, Hannover Medical School, Hannover, Germany; 172 Institute of Human Genetics, Hannover Medical School, Hanover, Germany; 173 Harvard Medical School, Boston, Massachusetts,United States of America; 174 Program in Translational NeuroPsychiatric Genomics, Department of Neurology, Brigham and Women’s Hospital, Boston, Massachusetts,United States of America; 175 Faculty of Health and Medical Sciences, University of Copenhagen, Copenhagen, Denmark; 176 Faculty of Medicine, University of Aalborg, Aalborg, Denmark; 177 Interuniversity Cardiology Institute of the Netherlands, Utrecht, The Netherlands; 178 Division of Medicine, Turku University Hospital, Turku, Finland; 179 Murdoch Children's Research Institute, Parkville, Victoria, Australia; 180 Department of Medicine, University of Turku, Turku, Finland; 181 William Harvey Research Institute, Barts and The London School of Medicine and Dentistry, Queen Mary University of London, London, United Kingdom; 182 Echinos Medical Centre, Echinos, Greece; 183 Clinical Gerontology Unit, Addenbrooke's Hospital, Cambridge, United Kingdom; 184 Department of Health, National Institute for Health and Welfare, Helsinki, Finland; 185 Department of Internal Medicine II—Cardiology, University of Ulm Medical Center, Ulm, Germany; 186 Department of Public Health, Faculty of Medicine, University of Split, Split, Croatia; 187 Department of Medicine A, University Medicine Greifswald, Greifswald, Germany; 188 Department of Epidemiology and Public Health, UCL, London, United Kingdom; 189 Department of Public Health and Caring Sciences, Geriatrics, Uppsala University, Uppsala, Sweden; 190 Chair of Nephrology, Università Vita Salute San Raffaele, Segrate (Milan), Italy; 191 Genomics of Renal Disease and Hypertension Unit, IRCCS San Raffaele Scientific Institute, Segrate (Milan), Italy; 192 Department of Biological Sciences, University of Calgary, Calgary, Alberta, Canada; 193 Diabetes Prevention Unit, National Institute for Health and Welfare, Helsinki, Finland; 194 Department of Clinical Experimental Research, Rigshospitalet, Glostrup, Denmark; 195 Strangeways Research Laboratory Wort's Causeway, Cambridge, United Kingdom; 196 Lund University Diabetes Centre and Department of Clinical Science, Diabetes & Endocrinology Unit, Lund University, Malmö, Sweden; 197 Department of Medical Epidemiology and Biostatistics, Karolinska Institutet, Stockholm, Sweden; 198 School of Social and Community Medicine, University of Bristol, Bristol, United Kingdom; 199 Department of Biostatistics, University of Liverpool, Liverpool, United Kingdom; 200 Department of Paediatrics, University of Cambridge, Cambridge, United Kingdom; 201 Massachusetts General Hospital, Center for Human Genetic Research, Psychiatric and Neurodevelopmental Genetics Unit, Boston, Massachusetts, United States of America; 202 Department of Kinesiology, Laval University, Québec City, Québec, Canada; 203 Institute of Nutrition and Functional Foods, Laval University, Québec City, Québec, Canada; 204 Center for Biomedicine, European Academy Bozen/Bolzano (EURAC), Bolzano, Italy, Affiliated Institute of the University of Lübeck, Lübeck, Germany; 205 Department of Children, Young People and Families, National Institute for Health and Welfare, Helsinki, Finland; 206 Department of Obstetrics and Gynecology, Medical Research Center Oulu, Oulu University Hospital and University of Oulu, Oulu, Finland; 207 Department of Psychiatry, Washington University School of Medicine, St. Louis, Missouri, United States of America; 208 Human Genomics Laboratory, Pennington Biomedical Research Center, Baton Rouge, Louisiana, United States of America; 209 Science for Life Laboratory, Karolinska Institutet, Stockholm, Sweden; 210 Department of Molecular Epidemiology, Leiden University Medical Center, Leiden, The Netherlands; 211 Department of Medical Sciences, Uppsala University, Uppsala, Sweden; 212 National Heart and Lung Institute, Imperial College London, London, United Kingdom; 213 Anogia Medical Centre, Anogia, Greece; 214 DZHK (German Centre for Cardiovascular Research), partner site Greifswald, Greifswald, Germany; 215 School of Nutrition, Laval University, Québec City, Québec,Canada; 216 Department of Clinical Chemistry, Ulm University Medical Centre, Ulm, Germany; 217 Department of Clinical Medicine, Faculty of Health Sciences, University of Tromsø, Tromsø, Norway; 218 Department of Community Medicine, Faculty of Health Sciences, University of Tromsø, Tromsø, Norway; 219 VUMC, Department of Epidemiology and Biostatistics, Amsterdam, The Netherlands; 220 Department of Cardiology, Division Heart and Lungs, University Medical Center Utrecht, Utrecht, Netherlands; 221 Durrer Center for Cardiogenetic Research, Interuniversity Cardiology Institute Netherlands-Netherlands Heart Institute, Utrecht, The Netherlands; 222 Institute of Cardiovascular Science, University College London, London, United Kingdom; 223 Department of Epidemiology, University Medical Center, Utrecht, The Netherlands; 224 Diabetes and Obesity Research Institute, Cedars-Sinai Medical Center, Los Angeles, California, United States of America; 225 Imperial College London, London, United Kingdom; 226 Lee Kong Chian School of Medicine, Singapore, Singapore; 227 Nanyang Technological University, Singapore, Singapore; 228 Human Genetics Center and Institute of Molecular Medicine, University of Texas Health Science Center, Houston, Texas, United States of America; 229 Department of Medicine III, University of Dresden, Medical Faculty Carl Gustav Carus, Dresden, Germany; 230 Imperial College Healthcare NHS Trust, London, United Kingdom; 231 University of Sassari, Sassari, Italy; 232 Institute of Biomedical Technologies, National Institute of Research, Segrate-Milano, Italy; 233 Department of General Practice and Primary Health Care, University of Helsinki, Helsinki, Finland; 234 Rush Institute for Healthy Aging and Department of Internal Medicine, Rush University Medical Center, Chicago, Illinois, United States of America; 235 Durrer Center for Cardiogenetic Research, Amsterdam, The Netherlands; 236 Robertson Center for Biostatistics, University of Glasgow, Glasgow, United Kingdom; 237 Department of Public Health & Clinical Medicine, Umeå University Hospital, Umeå, Sweden, Umeå, Sweden; 238 Department of Nutrition, Harvard School of Public Health, Boston, Massachusetts, United States of America; 239 Department of Medicine, Karolinska Institutet, Stockholm, Sweden; 240 Deutsches Herzzentrum München, Technische Universität München, München, Germany; 241 Channing Division of Network Medicine, Department of Medicine, Brigham and Women’s Hospital and Harvard Medical School, Boston, Massachusetts, United States of America; 242 Medical Research Center Oulu, Oulu University Hospital, and University of Oulu, Oulu, Finland; 243 Department of Pulmonary Physiology and Sleep Medicine, Sir Charles Gairdner Hospital, Nedlands, Western Australia, Australia; 244 Department of Clinical Physiology, Tampere University Hospital, Tampere, Finland; 245 Department of Clinical Physiology, University of Tampere School of Medicine, Tampere, Finland; 246 Children's Hospital, Helsinki University Hospital and University of Helsinki, Helsinki, Finland; 247 Cardiovascular Research Center and Cardiology Division, Massachusetts General Hospital, Boston, Massachusetts, United States of America; 248 Center for Human Genetics Research, Massachusetts General Hospital, Boston, Massachusetts, United States of America; 249 Radboud university medical center, Radboud Institute for Health Sciences, Department of Urology, Nijmegen, The Netherlands; 250 National Institute for Health and Welfare, Helsinki, Finland; 251 University of Helsinki and Helsinki University Central Hospital, Department of Medicine and Abdominal Center: Endocrinology, Helsinki, Finland; 252 Minerva Foundation Institute for Medical Research, Helsinki, Finland; 253 Department of Physiology, Institute of Biomedicine, University of Eastern Finland, Kuopio Campus, Kuopio, Finland; 254 Department of Clinical Chemistry, University of Tampere School of Medicine, Tampere, Finland; 255 Department of Clinical Chemistry, Fimlab Laboratories and School of Medicine, University of Tampere, Tampere, Finland; 256 Department of Medicine, Université de Montréal, Montréal, Québec, Canada; 257 Stanford University, Stanford, California, United States of America; 258 Primary Health Care Unit, Institute of Public Health and Clinical Nutrition, School of Medicine, University of Eastern Finland, Kuopio, Finland; 259 Primary Health Care Unit, Kuopio University Hospital, Kuopio, Finland; 260 Department of Medicine, Stanford University School of Medicine, Stanford, California, United States of America; 261 Geriatrics Research and Education Clinical Center, Baltimore Veterans Administration Medical Center, Baltimore, Maryland, United States of America; 262 Department of Surgery, University Medical Center Utrecht, Utrecht, Netherlands; 263 Department of Pediatrics, University of Iowa, Iowa City, Iowa, United States of America; 264 Department of Respiratory Medicine, Sir Charles Gairdner Hospital, Nedlands, Western Australia, Australia; 265 HUCH Heart and Lung Center, Division of Cardiology, Helsinki University Central Hospital, Helsinki, Finland; 266 University of Groningen, University Medical Center, Interdisciplinary Center Psychopathology and Emotion Regulation, Groningen, The Netherlands; 267 School of Public Health, University of Adelaide, Adelaide, South Australia, Australia; 268 Robinson Research Institute, University of Adelaide, Adelaide, South Australia, Australia; 269 Division of Epidemiology and Community Health, School of Public Health, University of Minnesota, Minneapolis, Minnesota, United States of America; 270 Department of Neurology, General Central Hospital, Bolzano, Italy; 271 Departments of Epidemiology and Health Services, University of Washington, Seattle, Washington, United States of America; 272 Group Health Research Institute, Group Health Cooperative, Seatte, Washington, United States of America; 273 Department of Clinical Physiology and Nuclear Medicine, Turku University Hospital, Turku, Finland; 274 Research Centre of Applied and Preventive Cardiovascular Medicine, University of Turku, Turku, Finland; 275 Department of Clinical Physiology and Nuclear Medicine, Kuopio University Hospital, Kuopio, Finland; 276 Department of Medicine, Central Finland Central Hospital, Jyväskylä, Finland; 277 BHF Glasgow Cardiovascular Research Centre, University of Glasgow, Glasgow, United Kingdom; 278 Geriatric Research and Education Clinical Center, Vetrans Administration Medical Center, Baltimore, Maryland, United States of America; 279 MRC Integrative Epidemiology Unit, School of Social and Community Medicine, University of Bristol, Bristol, United Kingdom; 280 Institute of Preventive Medicine, Bispebjerg and Frederiksberg Hospital, The Capital Region, Frederiksberg, Denmark; 281 R & D VitaK Group, Maastricht University, Maastricht, The Netherlands; 282 Geriatric Unit, Azienda Sanitaria Firenze (ASF), Florence, Italy; 283 Faculty of Medicine, University of Iceland, Reykjavik, Iceland; 284 Centre for Vascular Prevention, Danube-University Krems, Krems, Austria; 285 Instituto de Investigacion Sanitaria del Hospital Universitario La Paz (IdiPAZ), Madrid, Spain; 286 Diabetes Research Group, King Abdulaziz University, Jeddah, Saudi Arabia; 287 Department of Public Health and Clinical Nutrition, University of Eastern Finland, Finland; 288 Research Unit, Kuopio University Hospital, Kuopio, Finland; 289 Department of Human Genetics, Radboud university medical center, Nijmegen, The Netherlands; 290 Department of Internal Medicine, University Hospital Lausanne (CHUV) and University of Lausanne, Lausanne, Switzerland; 291 Institute of Cellular Medicine, Newcastle University, Newcastle, United Kingdom; 292 The Cohorts for Heart and Aging Research in Genomic Epidemiology Consortium; 293 The DIAbetes Genetics Replication And Meta-analysis Consortium; 294 The Global Lipids Genetics Consortium; 295 The Global Blood Pressure Genetics Consortium; 296 The International Consortium for Blood Pressure; 297 The Meta-Analyses of Glucose and Insulin-related traits Consortium; 298 Department of Nutrition, Gillings School of Global Public Health, University of North Carolina, Chapel Hill, North Carolina, United States of America; 299 Carolina Population Center, University of North Carolina at Chapel Hill, Chapel Hill, North Carolina, United States of America; 300 National Heart, Lung, and Blood Institute, National Institute of Health, Bethesda, Maryland, United States of America; 301 NIHR Cambridge Biomedical Research Centre, Institute of Metabolic Science Addenbrooke’s Hospital, Cambridge, United Kingdom; 302 University of Cambridge Metabolic Research Laboratories, Institute of Metabolic Science Addenbrooke’s Hospital, Cambridge, United Kingdom; 303 Princess Al-Jawhara Al-Brahim Centre of Excellence in Research of Hereditary Disorders (PACER-HD), King Abdulaziz University, Jeddah, Saudi Arabia; 304 Finnish Institute for Molecular Medicine (FIMM), Helsinki University, Helsinki, Finland; 305 Department of Epidemiology, Harvard School of Public Health, Boston, Massachusetts, United States of America; 306 Department of Medicine, Division of Cardiovascular Medicine, Stanford University School of Medicine, Stanford, California, United States of America; 307 Department of Epidemiology and Popualtion Health, Albert Einstein College of Medicine, Bronx, New York, United States of America; 308 Oxford NIHR Biomedical Research Centre, Oxford, United Kingdom; 309 Department of Genetics, University of North Carolina, Chapel Hill, North Carolina, United States of America; 310 Population Health Research Institute, St George's, University of London, London, United Kingdom; 311 Center for Medical Systems Biology, Leiden, The Netherlands; 312 Carolina Center for Genome Sciences and Department of Epidemiology, University of North Carolina at Chapel Hill, Chapel Hill, North Carolina, United States of America; 313 The Genetics of Obesity and Related Metabolic Traits Program, The Icahn School of Medicine at Mount Sinai, New York, New York, United States of America; 314 The Mindich Child Health and Development Institute, The Icahn School of Medicine at Mount Sinai, New York, New York, United States of America; National Institute of Health, National Human Genome Research Institute, UNITED STATES

## Abstract

Genome-wide association studies (GWAS) have identified more than 100 genetic variants contributing to BMI, a measure of body size, or waist-to-hip ratio (adjusted for BMI, WHR_adjBMI_), a measure of body shape. Body size and shape change as people grow older and these changes differ substantially between men and women. To systematically screen for age- and/or sex-specific effects of genetic variants on BMI and WHR_adjBMI_, we performed meta-analyses of 114 studies (up to 320,485 individuals of European descent) with genome-wide chip and/or Metabochip data by the Genetic Investigation of Anthropometric Traits (GIANT) Consortium. Each study tested the association of up to ~2.8M SNPs with BMI and WHR_adjBMI_ in four strata (men ≤50y, men >50y, women ≤50y, women >50y) and summary statistics were combined in stratum-specific meta-analyses. We then screened for variants that showed age-specific effects (G x AGE), sex-specific effects (G x SEX) or age-specific effects that differed between men and women (G x AGE x SEX). For BMI, we identified 15 loci (11 previously established for main effects, four novel) that showed significant (FDR<5%) age-specific effects, of which 11 had larger effects in younger (<50y) than in older adults (≥50y). No sex-dependent effects were identified for BMI. For WHR_adjBMI_, we identified 44 loci (27 previously established for main effects, 17 novel) with sex-specific effects, of which 28 showed larger effects in women than in men, five showed larger effects in men than in women, and 11 showed opposite effects between sexes. No age-dependent effects were identified for WHR_adjBMI_. This is the first genome-wide interaction meta-analysis to report convincing evidence of age-dependent genetic effects on BMI. In addition, we confirm the sex-specificity of genetic effects on WHR_adjBMI_. These results may provide further insights into the biology that underlies weight change with age or the sexually dimorphism of body shape.

## Author Summary

Adult body size and body shape differ substantially between men and women and change over time. More than 100 genetic variants that influence body mass index (measure of body size) or waist-to-hip ratio (measure of body shape) have been identified. While there is evidence that some genetic loci affect body shape differently in men than in women, little is known about whether genetic effects differ in older compared to younger adults, and whether such changes differ between men and women. Therefore, we conducted a systematic genome-wide search, including 114 studies (>320,000 individuals), to specifically identify genetic loci with age- and or sex-dependent effects on body size and shape. We identified 15 loci of which the effect on BMI was different in older compared to younger adults, whereas we found no evidence for loci with different effects in men compared to women. The opposite was seen for body shape as we identified 44 loci of which the effect on waist-to-hip ratio differed between men and women, but no difference between younger and older adults were observed. Our observations may provide new insights into the biology that underlies weight change with age or the sexual dimorphism of body shape.

## Introduction

Body size and shape are independent risk factors for morbidity and mortality [1–6]. They change as people grow older and these changes differ substantially between men and women [7–12]. Subtle sexual dimorphisms are already apparent during early childhood, but differences become more apparent during puberty due, at least in part, to the increasing influence of sex steroid hormones [12–14]. After puberty, sex-differences are largely maintained over the adult life-course. As women age a decline in sex steroid hormones, which coincides with menopause, affects their body shape and composition, resulting in a more android fat distribution [8, 12, 15]. When younger, women tend towards an hourglass body shape with gynoid fat distribution, storing proportionally more fat at thighs and hip than around the waist [12, 16, 17]. At a later age, often after menopause, women’s fat storage shifts more upwards around the waist [12, 16, 17]. In men, changes in body fat distribution are subtler than in women, showing a slow but steady increase in waist circumference with age [12]. Thus, after the menopause, the sex-differences in body shape between men and women decrease [12].

This intricate interplay between age and sex on body size and shape is driven by underlying biological processes, involving environmental and genetic factors [7–12, 15]. Elucidating sex- and age-specific genetic effects on body size and shape may provide insights into the biological processes that are involved in the regulation of body weight and fat distribution.

More than 100 genetic loci have been identified for body mass index (BMI), a measure for body size, and for waist-to-hip ratio adjusted for BMI (WHR_adjBMI_), a measure of body shape, most of which were identified through our own work in the Genetic Investigation of ANthropometric Traits (GIANT) Consortium [18, 19]. In a recent sex-stratified genome-wide association meta-analysis (up to 133,723 individuals in discovery stage), we searched for variants with sex-specific effects on BMI and WHR_adjBMI_ and identified several loci for which the association with WHR_adjBMI_ differed between men and women, whereas no such loci were observed for BMI [10]. However, so far, no GWAS efforts have aimed to identify genetic loci that contribute to differences in body size and shape observed in younger versus older adults, particularly across the menopausal period in women.

We conducted a genome-wide search for loci that exhibit age- and/or sex-specific differences in BMI and WHR_adjBMI_. For this, we utilized study-specific genome-wide association statistics separately by sex and by two age groups in each of the studies participating in the GIANT consortium. The two age groups focus on those below and above 50 years of age, as this cut-off coincides with the average age at which women transition through menopause and experience changes in body fat distribution [20–25]. We hypothesize that genetic loci may contribute to the observed differences in body size/shape before age 50y and after age 50y, and that these differences may be sex-specific.

## Results

### Stratified GWAS identifies age- and sex-specific loci for BMI and WHR_adjBMI_

Our total sample comprised up to 320,485 adults (≥18y) of European ancestry from 114 studies with genome-wide array data imputed to the HapMap reference or genotyped Illumina Metabochip array data including up to 2.8 million autosomal variants. Details on study-specific analyses, genotyping methods and phenotypic descriptives are given in **S1–S3 Tables**. To systematically search for genetic loci that influence body size or shape in an age- and sex-specific manner, we first conducted study-specific GWA analyses for BMI and WHR_adjBMI_ by four strata (men ≤50y, men >50y, women ≤50y, women >50y), and subsequently performed stratified meta-analyses (comprising up to 50,095 men ≤50y, 93,201 men >50y, 70,692 women ≤50y, and 106,497 women >50y) and derived pooled stratum-specific association results (*P_men≤50_*, *P*_*men>50*_, *P_women≤50_*, *P*_*women>50*_) for each trait. This strategy allowed us to test for three types of interactions: (1) SNPs that demonstrate age-specific effects (SNP x AGE, *P*_*agediff*_), (2) SNPs that show sex-specific effects (SNP x SEX, *P*_*sexdiff*_), and (3) SNPs that show age-specific effects that differ between men and women (SNP x AGE x SEX, *P*_*agesexdiff*_). We first performed genome-wide screens using an *a priori* filter; i.e. we examined interaction effects on SNPs that showed evidence of an overall main-effect association (*P*_*Overall*_ < 10^−5^). This screen is known to have better power to identify loci with age- or sex-specific effects that are directionally concordant [10, 26]. In a second screen, we examined interaction effects for all SNPs, irrespective of their main-effect association, which allows identification of loci with opposite effect direction in older vs younger adults or in men vs women.

**Fig 1:**
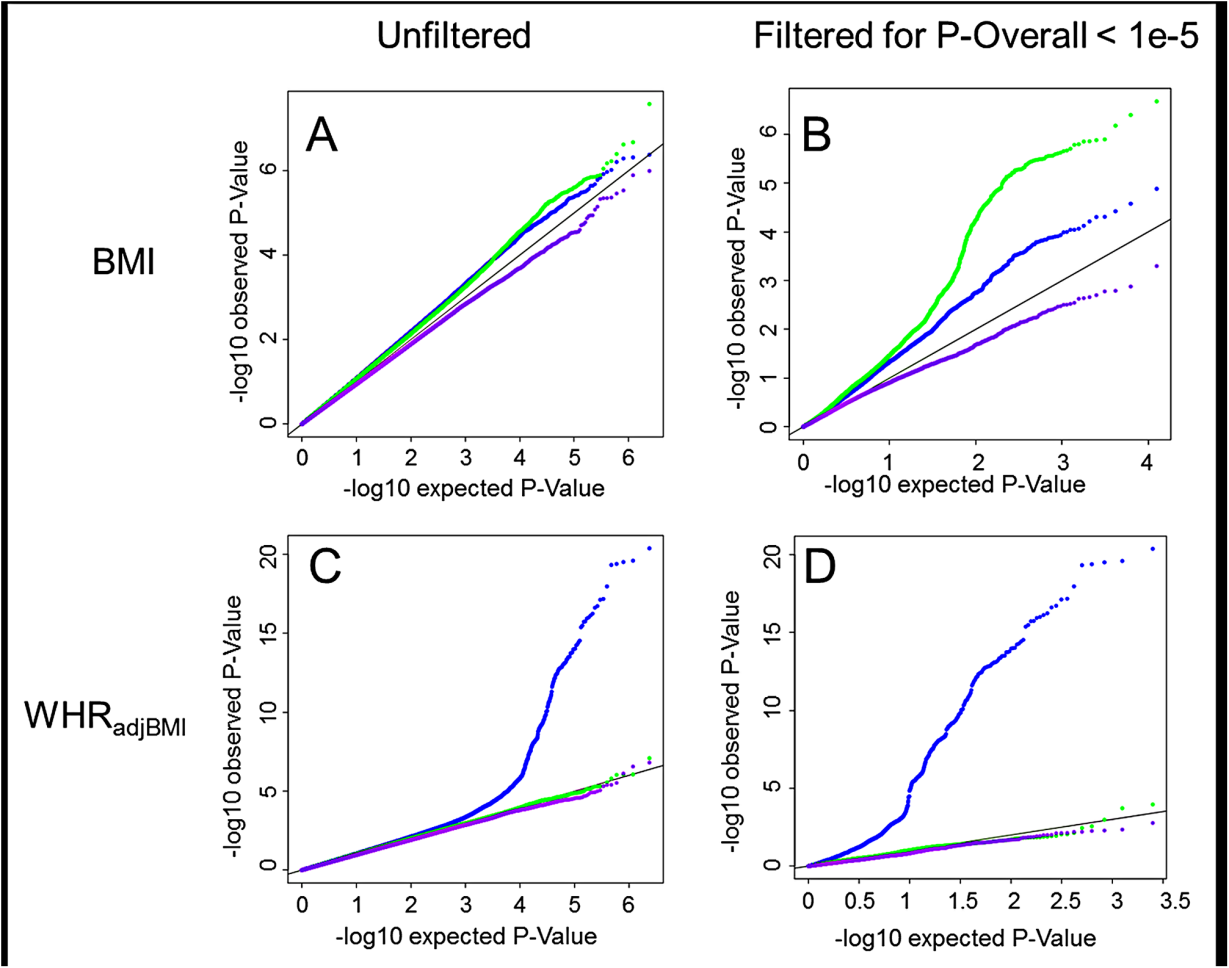
Interaction QQ plots. Quantile-Quantile plots showing P-Values for age-difference (*P*_*agediff*_, green), sex-difference (*P*_*sexdiff*_, blue) and age- and sex-difference (*P*_*agesexdiff*_, purple). For BMI the P-Values are depicted for all SNPs genome-wide (A) as well as for a limited subset of SNPs that survived pre-filtering on the overall association with BMI, *P*_*Overall*_ < 1x10^-5^ (B). For WHR_adjBMI_ the P-Values are depicted for all SNPs genome-wide (C) as well as for a limited subset of SNPs that survived pre-filtering on the overall association with WHR_adjBMI_, *P*_*Overall*_ < 1x10^-5^ (D).

As such, 15 loci with age-specific effects for BMI and 44 loci with sex-specific effects for WHR_adjBMI_ reached significance after accounting for multiple testing (controlling false-discovery rate, FDR <5%) (**Figs 1** and **S1**). No loci were identified with evidence for three way SNP x AGE x SEX interaction.

In addition to the stratum-specific meta-analyses, we performed (a) a *main effect* meta-analysis that combined the four pooled effect estimates (one from each stratum), providing results for the *overall association* (*P*_*Overall*_), assuming effects in age- and sex-groups are the same, and (b) a *joint (main + interaction) meta-analysis approach* (*P*_*joint*_) allowing for simultaneous testing of overall association, SNP-by-age and SNP-by-sex interactions [27]. These two screens revealed 83 novel loci of which the association with BMI or WHR_adjBMI_ reached genome-wide significance (P<5x10^-8^) (**S2 Fig**). This extended discovery is enabled through power augmentation achieved by simultaneously testing main and interaction effects, and/or by accounting for potentially different effects of age and sex on the respective phenotype in the four strata.

### BMI-novel loci with differential effects in younger and older individuals

**Fig 2:**
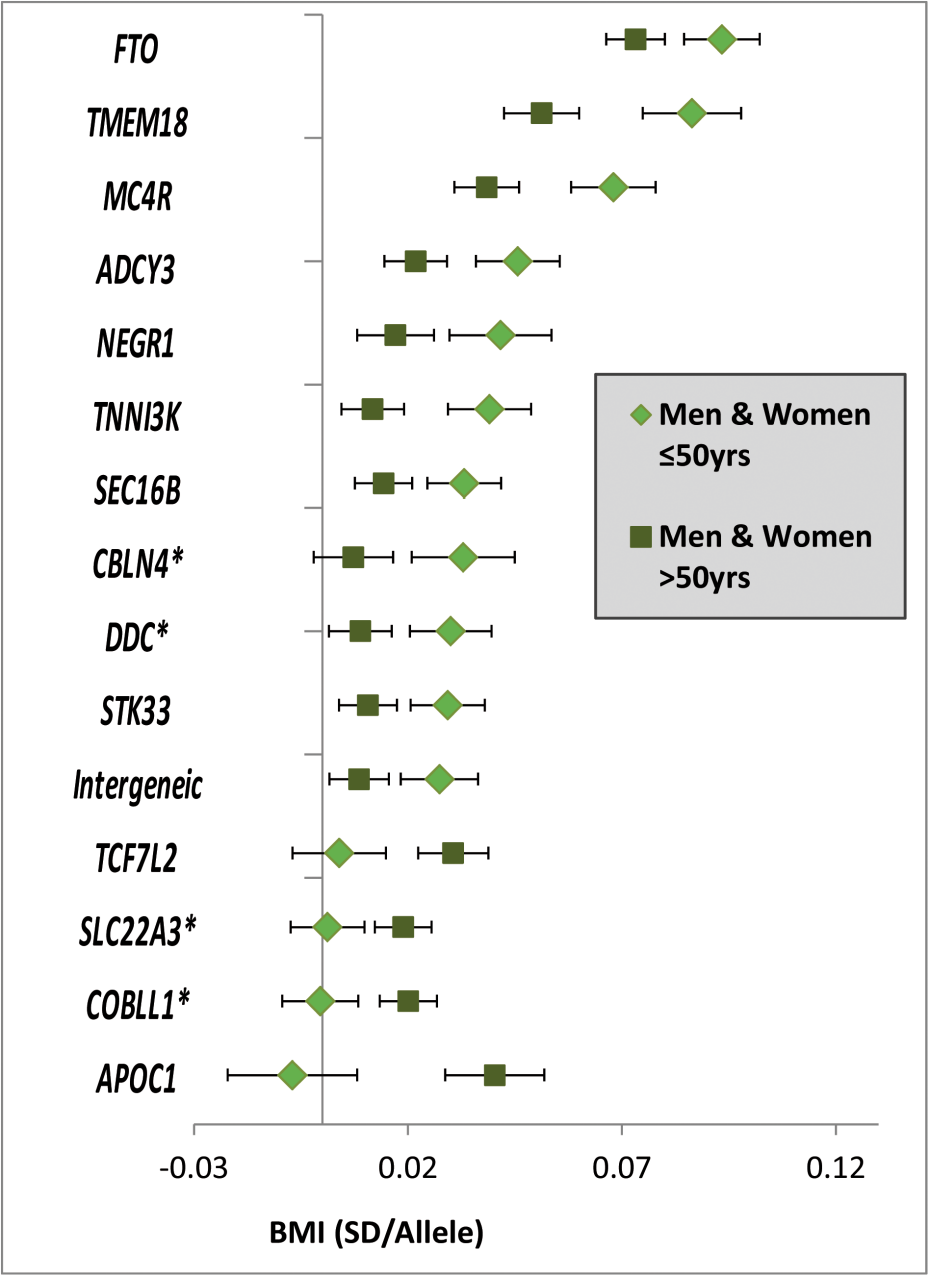
Age-dependent BMI loci. Effect estimates (beta ±95CI) per standard deviation in BMI and risk allele for loci showing age-differences in men & women ≤50y compared to men & women >50y. Loci are ordered by greater magnitude of effect in men & women ≤50y compared to men & women >50y. (95%CI: 95% confidence interval; BMI: body mass index; SD: standard deviation, *Newly identified loci).

Among the 15 loci with significantly different effects (at 5% FDR) on BMI in the younger versus the older individuals, four were novel (near *COBLL1*, *DDC*, *SLC22A3* and *CBLN4)* and 11 were previously established as BMI loci in large-scale *main effect* GWA meta-analyses (near *NEGR1*, *TNNI3K*, *SEC16B*, *TMEM18*, *ADCY3*, *AC016194*.*1*, *TCF7L2*, *STK33*, *FTO*, *MC4R*, *APOC1)* (**S3 Fig** and **Tables 1** and **S4**) [19, 28]. Eleven of the 15 age-dependent BMI loci (73%, *P*_*binomial*_ = 0.06 for divergence from 50%) showed stronger effects in the younger than in the older group, while the four remaining loci had effects that were more pronounced in the older than in the younger group (**Figs 2** and **S4**). We did not identify BMI-associated loci that showed effects in opposite direction between the younger versus the older group, nor did we find any sex-specific BMI effects. A sensitivity analysis excluding studies with self-report BMI found similar results (**S5 Fig**).

**Table 1:** Fifteen BMI loci showing significant age-differences in adults ≤50y compared to adults >50y. The table shows the age-group specific (sex-combined) results, ordered by largest to smallest effect in adults ≤50y. All loci were detected by the screen on age-difference that included the a-priori filter on *P*_*Overall*_ < 10^−5^. The age- and sex-specific results (four strata) and more detailed information on the loci are given in **S4 Table**.

							Age ≤ 50y			Age > 50y			
SNP	Novel Locus^a^	Nearest Gene	Chr	Pos	Alleles^b^EA/OA	EAF	β	P	N	β	P	N	P_Agediff_
rs9936385		*FTO*	16	52376670	C/T	39%	0.093	4.5E-95	115,354	0.073	1.0E-97	197,478	1.6E-04
rs2867125		*TMEM18*	2	612827	C/T	83%	0.086	6.1E-49	112,934	0.051	2.3E-30	195,579	4.0E-07
rs12955983		*MC4R*	18	56023969	G/A	28%	0.068	1.7E-41	114,448	0.038	2.0E-23	196,590	6.7E-07
rs6737082		*ADCY3*	2	24991544	C/A	47%	0.046	6.3E-20	92,191	0.022	5.4E-09	162,112	4.7E-05
rs2821248		*NEGR1*	1	72348148	A/G	83%	0.042	8.4E-12	106,067	0.017	1.9E-04	188,322	6.2E-04
rs1514174		*TNNI3K*	1	74765651	C/T	43%	0.039	3.0E-15	92,120	0.012	1.7E-03	161,764	2.8E-06
rs591120		*SEC16B*	1	176169376	C/G	20%	0.033	4.9E-14	115,337	0.014	2.8E-05	197,481	3.1E-04
rs11908421	yes	*CBLN4*	20	53813074	T/C	81%	0.033	8.7E-08	92,575	0.007	1.2E-01	162,284	4.3E-04
rs4947644	yes	*DDC*	7	50586370	T/C	51%	0.030	7.7E-10	91,980	0.009	1.7E-02	158,555	2.5E-04
rs10840060		*STK33*	11	8456621	C/A	50%	0.029	3.8E-11	110,697	0.011	2.0E-03	187,808	4.0E-04
rs1459180		Intergeneic	8	77144822	G/T	58%	0.027	3.1E-09	112,913	0.009	1.6E-02	190,729	6.0E-04
rs17747324		*TCF7L2*	10	114742493	T/C	77%	0.004	4.8E-01	111,572	0.031	2.6E-13	193,773	4.7E-05
rs3127574	yes	*SLC22A3*	6	160711360	C/G	51%	0.001	7.9E-01	113,057	0.019	2.3E-08	195,472	6.8E-04
rs3769885	yes	*COBLL1*	2	165300636	A/G	48%	-0.001	9.1E-01	107,703	0.020	3.9E-09	192,513	1.1E-04
rs4420638		*APOC1*	19	50114786	A/G	82%	-0.007	3.6E-01	83,196	0.040	8.9E-12	152,014	2.1E-07

Chr: Chromosome; Pos: position; EAF: Effect Allele Frequency; EA: Effect allele; OA: Other allele

^a^ ‘Yes’ if the locus is mentioned as BMI locus for the first time

^b^ Effect allele is according to the BMI increasing allele according to the associated sex.

### WHR_adjBMI_–additional genetic loci contribute to differences between men and women

**Fig 3:**
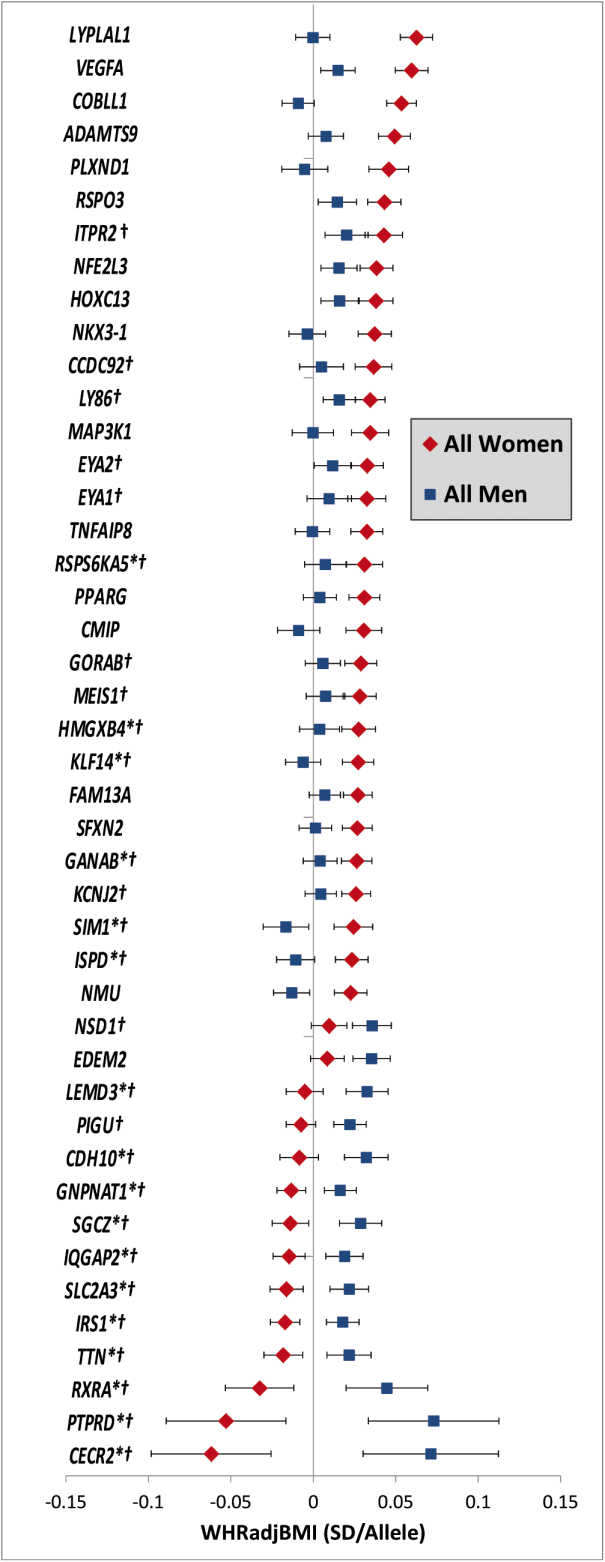
Sex-dependent WHR_adjBMI_ loci. Effect estimates (beta ± 95CI) per standard deviation in WHR_adjBMI_ and risk allele for loci showing sex-differences in women compared to men. Loci are ordered by greater magnitude of effect in women compared to men. (95%CI: 95% confidence interval; SD: standard deviation. *Newly identified loci. † Newly identified sex-differences)

Unlike for BMI, no WHR_adjBMI_-associated loci with significant difference between the age-groups were observed. Yet, 44 loci showed significantly different effects on WHR_adjBMI_ between women and men of which 17 loci were novel (near *TTN*, *IRS1*, *CDH10*, *IQGAP2*, *SIM1*, *ISPD*, *KLF14*, *SGCZ*, *PTPRD*, *RXRA*, *GANAB*, *SLC2A3*, *LEMD3*, *GNPNAT1*, *RPS6KA5*, *CECR2*, *HMGXB4)* and 27 loci had been previously established in *main-effect* GWA meta-analyses for WHR_adjBMI_ (**S6 Fig** and **Tables 2** and **S5**). Of the 27 previously established WHR_adjBMI_ loci, sex-differences had already been reported for 17 loci [10, 29] *[18]*. Our genome-wide screen established sex-specific effects for an additional 10 of the previously established loci with a main-effect on WHR_adjBMI_ (near *GORAB*, *LY86*, *ITPR2*, *PIGU*, *EYA2*, *KCNJ2*, *MEIS*, *EYA1*, *CCDC92*, *NSD1*). Of the 44 sex-specific loci, 11 loci showed opposite effect directions in women versus men and 33 showed a significant effect in one and a smaller or no effect in the other sex. Consistent with previous observations, almost all of these 33 loci (28 out of the 33, *P*_*binomial*_ = 3.3x10^-5^) showed more pronounced effects in women than in men (**Figs 3** and **S7**). Again, a sensitivity analysis excluding studies with self-report waist and hip circumference found similar results (**S8 Fig**).

**Table 2:** Forty-four WHR_adjBMI_ loci showing significant sex-differences. The table shows the sex-specific (age-group combined) results, ordered by largest, positive effect in women to largest, negative effect in women. The age- and sex-specific results (four strata), more detailed information on the loci and on the screens for which they were detected are given in **S5 Table**.

								Women			Men			
SNP	NovelLocus^a^	Novel Sexdiff^b^	NearestGene	Chr	Pos	Alleles^c^EA/OA	EAF	β	P	N	β	P	N	P_Sexdiff_
rs2820443			*LYPLAL1*	1	217820132	T/C	72%	0.063	5.2E-36	111,691	0.000	9.8E-01	93,780	1.1E-18
rs998584			*VEGFA*	6	43865874	A/C	48%	0.060	3.1E-32	109,533	0.015	5.0E-03	87,177	5.0E-10
rs6717858			*COBLL1*	2	165247907	T/C	59%	0.054	2.7E-31	110,110	-0.009	7.2E-02	90,259	4.2E-21
rs4616635			*ADAMTS9*	3	64677315	C/G	72%	0.049	4.0E-23	114,021	0.008	1.5E-01	93,679	7.5E-09
rs2811434			*PLXND1*	3	130822305	T/G	79%	0.046	8.8E-14	91,914	-0.005	4.7E-01	66,742	3.0E-08
rs1936811			*RSPO3*	6	127425553	T/A	61%	0.043	5.6E-17	91,862	0.015	1.4E-02	67,436	2.0E-04
rs10743579		yes	*ITPR2*	12	26352412	A/C	25%	0.043	1.4E-13	91,035	0.020	2.3E-03	67,178	8.7E-03
rs6958350			*NFE2L3*	7	25838458	T/C	25%	0.038	4.8E-14	114,759	0.016	4.9E-03	91,294	2.1E-03
rs1443512			*HOXC13*	12	52628951	A/C	24%	0.038	3.0E-13	114,486	0.016	5.8E-03	88,811	3.8E-03
rs7830933			*NKX2-6*	8	23659269	A/G	77%	0.037	4.4E-13	116,052	-0.004	5.3E-01	93,504	4.8E-08
rs11057396		yes	*CCDC92*	12	122985015	A/C	67%	0.037	1.2E-10	78,489	0.005	4.6E-01	53,789	2.6E-04
rs1294404		yes	*LY86*	6	6680021	A/G	61%	0.035	3.0E-14	116,324	0.016	1.4E-03	92,668	4.3E-03
rs9687846			*MAP3K1*	5	55897651	A/G	19%	0.035	1.9E-09	116,005	0.000	9.8E-01	93,710	3.5E-05
rs6018158		yes	*EYA2*	20	44971841	T/C	41%	0.033	7.5E-11	93,476	0.012	3.9E-02	67,612	5.0E-03
rs745578		yes	*EYA1*	8	72628878	A/G	24%	0.033	2.9E-08	92,963	0.010	1.6E-01	67,179	9.3E-03
rs1045241			*TNFAIP8*	5	118757185	C/T	71%	0.033	4.8E-11	116,314	0.000	9.3E-01	93,754	3.4E-06
rs7492628	yes	yes	*RPS6KA5*	14	90616889	G/C	30%	0.031	2.3E-08	91,645	0.007	2.5E-01	66,029	3.9E-03
rs17819328			*PPARG*	3	12464342	G/T	43%	0.031	8.5E-11	109,626	0.004	4.3E-01	88,650	7.9E-05
rs12443634			*CMIP*	16	80081775	A/C	29%	0.031	3.2E-08	93,188	-0.009	1.8E-01	66,051	2.4E-06
rs4656767		yes	*GORAB*	1	168646351	A/C	71%	0.029	5.3E-09	115,682	0.006	2.8E-01	91,023	1.3E-03
rs13029520		yes	*MEIS1*	2	66626466	T/C	40%	0.028	3.5E-08	86,851	0.007	2.1E-01	62,091	6.9E-03
rs2092029	yes	yes	*HMGXB4*	22	33982241	C/T	33%	0.028	1.4E-07	91,409	0.004	5.4E-01	63,601	2.7E-03
rs6971365	yes	yes	*KLF14*	7	130083021	C/T	30%	0.027	2.8E-08	116,043	-0.006	2.6E-01	92,416	2.9E-06
rs9991328			*FAM13A*	4	89932144	T/C	49%	0.027	1.2E-09	111,934	0.007	1.4E-01	92,564	1.7E-03
rs7917772			*SFXN2*	10	104477433	A/G	62%	0.027	6.2E-09	113,982	0.001	8.0E-01	90,756	1.3E-04
rs2956993	yes	yes	*GANAB*	11	62162738	G/T	38%	0.026	1.9E-08	111,837	0.004	4.2E-01	90,047	1.2E-03
rs8066985		yes	*KCNJ2*	17	65964940	A/G	51%	0.026	5.4E-09	114,268	0.005	3.5E-01	93,518	8.0E-04
rs17185536	yes	yes	*SIM1*	6	100727652	C/T	76%	0.024	4.3E-05	88,603	-0.017	1.9E-02	62,861	5.5E-06
rs9648211	yes	yes	*ISPD*	7	16056277	A/G	57%	0.023	3.6E-06	93,196	-0.011	6.9E-02	67,611	6.5E-06
rs3805389			*NMU*	4	56177507	A/G	28%	0.023	7.1E-06	110,897	-0.013	1.9E-02	88,609	1.1E-06
rs3088050		yes	*NSD1*	5	176659241	A/G	21%	0.010	7.8E-02	112,933	0.036	3.0E-09	91,432	1.1E-03
rs6088735			*EDEM2*	20	33209337	C/T	77%	0.009	1.0E-01	114,266	0.035	6.9E-10	90,782	4.0E-04
rs7307410	yes	yes	*LEMD3*	12	63828845	C/G	26%	-0.005	3.6E-01	89,227	0.033	5.0E-07	65,085	7.5E-06
rs6088552		yes	*PIGU*	20	32690152	G/A	37%	-0.007	1.0E-01	116,320	0.022	8.7E-06	92,396	7.2E-06
rs972303	yes	yes	*CDH10*	5	24391312	T/C	75%	-0.008	1.6E-01	87,302	0.032	1.9E-06	64,371	4.1E-06
rs4898764	yes	yes	*GNPNAT1*	14	52334821	G/A	53%	-0.013	2.6E-03	114,264	0.016	8.5E-04	90,762	4.2E-06
rs17470444	yes	yes	*SGCZ*	8	14852373	A/G	71%	-0.014	1.4E-02	86,472	0.029	1.0E-05	61,247	4.0E-07
rs2069664	yes	yes	*IQGAP2*	5	75952190	G/A	53%	-0.015	3.3E-03	88,448	0.019	9.1E-04	65,083	5.7E-06
rs741361	yes	yes	*SLC2A3*	12	7966952	A/G	60%	-0.016	1.7E-03	89,766	0.022	2.7E-04	64,771	8.6E-07
rs2673140	yes	yes	*IRS1*	2	226868111	G/A	38%	-0.017	2.0E-04	114,393	0.018	4.1E-04	92,271	1.7E-07
rs2042995	yes	yes	*TTN*	2	179266611	C/T	23%	-0.018	2.3E-03	66,222	0.022	1.4E-03	91,408	6.2E-06
rs10881574	yes	yes	*RXRA*	9	136345043	C/T	7%	-0.032	2.2E-03	88,999	0.045	4.0E-04	62,482	1.6E-06
rs7042428	yes	yes	*PTPRD*	9	8252414	A/G	98%	-0.053	4.3E-03	99,878	0.073	2.9E-04	79,410	2.5E-06
rs17809093	yes	yes	*CECR2*	22	16370258	G/C	4%	-0.062	8.7E-04	67,968	0.071	6.5E-04	47,894	1.1E-06

Chr: Chromosome; Pos: position; EAF: Effect Allele Frequency; EA: Effect allele; OA: Other allele

^a^ ‘Yes’ if the locus is mentioned as WHR_adjBMI_ locus for the first time

^b^ ‘Yes’ if the sex-difference in the effect on WHR_adjBMI_ is reported for the first time

^c^ Effect allele is according to the WHR_adjBMI_ increasing allele according to the associated sex.

### No evidence for loci with simultaneous age- and sex-specific effects

We searched for loci with sex-specific effects on WHR_adjBMI_ that differ between the two age-groups and for loci with age-specific effect on BMI that differ between men and women by testing a three-way interaction (SNP x AGE x SEX, *P*_*agesexdiff*_). We first tested for this three-way interaction in the 59 SNPs identified with an age-difference (15 loci for BMI) or a sex-difference (44 loci for WHR_adjBMI_), as described above. However, none of these 59 loci showed a significant three-way interaction (*P*_*agesexdiff*_ > 0.00084 = 0.05/59, Bonferroni corrected) (**S4 and S5 Tables**). When screening for the three-way interaction genome-wide, no such loci were identified (at 5% FDR) (**Fig 1**).

### Detecting loci with age- or/and sex-interaction requires extremely large sample sizes

**Fig 4:**
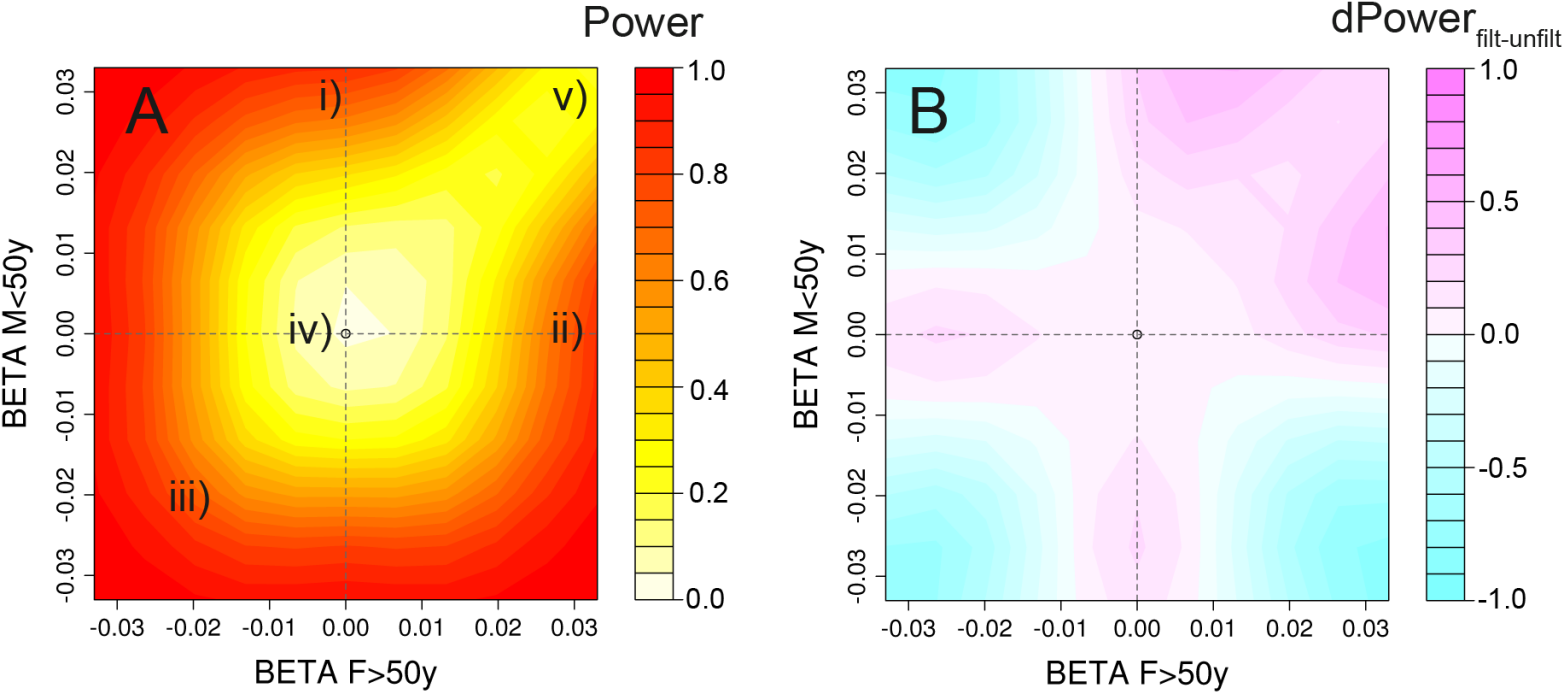
Power heatplots. Power for the combination of screens and gain through a priori filtering for varying configurations of effect sizes across the 4 strata. The figures illustrate (A) the power to detect age-difference, sex-difference or age-sex-difference in at least one of our scans (on *P*_*agediff*_, *P*_*sexdiff*_ and *P*_*agesexdiff*_, with and without a priori filtering); and (B) a power comparison, comparing approaches with and without a priori filtering on *P*_*Overall*_ < 1x10^-5^. We here assume four equally sized strata and a total sample size of N = 300,000 (comparable to the sample size in our BMI analyses). We set b_F≤50y_ = 0.033 (corresponding to a known and mean BMI effect in *MAP2K5* region with R^2^ = 0.037%), b_M>50y_ = 0, and vary b_F>50y_ and b_M≤50_ on the axes. This strategy allows us to cover the most interesting and plausible interaction effects: Two-way interactions, such as (i) pure age-difference (b_≤50y_ = 0.033, b_>50y_ = 0) and (ii) pure sex-difference (b_F_ = 0.033, b_M_ = 0); and three-way interactions, such as (iii) extreme three-way interaction with opposite direction across AGE and SEX, (iv) 1-strata interaction (b_F≤50y_ = 0.033, b_F>50y_ = b_M≤50y_ = b_M>50y_ = 0), and (v) 3-strata interaction (b_F≤50y_ = b_F>50y_ = b_M≤50y_ = 0.033, b_M>50y_ = 0).

We analytically computed the statistical power of our screens to identify SNP x AGE, SNP x SEX or SNP x AGE x SEX interaction effects, assuming a total sample size of 300,000 individuals distributed across four equally sized strata and considering a range of effect size configurations informed by previous observations (**S9, S10** and **S11 Figs**). For example, for a medium genetic effect on BMI (R^2^ = 0.037% as observed previously for a locus near *MAP2K5 [28]*), our screens had (i) sufficient power to identify genetic loci with two-way SNP x AGE or SNP x SEX interactions (i.e. loci with effect in one stratum and not in the other, so-called *pure two-way interaction*, power = 86%, or loci with effect in both strata, but with opposite effect direction, power = 99%), (ii) sufficient power to detect *extreme three-way interaction* SNP x AGE x SEX, typically involving a biologically-unlikely scenario with opposite effect directions across both AGE and SEX (power = 99%), but (iii) insufficient power to identify loci with biologically more plausible three-way interactions (in the range of R^2^ of 0.01–0.05%), i.e., loci that have an effect in only one stratum and not in the other three strata, *1-stratum interaction*, power = 2%, or those with a similar effect in three strata and not in the fourth, *3-strata interaction*, power = 21% (**Fig 4**). Identification of loci with medium 1-stratum (R^2^ = 0.037% in one stratum and R^2^ = 0 in the other three strata) or 3-strata (R^2^ = 0.037% in three strata and R^2^ = 0 in one stratum) interaction effects with a power of 80%, would require a total sample size of 750,000 or 600,000 individuals, respectively.

Reducing the multiple testing burden by applying a filter on the overall meta-analysis to first identify SNPs with main effects (*P*_*Overall*_ < 10^−5^) improved the statistical power to identify loci with specific interaction scenarios: (i) loci with pure two-way interaction effects (e.g. 30% power increase to detect SNP x AGE with R^2^ = 0.037% and R^2^ = 0 in the two strata), or (ii) loci with 3-strata interaction effects (e.g. 21% power increase for loci with R^2^ = 0.037% in three strata and R^2^ = 0 in one stratum) (**Figs 4** and **S9**).

With our sample size of 300,000 subjects and equally sized strata we had 80% power to detect (i) 1-stratum interaction with R² = 0.09% in one stratum (R² = 0 in the other three strata), (ii) 3-strata interaction with R² = 0.07% in three strata (R² = 0 in one stratum), or (iii) pure two-way interaction with R² = 0.03% in one stratum (R² = 0% in the other stratum).

In summary, this analysis suggests that our study is sufficiently powered to detect even subtle two-way interaction effects, and would certainly include effect-sizes that would be considered biologically or clinically important. While even more subtle interactions may be occurring, it appears likely that in this effort, we have detected the most important age- and sex- interactions for body size and shape.

### Association of identified loci with other traits

To examine whether the age- and sex-specific effects of the identified BMI and WHR_adjBMI_ loci translate into similar age- and sex-effects on obesity-related cardiometabolic traits, we gathered results from the ICBP, CHARGE and Global-BPGen consortia (age-specific and sex-specific effects in blood pressure) [30], Global Lipids Genetics Consortium (GLGC) (sex-specific effects in lipids) [31], DIAGRAM (sex-specific effects for type 2 diabetes) [32]and MAGIC (sex-specific effects of glycemic traits, personal communication) [33](S6–S10 **Tables**). Only CHARGE, Global-BPGen and ICBP had previously performed GWAS searching for age-specific effects on blood pressure [34]. None of the 15 age-specific BMI-associated loci influenced blood pressure in an age-specific manner (P_SNPxAGE_ > 0.0033 = 0.05/15) (**S6 Table**). Eight of the 44 sexually dimorphic WHR_adjBMI_ loci show directionally consistent female-specific effects in other traits (**S10 Table**), but none attained significant sex-difference (*P*_*sexdiff*_ > 0.0011 = 0.05/44).

In addition, we performed a systematic search in the National Human Genome Research Institute (NHGRI) GWAS Catalog (www.genome.gov/gwastudies) to examine previously reported GWAS-associations for potential age- or sex-specificity for the loci we identified for BMI and WHR_adjBMI_, respectively [35]. While no associations have been reported that corroborate the sex- or age-specificity of our findings, largely because few sex-stratified and no age-stratified genome-wide studies have been performed to date (this study is among the first ones), many main-effect associations with a wide range of traits and disease have been reported for our age- or sex-specific BMI or WHR_adjBMI_ loci (**S11** and **S12 Tables**). For example, the four loci that showed a larger effect in the older group are known for their association with type 2 diabetes (T2D, near *TCF7L2* and *COBLL1*) or with coronary artery disease (CAD, near *SLC22A3* and *APOC1*). The fact that disease status may correlate both with age and obesity traits may confound our age- or sex-specific findings. To reduce this possibility we repeated the meta-analyses restricted to population-based samples (excluding all case-control studies) and observed similar effect sizes compared to the original meta-analysis (**S13** and **S14 Tables**).

### Age-specific effects of BMI loci extend across the life course

We then examined whether the age-specific effects of the 15 BMI loci extend to younger ages and across the life course by performing look-ups in (i) a GWAS for birth weight [36] and for childhood obesity [37] from the Early Growth Genetics (EGG) Consortium, (ii) a GWAS for BMI of individuals aged 16–25 years [38], and (iii) a GWAS for weight change during adulthood (personal communication).

We found no evidence of association with *birth weight* (N = 26,836) for any of our 15 age-dependent BMI-associated loci (**S15 Table**) [36]. In contrast, we observed nominal significant associations with risk of *childhood obesity* (N = 13,648) for 10 of the 11 variants with stronger effect on BMI in the younger adults (**Tables 3** and **S16**). The four loci that only showed association with BMI in the older adults were not associated with childhood obesity risk (**S16 Table**) [37].

**Table 3:** Enrichment analyses using look-up data for the 15 age-group specific BMI loci. The look-up data is taken from the EGG consortium for birth weight and for childhood obesity, and from personal communication for weight change trajectories. More details including SNP specific effect sizes or odds ratios and association P-Values on the look-up trait can be found in **S15 Table** (for birth weight), **S16 Table** (for childhood obesity) and **S18 Table** (for weight change).

Look-up data set	Sample size	#SNPs tested	#SNPs concordant with the ≤50y vs >50y association pattern	*P*_*binomial*_ ^a^	Loci with expected association pattern
Birth weight	26,836	11	0^b^	>0.99	-
Childhood obesity	13,648	11	10^b^	1.0 x 10^−15^	*FTO*, *TMEM18*, *MC4R*, *ADCY3*, *NEGR1*, *TNNI3K*, *SEC16B*, *CBLN4*, *DDC*, *STK33*
16–25y age-group	29,880	11	9^b^	2.0 x 10^−13^	*FTO*, *TMEM18*, *MC4R*, *ADCY3*, *NEGR1*, *TNNI3K*, *SEC16B*, *CBLN4*, *Intergeneic*
Weight change	39,041	15	5^c^	2.4 x 10^−5^	*FTO*, *STK33*, *TCF7L2*, *SLC22A3*, *APOC1*

^a^ One-sided binomial P-values that test for enrichment of nominal significant and directionally consistent association in the look-up data.

^b^ For the BMI increasing alleles of the 11 SNPs with stronger effect on BMI in ≤50y, we expect to see a nominal significant association with increased birth weight, increased risk for childhood obesity and increased BMI in the 16–25y age-group.

^c^ For the BMI increasing alleles of the 11 SNPs with stronger effect on BMI in ≤50y, we expect to see a nominal significant association with negative effect on weight change (weight loss), and for the BMI increasing alleles of the four SNPs with stronger effect on BMI in >50y, we expect to see a nominal significant association with positive effect on weight change (weight gain) (see Methods for details).

Furthermore, nine of the 11 variants with stronger effect on BMI in the younger adults (18-50y) showed directionally consistent association with increased BMI in the youngest *16–25y age-group* (N = 29,880, **Tables 3** and **S17**). A more detailed experimental examination of effect sizes across the three age-groups did not reveal significant trends (**S12 Fig, S17 Table,** and **S1 Text**).

Finally, we speculated that a higher genetic BMI effect in the younger adults would translate into weight loss and a higher genetic BMI effect in the older adults would translate into weight gain with increasing age (**Methods**). Five of the 15 loci with age-specific effects on BMI showed a nominal significant association accompanied by the hypothesized direction on *weight change* (N = 39,041, **Tables 3** and **S18**).

In summary, the age-dependency of the 15 loci is supported by directionally consistent enrichment of nominal significant associations (*P* < 0.05) with *childhood obesity*, with BMI in the *16–25y age-group* and with *weight changes* across adulthood (*P*_*Binomial*_ ranging from 2.4 x 10^−5^ to 1.0 x 10^−15^, **Table 3**).

### eQTL analysis

#### eQTLs in humans

We performed sex-specific *cis* eQTL analyses in lymphoblastoid cell lines of the combined Groningen and EGCUT studies (1,450 men and 910 women) [39, 40] for the 44 SNPs showing sex-specific effects for WHR_adjBMI_ to determine whether there is evidence to support sex-specific regulatory effects of the index variants on adjacent gene expression. Two SNP-gene associations displayed significant differences in genetic effects on expression between men and women (FDR(*P*_*Sexdiff*_) < 5% with and without initial filtering on overall expression effects):rs6088552–*ACSS2* and rs6088735–*MYH7B* (**S19 Table**). While both SNPs were associated with WHR_adjBMI_ in men-only (and no effect in women), the first SNP showed no effect on gene expression in men but was associated with gene expression in women, and the second SNP rs6088735 was associated with gene expression in both sexes, but higher in men and lower in women. The two loci were located at only 519kb from each other (rs6088552 near *PIGU*, rs6088735 near *EDEM2*, at chr20:33-34Mb, r^2^ = 0.07), each showing independent sex-specific associations with WHR_adjBMI_ and each also showing independent sex-specific association with the expression of two different genes (*ACSS2* and *MYH7B*, respectively) (**S13 Fig).** *ACSS2* (acyl-CoA synthetase short-chain family member 2) is a cytosolic enzyme, transcribed by SREB-proteins, that catalyzes the production of acetyl-CoA for use in both lipid synthesis and energy generation acids [41]. *MYH7B* (myosin, heavy chain 7B, cardiac muscle, beta) encodes a heavy chain subunit for slow-twitch myosin, largely expressed in heart and skeletal muscle tissue, and is involved in ATP-hydrolysis.

Age-stratified analysis were not performed for EGCUT as the study participants were relatively young (mean age: 37y), with too few individuals in the >50y age-group. Instead, we examined association between the 15 age-specific loci and gene expression using data from 3,489 unrelated individuals (N = 2,531 for <50y, N = 958 for ≥50y) from the NESDA and NTR cohorts [42, 43]. No SNP showed a significant age-specific effect on gene expression (FDR(*P*_*agediff*_ *)* > 5% for all SNP-gene expression combinations).

#### eQTLs in mice

We compared expression of genes harboured by the identified loci in inguinal and gonadal fat in age-matched male, female or ovariectomized female (OVX) C57/BL6 mice maintained on a high-fat (HF) diet [44].

For genes located in the 15 age-specific BMI-associated loci, we compared expression in OVX female mice with the expression in the other male and female mice, but no differences in gene expression were observed.

For genes located in the 44 sex-specific WHR_adjBMI_-associated loci, we compared expression in female mice (OVX and non-OVX) with the expression in male mice. The expression of two genes reached significance (*P* < 6.4x10^-4^ = 0.05/(39 x 2)), corrected for testing 39 genes with homologous regions, and two tissues). The expression of *IQGAP2*, which regulates cell adhesion and motility, (rs2069664) was higher (*P* = 2.3x10^-7^) in gonadal fat tissue of male compared to female mice, whereas the expression of *TP53INP2*, a co-factor for the thyroid hormone receptor, (rs6088552) was higher (*P* = 2.3x10^-6^) in inguinal fat tissue of male compared to female mice. *TP53INP2* is located in the same chromosomal region for which we found evidence for sex-specific associations with the expression of *ACSS2* and *MYH7B* in humans. Interestingly, Tp53inp2 has also been named the DOR (Diabetes and Obesity Related) gene, as its expression is substantially reduced in skeletal muscle of obese diabetic fa/fa Zucker rats [45]. Muscle-specific overexpression of Tp53inp2 in mice leads to reduced muscle mass, whereas a deletion leads to muscle hypertrophy [46]. *TP53INP2* expression was markedly reduced in muscle from individuals with type 2 diabetes and in rodent diabetes models [46].

### Pathway analyses

We applied pathway analyses to gain insight into mechanisms that might be involved in the age- and sex-specific difference in body size and body shape. We assumed that loci even with moderate evidence for age- or sex-difference for BMI and WHR_adjBMI_, respectively, are enriched for genes that contribute to the age-specific BMI association or sex-specific WHR_adjBMI_ association (**Methods**). We used the DEPICT software to perform gene set enrichment and gene expression analyses [47] (**S20** and **S21 Tables** and **S1 Text**), and QIAGEN’s Ingenuity Pathway Analysis (IPA, QIAGEN Redwood City, www.qiagen.com/ingenuity) tool for pathway analysis and functional annotation (**S22–S26 Tables** and **S1 Text**). Both the DEPICT and the IPA analyses identify the possible influence of sex-specific WHR_adjBMI_ loci in androgen biosynthesis, a hormone known to decrease the storage of lipids in adipose tissue [48]. Additionally, PPARα/RXRα activation, the most significant canonical pathway for loci with a greater effect on WHR_adjBMI_ in women, may be inhibited in the presence of estrogen, thus decreasing the breakdown of lipids through competitive receptor binding [49]. To fully understand the possible age- and sex- specific regulatory effects these identified genes may have in the identified pathways, gene sets, and biological functions, further analyses are needed.

### Heritability and explained variance analyses

To assess whether the age-group differences observed for BMI and the sex-differences observed for WHR_adjBMI_ extend to the contribution of all 2.5M variants (narrow-sense heritability), we calculated heritability using the GCTA method [50] in several large studies (N = up to 29,232 individuals) for all, for women and men, for the younger and older adult groups. The variance explained by the 2.5M variants was 21% for BMI and 10% for WHR_adjBMI_, with no significant difference between age groups for BMI (*P*_*agediff*_ = 0.19) or between men and women for WHR_adjBMI_ (*P*_*sexdiff*_ = 0.48) (**S27 Table**).

To further investigate differences between subgroups, we calculated the variance explained in the discovery data set for subsets of SNPs based on varying thresholds of overall association on BMI or WHR_adjBMI_ (**S14 Fig**). When we included only SNPs that reached genome-wide significance for BMI (*P*_*Overall*_ < 5x10^-8^), the variance explained in the younger adults (3.4%) was significantly larger than in the older (2.45%) adults. As we increased the significance threshold and included more SNPs with less significant overall association, the difference between the two age groups reduced and became non-significant once SNPs with a *P*_*Overall*_ > 3x10^-5^ were included. We observed similar significant differences in explained variance for WHR_adjBMI_ between men and women, with the most pronounced difference for genome-wide significant SNPs (*P*_*Overall*_ < 5x10^-8^, women 1.60%; men: 0.70%) that reduced and became non-significant for SNPs with a *P*_*Overall*_ > 1x10^-5^. Consistent with the observed interactions, we found no difference in explained variance between men and women for BMI or between the younger and the older group for WHR_adjBMI_ at any *P*_*Overall*_ cut-off (**S14 Fig**).

Family-based heritability estimates, from the Family Heart Study (N = 1,810, 454 families), showed similar (but non-significant) trends for younger versus older adults for BMI (60% vs 45%, *P*_*agediff*_ = 0.24), for women and men for WHR_adjBMI_ (43% vs 38%, *P*_*sexdiff*_ = 0.68) (**S27 Table**).

Collectively, these observations are consistent with the results of our genome-wide search, showing that genetic variants contribute more to BMI variation in younger than in older adults and more to WHR_adjBMI_ variation in women than in men. These differences are most pronounced when we test genome-wide significant SNPs only, while differences are minimized as more SNPs with weaker associations are included.

### Joint testing of main- and interaction effects yield novel loci for BMI and WHR_adjBMI_

Our stratified analysis approach also offered an opportunity for discovery of novel variants influencing BMI and WHR_adjBMI_ by (i) using a joint 4df test of the main SNP effect in the presence of interaction [27] and (ii) by overall meta-analysis of the 4 strata. Both approaches increase statistical power to detect a main effect if there is evidence of heterogeneity across the strata. Of the 164 loci that reached genome-wide significance for BMI (P < 5x10^-8^), 73 are novel (**S28 Table** and **S1, S2** and **S15 Figs**). Of the 73 loci, 45 were only identified in the overall test and 26 were identified in both tests. The remaining two loci were only identified in the joint test and either displayed evidence for difference between men and women (near *CXXC5*, *P*_*sexdiff*_ = 2.7x10^-5^) or between age-groups (near *DDC*, *P*_*agediff*_ = 6.2x10^-4^ *)* suggesting that its identification may have been aided by allowing for interaction. We identified 53 loci with significant associations with WHR_adjBMI_, of which 10 were novel (**S29 Table and S1, S2** and **S16 Figs**). It can be speculated that the yield of novel SNP associations for BMI was greater than that of WHR_adjBMI_, because age-dependent effects have not been sought systematically before, whereas sex-specific screens have been performed previously [10].

## Discussion

Our genome-wide search for age- and sex-specific loci in up to 320,485 adults of European ancestry identified 15 loci that were associated with BMI in an age-dependent manner, with predominantly larger effects in the younger than in the older adults. Notably, despite sufficient statistical power, we did not identify BMI-associated loci with sex-dependent effects. The largest association study on BMI *[19]* identified two SNPs with different impact on BMI in men and women: rs543874 (*SEC16B*) and rs6091540 (*ZFP64*). While these SNPs show more modest trends towards sex-different effect (*P*_*sexdiff*_ = 2.4x10^-4^ and 1.3x10^-4^, respectively) in our study, they were not picked up by our analysis due to the different pre-filtering strategy. In contrast to BMI and consistent with previous observations for WHR_adjBMI_, we identified 44 WHR_adjBMI_ associated loci with sex-specific effects of which the majority have a larger effect in women compared with men. No age-specific WHR_adjBMI_ loci were discovered.

Our work is the first large-scale genome-wide association study to interrogate the influence of both age and sex, simultaneously, on genetic effects for BMI and WHR_adjBMI_. While our meta-analysis had sufficient power to identify SNP-by-age or SNP-by-sex interactions, we only discovered loci influenced by age for BMI. Studies that followed up on previously established BMI loci in longitudinal and cross-sectional designs support our findings regarding the age-dependency of the majority of these loci [38, 51–57]. Indeed, for 11 of the 15 loci identified in our study, the effect on BMI was 1.5 to 3.5 times smaller in the older adults than in the younger adults, which may reflect a greater culmination of environmental and lifestyle factors on adiposity in older adults that overwhelm the genetic effects. While none of these loci were associated with birth weight, all—but one—were nominally associated with increased risk of childhood obesity. Results from a GWAS on BMI in 16-to-25 year-olds [58] provide preliminary evidence that some loci exert their largest effects relatively early in life, whereas others become more pronounced in young adulthood. Notwithstanding the predominance of BMI loci with larger genetic effects in younger individuals we identified four loci with stronger genetic effects in older adults. Interestingly, these four loci have been previously associated with either type 2 diabetes [32] or coronary artery disease [59]. Sensitivity analyses precluded potential ascertainment bias introduced by disease studies in the older group. These loci may influence BMI through mechanisms that are distinct from other BMI-associated loci; mechanisms that may be more closely related to processes more directly involved in the pathogenesis obesity-related diseases. Furthermore, the directional consistent genetic effects of our loci on weight change during adult life from longitudinal studies supports our finding.

Indeed, the stratification into age-groups may introduce a cohort effect that implies a different genetic or environmental make-up of cohorts with older *vs* younger adults. For example, the obesogenic environment that has fueled the obesity epidemic that westernized societies have experienced during the past 30 years may have affected older individuals differently than younger individuals. To examine the contribution of such cohort effects and to obtain more accurate age-dependent effect estimates, large-scale genetic longitudinal studies would be required that measure BMI at multiple time points with individuals born across a wide range of birth years.

While our study provides some first insights into age-dependent genetic effects, in particular before and after menopause, more data from larger studies with longitudinal data spanning from childhood through late adulthood are desirable to accurately assess the influence of these loci on BMI across the life course. Indeed, identifying the time of life when variants affect body weight the most may help us determine the mechanisms of their influence on body weight and potential for intervention.

In contrast to the observations for BMI, our genome-wide interaction analyses did not identify loci with age-dependent effects for WHR_adjBMI_ but there was strong novel evidence for sex-influenced effects in 44 loci. For 27 of the 44 loci, the sexual dimorphism is reported for the first time, with 17 being completely novel associations for WHR_adjBMI_. Due to increased sample size and optimized SNP selection approaches, we more than doubled the number of loci with established sex-difference for WHR_adjBMI_ [10, 18, 29]. The 44 loci divide into 11 loci with opposite effects between men and women, 28 loci with a stronger effect in women and five loci with a stronger effect in men. This is the first report to highlight loci with opposite effects and the enrichment of women-specific WHR_adjBMI_ associations is consistent with previous findings.

We examined whether the sex-dependent effects on WHR_adjBMI_ were mediated through sex-specific effects on the expression of genes located within these loci, using data available from eQTL analyses in humans and mice. Of particular interest is a region at chromosome 20q11.22 in which two independent WHR_adjBMI_ lead SNPs near *PIGU* and near *EDEM2* showed independent sex-specific associations with the expression of *ACSS2* and *MYH7B*, respectively, in humans. While we found no direct evidence of sex-specific action of *ACSS2* or *MYH7B*, based on current knowledge, both proteins seem to be involved in peripheral energy metabolism. In addition, we observed that the expression of Tp53inp2 (Tumor Protein 53 Inducible Nuclear Protein 2), of which the human TP53INP2 ortholog is also located in the *PIGU* locus, had significantly higher expression levels in the inguinal fat of male than female mice. This observation is consistent with a previous study, showing that Tp53inp2 expression in white adipose tissue is significantly higher in male than in female mice [60]. The authors speculated that this sex-specificity might be due to differences is fat distribution with females storing proportionally more fat in subcutaneous/inguinal and males more in intra-abdominal depots [60]. Taken together, the sex-specific association with WHR_adjBMI_ of two independent loci at chr20q11.22 may be mediated through any or all three genes for which we found sex-specific expression. While all three genes are good candidates, experimental follow up will be needed to pinpoint the causal gene(s) and to elucidate the function and sex-specificity.

Our broad-sense (family-based analyses) or narrow-sense (GCTA including all 2.5M variants) heritability estimates showed no difference in explained variance between men or women, or between younger and older adults for either outcome. However, when considering subsets of variants displaying overall significant associations (*P*_*Overall*_ < 1x10^-5^), we observed a significant difference between age- but not sex- groups for BMI, with a larger explained variance among the younger than the older adults, and between sex- but not age groups for WHR_adjBMI_, with a larger explained variance in women than in men. These observations further corroborate the predominance of age-dependent loci for BMI and sex-dependent loci for WHR_adjBMI_ identified through a genome-wide screen.

Even though our study is likely the largest GxE and the first GxE_1_xE_2_ interaction GWAS meta-analysis ever conducted, we did not detect loci with sex-specific effects for BMI (SNP x SEX), age-specific effects for WHR_adjBMI_ (SNP x AGE) or three-way interactions effects (SNP x AGE x SEX). Three-way interactions are biologically plausible when considering that sex-specific effects might be exerted through hormones and that the hormonal status particularly of women changes at menopause (i.e., around the age of 50 years). This would result in a 1-stratum interaction (i.e., genetic effect only present in younger women) or a 3-strata interaction (i.e., genetic effect present in all but in younger women). While our study had sufficient power (power > 80%) to identify any kind of two-way interaction (SNP x SEX or SNP x AGE) even for effects as small as those observed for established BMI or WHR_adjBMI_ loci, our power was limited specifically for the biologically plausible three-way effects (1-stratum or 3-strata-interaction). To detect subtle effects appearing in only one of the four strata will require specialized study designs or alternative approaches. We provide a detailed analytical perspective on the power to detect different interaction signals that may inform other studies aiming at detecting interaction effects.

We acknowledge that our power estimations are expressed as a function of previously observed explained variances, incorporating measurement error. As measurement error increases, the variance of the phenotype increases and—because the genetic effect is not affected—the explained variance of the genetic variants decreases. While a random measurement error in the dependent variable of a linear regression model would not lead to a biased effect size estimate, such an error would increase the standard errors of the effect size estimates compared to a measurement error free outcome. Under the alternative hypothesis, this results in smaller statistical power. This would imply, for our analysis, that we have potentially missed some true associations, which could have been detected with smaller measurement error.

With the growing sample-size and thus statistical power, measurement error is often larger than the variant-wise effect size estimate for many human traits currently under investigation in large-scale GWAS. Thus, an individual variant’s effect may not have clinical significance by itself in predictive models. However, its ultimate significance should be evaluated in the context of the biological mechanism it reveals along with other discovered variants, and the potential of such a mechanism as a therapeutic target; this is yet to be determined. In order to discover more disease-associated genetic variants, reducing measurement error by repeated and/or more accurate measurements is a viable alternative to only increasing sample size–especially when the measurement error relative to the outcome variability is high.

For technical reasons, variants on the X-chromosome were not screened. Yet, an interesting hypothesis is that sex-linked variants contribute to a sex-dependent architecture of body size and shape, both of which exhibit obvious sexual dimorphism. These analytic challenges are being addressed currently, and exploration of X-linked variation is warranted. Further, we have included only individuals of European-ancestry and thus cannot report on the generalizability of our findings to other race or ethnic groups. While we examined age-dependent effects by binning individuals below and above age 50 years—an average age of menopause—it is possible that modeling of age as a continuous trait might have had superior power. This approach poses more complex harmonization issues that should be addressed in a follow-up study. In addition, we recognize that environmental modifiers may further influence the effect of trait-related loci, and that some of the interactions we identified may be proxies for interactions with other environmental factors that are correlated with either age or sex.

In summary, our findings further distinguish the genetics of BMI from the genetics of WHR_adjBMI_. Previously described aspects of distinction include the enrichment of neural pathways versus insulin-related pathways and sexual consistency versus sexual dimorphism, respectively [61, 62]. Our findings suggest that genetic BMI effects can change by age possibly depicting different mechanisms of genetic BMI effects that either increase or decrease during adult age. The knowledge of such mechanisms might guide the development of more effective intervention programs that are desperately sought after.

## Methods

### Anthropometric phenotypes

The anthropometric traits examined are body mass index (BMI, kg/m²), which is a measure of body mass and a surrogate for total body fat, and waist-to-hip-ratio adjusted for BMI (WHR_adjBMI_), which is a measure of body fat distribution. Traits were transformed before analyses; we first created age- (and BMI) adjusted residuals (including age and age² into the regression for BMI, and additionally BMI for WHR_adjBMI_) for each of the four strata separately (men ≤50y, men >50y, women ≤50y, and women >50y) and subsequently applied an inverse normal transformation.

### Study-specific analyses

We included up to 92 studies (totalling up to 21,989 men ≤50y, 74,324 men >50y, 41,386 women ≤50y, and 88,625 women >50y) with genome-wide genotyping chip data using either Affymetrix or Illumina arrays. To enable meta-analyses across different SNP panels, each study group performed genotype imputation using HapMap II CEU (build 21 or 22) via MACH [63], IMPUTE [64] or BimBam [65] yielding ~2.8 Million SNPs. In addition, we included 22 studies (up to 28,106, 18,877, 29,306, 17,872 individuals for each of the strata, respectively) for BMI and WHR_adjBMI_ that were genotyped using the custom iSELECT Metabochip array containing ~195K SNPs designed to support large-scale follow-up of putative associations with metabolic and cardiovascular traits [66].

In each study, SNP associations were tested separately by age-group and sex (men ≤50y, men >50y, women ≤50y and women >50y) for autosomal variants. The additive genetic effect for each SNP on each phenotype was estimated via linear regression using MACH2QTL [67], SNPTEST [64], ProbABEL [68], GenABEL [69], Merlin [70], PLINK [71] or QUICKTEST [72]. For studies with a case-control design, cases and controls were analysed separately. See **S1, S2** and **S3 Tables** for study specific genotyping, imputation, analysis, quality control and phenotypic descriptive information. In total we gathered association data from up to 92 studies with imputed GWAS data and 22 studies genotyped on the Metabochip array for BMI including up to 320,485 individuals and 64 studies with imputed GWAS data and 20 studies genotyped on the Metabochip array for WHR_adjBMI_ including up to 216,654 individuals.

All studies were conducted according to the principles expressed in the Declaration of Helsinki. The studies were approved by the local Review Boards and all study participants provided written informed consent for the collection of samples and subsequent analysis.

### Quality control of study-specific aggregated data

All study-specific files were processed in the meta-analysis centers through a standardized quality-control (QC) pipeline [73]. This involved QC checks on file completeness, range of test statistics, allele frequencies, trait transformation and population stratification as well as filtering on low quality data. Briefly, we excluded monomorphic SNPs, SNPs with MAF*N ≤ 3 (minor allele frequency multiplied by sample size), imputed SNPs with poor imputation quality: r2_hat < 0.3 in MACH, observed/expected dosage variance < 0.3 in BIMBAM, proper_info < 0.4 in IMPUTE, information < 0.8 in PLINK [64, 65, 67, 71]; genotyped SNPs with low call-rate (< 95%), and genotyped SNPs that were out of Hardy-Weinberg equilibrium (HWE, P-Value testing for HWE < 10^−5^). To increase the overlap in the number of SNPs between imputed GWAS and MetaboChip data, we transferred all SNP identifiers to unique SNP names consisting of chromosomal and base position, e.g. using chr1:217820132 instead of rs2820443 in the meta-analysis. Sex- and age-specific standard errors and P-values from each participating study were genomic-control (GC) corrected using study- and strata-specific lambda factors [74], whereas the lambdas were estimated from all genome-wide available SNPs for imputed GWAS and form a subset of 4,427 QT-interval SNPs for MetaboChip studies.

### The meta-analyses

Generally, beta-estimates and standard errors were meta-analyzed using an inverse-variance weighted fixed effect model as implemented in METAL [75].

We meta-analyzed effect estimates and standard errors from all available studies in each of the four strata separately, yielding *b*_*M*≤50*y*_, *b*_*M*>50*y*_, *b*_*F*≤50*y*_, *b*_*F*>50*y*_ and *SE*_*M*≤50*y*_, *SE*_*M*>50*y*_, *SE*_*F*≤50*y*_, *SE*_*F*>50*y*_. By meta-analyzing *b*_*M*≤50*y*_ and *b*_*M*>50*y*_ we obtained the effect and standard error for men (*b*_*M*_,*SE*_*M*_) and women (*b*_*F*_,*SE*_*F*_). Similar meta-analyses yielded the age group-specific association statistics, *b*_≤50*y*_ and *b*_>50*y*_ with standard errors *SE*_≤50*y*_ and *SE*_>50*y*_. Meta-analysis of all four strata provided the overall association effect estimate *b*_*overall*_, standard error *SE*_*overall*_, and P-value *P*_*overall*_. A joint meta-analysis based on the pooled stratum-specific estimates was performed according to Aschard et al [27].

After the meta-analyses, we performed an additional quality control step on the meta-analytic results: We only included SNPs (i) being available in at least half of the maximum sample size in all strata; and (ii) having chromosome and position annotation in dbSNP.

### Genome-wide screening approaches to detect interaction effects

Our study aimed at discovering SNPs with (1) ***age-different*** effects, (2) ***sex-different*** effects, and (3) ***age-dependent sex-different*** effects or ***sex-dependent age-different*** effects.

To find *age-different* genetic effects, we computed age-difference P-values (*P*_*agediff*_) by testing for difference between the age group-specific meta-analyzed beta-estimates *b*_≤50*y*_ and *b*_>50*y*_ using

$$t_{age}=\frac{b_{\leq 50y}-b_{>50y}}{\sqrt{{SE}_{\leq 50y}^{2}+{SE}_{>50y}^{2}-2r_{age}∙{SE}_{\leq 50y}∙{SE}_{>50y}}}.$$

The correlation *r*_*age*_ between *b*_≤50*y*_ and *b*_>50*y*_ computed as the Spearman rank correlation coefficient across all SNPs for BMI and WHR_adjBMI_ was 0.123 and 0.049, respectively. The analogous test statistic for *sex-different* effects was

$$t_{sex}=\frac{b_{M}-b_{F}}{\sqrt{{SE}_{M}^{2}+{SE}_{F}^{2}-2r_{sex}∙{SE}_{M}∙{SE}_{F}}},$$

with corresponding P-value (*P*_*sexdiff*_). The Spearman correlation *r*_*sex*_ was 0.121 or 0.047 for BMI and WHR_adjBMI_, respectively.

To test for the three-way interaction of age- and sex-differences, we introduced for the first time a test of difference between age groups in the sex-difference, which is mathematical equivalent to a test of difference between sexes in the age group-difference using the age-sex-difference statistic as

$$t_{agesex}=\frac{\left(b_{M\leq 50y}-b_{F\leq 50y})-(b_{M>50y}-b_{F>50y}\right)}{\sqrt{{SE}_{M\leq 50y}^{2}+{SE}_{F\leq 50y}^{2}+{SE}_{M>50y}^{2}+{SE}_{F>50y}^{2}}},$$

with the corresponding P-value (*P*_*agesexdiff*_).

To maximize statistical power we did not split our samples (artificially) into discovery and replication sets, but meta-analyzed all studies together and verified the absence of cross-study heterogeneity. We screened genome-wide for *P*_*agediff*_, *P*_*sexdiff*_, and *P*_*agesexdiff*_ for each of the two traits (BMI, WHR_adjBMI_). These screens have ideal power to detect effects that are of opposite direction across the four strata (**S9 Fig**). However, searching for effects that are prominent in one or some strata, but not existent or directionally consistent and less pronounced in other strata profits from an a priori filter on the overall association (*P*_*overall*_ < 10^−5^) as shown previously [10, 26] (**Fig 4**). The rationale behind this filter is that SNPs with unequal effects in the different strata have non-zero overall effect when tested in all strata combined. This is true unless these effects are the same magnitude, but in opposite direction (i.e. cancel out in the combined analysis). Hence filtering on overall association P-value possibly enriches our selection with SNPs showing interaction effects. For BMI and WHR_adjBMI_ 7,382 and 2,014 SNPs passed this filter.

For each trait and for each of the 6 approaches (*P*_*agediff*_, *P*_*sexdiff*_, *P*_*agesexdiff*_; with and without a priori filtering), we controlled the False Discovery Rate (FDR) at 5% to account for the multiple testing [76]. Importantly, controlling the FDR of each single analysis at 5% implies a global FDR control at 5% for the ensemble of discoveries resulting from all the different approaches together.

### Sensitivity analyses using population-based studies only

To ensure the association of none of our age- or sex-specific loci were driven by ascertainment bias through inclusion of case-series of individuals with type 2 diabetes or coronary artery disease, we performed additional meta-analyses restricted to population-based (i.e. no ascertainment bias) studies and compared the effect-sizes between the original meta-analyses and the meta-analyses restricted to population-based studies.

### Sensitivity analyses excluding studies with self-reported BMI or WHR

Self-reported BMI or WHR may cause systematic measurement error that might lead to biased effect estimates. Few of our studies assessed BMI and WHR by self-report in the sense that they told study participants how to measure BMI and WHR for themselves. In order to ensure that the age- or sex-differences of our identified loci was not driven by the few studies that used self-report data (13 of our 114 studies), which may introduce bias [77–79], we conducted sensitivity meta-analyses limited to studies that measured anthropometric phenotypes (**S5** and **S8 Figs**).

### Power computations

To illustrate the strength and characteristics of the various screens outlined, we analytically computed power by scan (**S9 Fig**) and for all scans combined (**Figs 4, S10** and **S11**), for varying configurations of effect size combinations and directions across the four strata. More specifically, we assumed equally sized strata, a total sample size approximately corresponding to the maximum sample size of our study and modelled three categories of SNPs explaining realistic fractions of the phenotypic variance, i.e. small, medium and large effects from Speliotes et al [28] and from Heid et al [29]. The power shown in any of the heatplots was calculated based on a fixed effect in women ≤50y (set to the known effect), a fixed effect in men >50y (set to 0), and varying effects in women >50y and men ≤50y (varying from negative to positive magnitude of the known effect). This strategy allowed us to depict power for most important interaction effects (i.e. for pure sex-difference, pure age-difference, 1-strata interaction and 3-strata interaction) in a single heatplot (see legend of **Fig 4**).

### Genome-wide screening approaches to detect main effects accounting for interaction

To identify novel genetic association for BMI and WHR_adjBMI_, we screened (i) the *P*_*Overall*_ gathered from a four-way meta-analysis of the stratified results and (ii) the *P*_*Joint*_ gathered from a four-way joint meta-analysis of the stratified results according to Aschard et al [27]. We used a genome-wide significance level (P<5x10^-8^) for both approaches to correct for the multiple testing and compared the detected regions to previously established loci using a 500kb distance criterion.

### Establishing enrichment for sex-specific or age-dependent genetic effects

For WHR_adjBMI_, we counted among the sex-different associations (disregarding the opposite effect loci) how many were significantly stronger in men or women. To test whether the observed counts represent significant imbalances between sexes we compared them to the expected binomial distribution (with p = 0.5). Similar exercise was done for age-specific associations for BMI.

### Lookup of age- and sex-specific associations with other phenotypes

Age-group specific association results of the identified loci were requested for blood-pressure measures (diastolic and systolic blood pressure, mean arterial pressure and pulse pressure) from the Global-BPGen consortium [30]. The provided effect size and standard error estimates for six age bins (20–29, 30–29, …, 70–79 years) were combined to derive SNP x AGE interaction effect sizes and P-Values (**S6 Table**) using meta-regression [34].

Sex-specific associations of the identified loci were requested for lipid traits (HDL-C, LDL-C, Total Cholesterol and Triglycerides) from the Global Lipids Genetics Consortium [31], for type 2 diabetes (T2D) from the DIAGRAM consortium[32], for glycemic traits (fasting insulin, fasting glucose, HOMA-B, HOMA-IR) from the Meta-Analyses of Glucose and Insulin-related traits (MAGIC) Consortium[33](personal communication), and for blood-pressure measures (diastolic and systolic blood pressure) from the Global-BPGen consortium [30] (**S7, S8** and **S9 Tables**). The provided men- and women-specific estimates were used to derive sex-difference P-Values.

### NHGRI GWAS catalog lookups

To further investigate the identified genetic variants in this study and to gain additional insight into their functionality and possible pleiotropic effects, we searched for previous SNP-trait associations nearby our lead SNPs. PLINK was used to find all SNPs within 500 kb of any of our lead SNPs using 1000 Genomes Project Pilot I genotype data from the CEPH (Utah residents with ancestry from northern and western Europe) population (CEU) [80, 81]. To identify previous associations, all SNPs within the specified regions were compared with the NHGRI (National Human Genome Research Institute) catalog for overlap and distances between the two SNPs were obtained using SAS, Version 9.2 [citation info below for SAS and PLINK] [82]. The NHGRI’s (National Human Genome Research Institute) GWAS catalog contains only the top 30 most significant SNP-trait associations from recent GWAS published results from studies with at least 100,000 SNPs with resulting P-values of less than P<1x10^-5^ [82]. For previous GWAS results not reported in the Catalog when accessed on 10/15/2014, additional SNP-trait associations were pulled from the literature and compared to our lead SNPs using the same PLINK output file to obtain distance and r^2^ values [83–91]. All previous associations within 500 kb and with an r^2^>0.1 with our lead SNP that reached genome-wide significance in the previous publication were retained for further interrogation.

### Association of age-specific BMI loci with birth weight and childhood obesity

Summary statistics from a genome-wide association meta-analyses previously performed by EGG Consortium (www.egg-consortium.org) were used to examine whether the 15 age-specific BMI loci associate with birth weight and/or childhood obesity risk. Birth weight (BW) had been transformed to z-scores. Association between each SNP and the birth weight was tested using linear regression assuming an additive genetic model, with sex and, where available, gestational age as covariables [36]. In the genome-wide association meta-analysis for childhood obesity risk, cases were defined as having an age- and sex-specific BMI > 95th percentile, and controls as having an age- and sex-specific BMI < 50th percentile in children of European ancestry. SNP associations were assessed in a case-control design assuming an additive genetic model [37].

### Comparison of effect sizes for age-dependent BMI loci with younger individuals aged 16–25 years

We compared the effect sizes for 15 loci with age related differences in BMI for each of the age strata (≤50y and >50y) in men and women combined with the BMI in young adults ages 16–25 years [58]. Nine out of the 14 studies included in the young adult analysis had overlapping samples with the current sample, although the BMI measurements utilized were different (i.e. adolescence/early adulthood versus middle-aged to older adulthood). We used t-tests to compare effect estimates (β) from the younger adults aged 16–25 years (A) to each of our age strata (≤50y or >50y) (B) adjusting for the correlation due to overlapping samples such that:

$$t_{diff}=\frac{b_{A}-b_{B}}{\sqrt{{SE}_{A}^{2}+{SE}_{B}^{2}-2r∙{SE}_{A}∙{SE}_{B}}},$$

where SE = standard error and r = Spearman correlation coefficient between the effect estimates genome-wide. We calculated the Spearman correlation *r* between our study and GIANT using the combined stages from both studies. The significance level (P-value) was based on a two-tailed t-test.

### Look-up of age-dependent BMI loci for weight change across adulthood

We also evaluated the 15 BMI loci showing age-dependent results from genome-wide analyses with weight change across adulthood. Using growth curves generated from multiple measures of weight in individuals between the ages of 20 and 65 years, weight change trajectories were calculated by sex using age as both a random and fixed effect. For each of the 15 loci showing age-differences in BMI, we observationally compared the direction of the effect estimate in the weight change results with the direction of effect seen between our adults aged 18–50 years and adults >50 years. While assuming constant height across adulthood and no cohort effect between the two age-groups, we hypothesized that for loci where we find a stronger effect for BMI in the adults ages 18–50 years compared to adults >50 years, the direction of effect estimate in the weight change data would be negative. For the loci where we found a stronger effect for BMI in the adults >50 years compared to the adults ages 18–50 years we hypothesized that the direction of effect estimate in the weight change data would be positive.

### Expression QTL analyses in human tissue

We examined transcript expression of genes nearby (+/- 1Mb) the 44 identified WHR_adjBMI_ SNP in lymphoblastoid human cell lines available in 2,360 human samples from the EGCUT and Groningen cohorts (910 women and 1,450 men) [39, 40]. We computed sex-specific associations between each of the 44 variants and all genes in their 1Mb vicinity and tested the men- and women-specific eQTLs for sex-difference (*FDR*_*sexdiff*_<5% calculated with/without initial filter on overall expression effect *FDR*_*overall*_<20%).

We next examined whether the 15 SNPs identified to be age-dependently associated with BMI impact nearby (+/- 1Mb) transcripts differently in younger (<50y) than in older individuals (≥50y). As such, we analyzed human whole blood transcription in 3,489 unrelated individuals from NESDA and NTR cohorts [42, 43], which were divided in a ≥50y group (N = 958) and a <50y group (N = 2,531). Cis-eQTL analysis for the 15 SNPs was conducted for the two groups separately and age-group specific eQTLs were compared for age-difference (*FDR*_*agediff*_<5% calculated with/without initial filter on overall expression effect *FDR*_*overall*_<20%).

### Expression QTL analyses of adipose tissues in high-fat-diet-induced obese mice

We performed a microarray analysis on data from an experiment previously published [44]. Briefly, 21 male, 21 female, and 21 ovariectomized (OVX) female C57/BL6 mice were fed from day 21 for 12 weeks on an high fat diet (45% calories from fat; Research Diets, Inc., New Brunswick, NJ). All mice (male, female, and OVX) were exposed to sham or OVX surgery. Animals were sacrificed and tissues collected during the first 2h of the beginning of the light cycle after a 12h fast.

GeneChip microarray (Affymetrix, Santa Clara, CA) was performed according to manufacturer’s instructions on 7 independent pooled samples (3 mice per pooled sample) per experimental group (male, female, OVX) from gonadal adipose tissue (GWAT) and inguinal adipose tissue (IWAT) fat pads. AMC Project Report Version 12 (6/27/07) GeneChip Operating System parameters α1 and α2 were set to 0.05 and 0.065, respectively. Normalized expression values from the Affymetrix identifier were analyzed with the online software server Genesifter (VizX Labs, Inc., Seattle, WA, USA). For comparisons of microarray data sets, multiple t-tests were used to identify genes with at least a twofold difference in gene expression (with Benjamini and Hochberg correction; P<0.05) and at least an expression level of 100. Genes populated from the GWAS studies were compared to this list of genes that met the minimum criteria of expression, fold difference, and p-value. Those identified as being statistically significant were further validated by qPCR.

### Pathway analyses

#### DEPICT

We used a recently developed pathway enrichment method, DEPICT [47]. The methodology first selects all lead SNPs below a certain threshold with respect to a target P-value (available genome-wide). We tested multiple hypotheses corresponding to different lead SNP selection scenarios. First, we selected SNPs with *P*_*sexdiff*_ <0.001. Second, focusing on SNPs with concordant effect size direction (CED), but different magnitude we added a marginal filter to boost power by selecting SNPs with *P*_*sexdiff*_ <0.01 and *P*_*overall*_ <0.01. In case of CED, SNPs with stronger effect in women may fall into separate pathways from SNPs with stronger effect in men. Hence, we have derived gender-specific sex-difference P-values (P_sexdiff_F_, P_sexdiff_M_). We then looked for women-specific pathway enrichment by selecting SNPs with P_sexdiff_F_ <0.01 and *P*_*overall*_ <0.01 (given the CED framework). Similarly, we created a separate list for men-specific SNPs by a filter of P_sexdiff_M_ <0.01 and *P*_*overall*_ <0.01. All above lists were also created for age-dependent BMI associations by replacing P_sexdiff_ by P_agediff_, P_sexdiff_F_ by P_agediff_younger_ and P_sexdiff_M_ by P_agediff_older_.

For each of the eight SNP lists, leads SNPs were identified. For each lead SNP locus a target region is defined as the smallest interval containing all SNPs with LD>0.5 with the lead SNP of the locus. All genes encompassed in the target regions represent the “GWAS genes”, thereby assuming that either the lead SNP is in LD with a functional coding SNP within a gene or that the lead SNP marks a cis-acting regulatory region. We then used the following pre-defined gene sets and pathways: Gene Ontology (GO), Reactome, InWeb protein complexes, Mouse knock-out phenotypes. Gene sets were re-annotated based on co-expression in a large collection (80,000) of gene expression compendium from GEO. Then, for each gene-set the pair-wise similarity between GWAS genes was calculated and compared to that of matching sets of non-GWAS genes to assess significance of enrichment.

DEPICT also generated a prioritized set of genes at each locus. Briefly, genes within associated loci that are functionally similar to genes from other associated loci are the more likely candidates. DEPICT prioritizes genes in three steps: gene scoring, bias adjustment, and false discovery rate estimation. In the scoring step, the method quantifies the similarity of a given gene to genes from other associated loci. The bias adjustment step controls confounding factors that may bias the gene scores, e.g. gene length. In the last step, experiment-wide false discovery rates are estimated by repeating the scoring and bias adjustment steps 20 times based on top SNPs from pre-computed null GWAS.

#### Ingenuity Pathway Analysis (IPA)

Significantly associated loci were further explored using QIAGEN’s Ingenuity Pathway Analysis (IPA, QIAGEN Redwood City, www.qiagen.com/ingenuity) to determine if there was an over-arching functional or disease relationship among these loci and their associated genes using the age and sex specific SNP lists described above. IPA uses publicly available databases (e.g. NHGRI GWAS Catalog, NCBI databases, KEGG) and proprietary databases of gene/protein interaction, expression, and function to identify possible pathways, networks, and overlapping functions of genes. For our analysis, IPA identified potential genes as those genes with coding regions within 2kb upstream or 0.5kb downstream of our list of input dbSNP ids that can unambiguously be mapped to these ids. To perform the analyses, only Ingenuity Knowledge Base genes were used, both direct and indirect relationships that are observed or predicted in mammals (humans, mice, and rats) are strictly considered. All canonical pathways and functional/disease categories and annotations that were statistically significant (P < 0.05 using the Fisher’s exact test) are reported; however, those that meet significance for multiple test correction (Benjamin-Hochberg corrected P <0.05) are highlighted in the table. Only the top ten predicted networks containing up to 140 genes or endogenous molecules were requested. Only those networks with a score of greater than 2 (Fisher’s Exact Test result of P <0.01) are considered significant [92].

### Estimation of heritability

We estimated the broad heritability (H^2^) of BMI and WHR_adjBMI_ within the Family Heart Study (FHS) to assess how much of each trait’s total phenotypic variance may be genetic. A random sample of 1,810 individuals (454 families) was used for this analysis. The sample was stratified by age and sex into 9 groups (all, all ≤50y, all >50y, men, women, men ≤50y, men >50y, women ≤50y, women >50y) to assess how each trait’s genetic variance may differ across strata. Within each group, BMI and WHR_adjBMI_ were adjusted for age, age^2^, genotyping chips (Illumina 560K, 1,000,000K, 610K), 10 principal components and 3 study centers. Residuals for BMI and WHR_adjBMI_ were ranked and an inverse normal transformation was applied. Subsequently, SOLAR was used to estimate the H^2^ of BMI and WHR_adjBMI_ within each group (**S28 Table**).

### Genome-wide Complex Trait Analysis for proportion of variance explained

To explore the contribution of all common (genotyped) SNPs genome-wide to each trait of interest, BMI and WHR_adjBMI_, we estimated the variance explained by all the autosomal SNPs in the combined ARIC, KORA S3/S4, CoLaus, EGCUT and SHIP studies within each of the sex and age strata, using the method proposed by Yang et al [93] and implemented in the Genome-wide Complex Trait Analysis software package (GCTA http://www.complextraitgenomics.com/software/gcta/). Each phenotypic trait was transformed in the same form as was used for all meta-analyses.

### Estimation of explained variance

We estimated the age-group and sex-specific polygenic variance explained by various subsets of SNPs that were based on varying thresholds of overall association (P_Overall_) with BMI or WHR_adjBMI_. First, each subset of SNPs was clumped into independent regions using a physical distance criterion <500kb and for each region the most significantly overall associated SNP (i.e. top SNP) was taken further. For each top SNP, the explained variance was calculated according to

$$r^{2}=\frac{1}{1+\frac{N}{{\left(\Phi^{-1}(\frac{P}{2})\right)}^{2}}}–\frac{1-r^{2}}{N}$$

for each age-group and for each sex separately [94]. Finally, the variance explained by the subset of SNPs was obtained by summing up the single SNP-specific explained variances. The overall association threshold was varied from 1x10^-8^ to 0.1.

### Search for biological and functional knowledge of the identified association regions

We examined whether SNPs known to provide reliable tags for Copy-Number-Variations (CNVs) in subjects of European-descent (combining four catalogues including 60,167 CNV-tagging SNPs as described previously [95]) correlated with our lead SNPs. We also performed several online database searches to establish whether known variants within a 500kb-window on both sides of each lead SNP, that are in high linkage disequilibrium (r² > 0.8) with our lead SNPs (using SNAP Proxy search [96]), might have putative or predicted function. (i) We searched the SIFT database [97] to determine whether any of these SNPs were predicted to affect protein function. (ii) We used Annovar [98] to investigate predicted and putative function in several functional classes, including splicing regulation, stop codons, polyphen predictions. (iii) We used the regulome database (http://regulome.stanford.edu/) to search for known and predicted regulatory elements (DNAase hypersensitivity, binding sites of transcription factors, and promoter regions) in the intergenic regions of our age-specific BMI and sex-specific WHR_adjBMI_ loci. Additionally, we searched for estrogen, androgen or progesterone receptor motifs around our sex-specific WHR_adjBMI_ loci. Source of these data include public datasets from GEO, the ENCODE project, and published literature [99].

## Supporting Information

S1 Fig Workflow and overview of results. The numbers stated are the number of identified independent loci for the respective analysis. Given in brackets is the number of the identified loci that are novel loci for the trait, i.e. have not been previously reported for association with the trait.
(TIF)

S2 Fig QQ plots for overall association and joint test P-Values for both traits. QQ-plots for BMI (A) and WHR_adjBMI_ (B) depicting overall association P-Values (red) and joint test P-Values (blue) for all SNPs and after excluding previously published BMI or WHR_adjBMI_ associated regions (P_Overall_: magenta; P_joint_: cyan).
(TIF)

S3 Fig Locuszoom plots for 15 loci associated with BMI that are different between men and women ≤50y and men and women >50y. Each plot highlights the most significant SNP for age-differences and illustrates p-values for age-differences (P_agediff_), sex-differences (P_sexdiff_), all strata combined (P_Overall_), and the joint test (P_Joint_). The figure is sorted according to **Table 1**. The plots are based on GrCh37 build positions and annotations.
(TIF)

S4 Fig Scatterplot of effect estimates (beta) for loci showing age-differences in BMI, contrasting loci with larger effect estimates in men & women ≤50 years (light green diamonds) and loci with larger effects in men & women >50 years (dark green squares). (TIF)

S5 Fig Sensitivity meta-analysis for the 15 age-specific BMI loci-excluding 13 studies that used self-report data for BMI and comparing the age-difference effects to the originally observed age-difference. (TIF)

S6 Fig Locuszoom plots for 44 loci associated with WHR_adjBMI_ that are different between men and women. Each plot highlights the most significant SNP for sex-differences and illustrates p-values for age-differences (P_agediff_), sex-differences(P_sexdiff_), all strata combined (P_Overall_), and the joint test (P_Joint_). The figure is sorted according to **Table 2**. The plots are based on GrCh37 build positions and annotations.
(TIF)

S7 Fig Scatterplot of effect estimates (beta) for loci showing sex-differences in waist-to-hip ratio adjusted for BMI (WHR_adjBMI_), organized by loci with larger effect estimates in women compared to men (red circles), larger effect estimates in men compared to women (blue squares) and opposite effect estimates between men and women (green triangles). (TIF)

S8 Fig Sensitivity meta-analysis for the 44 sex-differential WHR_adjBMI_ loci—excluding two self-report studies and comparing the sex-difference effects to the originally observed sex-difference. (TIF)

S9 Fig Power by AGE x SEX scan. The figures illustrate the power of scanning P_sexdiff_ (A: unfiltered, B: pre-filtered on P_Overall_), P_agediff_ (C: unfiltered, D: pre-filtered on P_Overall_), and P_agesexdiff_ (E: unfiltered, F: pre-filtered on P_sexdiff_ or on P_agediff_). We assume four equally sized strata, a total sample size of N = 300,000 (comparable to the sample size in our BMI analyses). To investigate varying scenarios of interaction effects, we set (i) b_F<50y_ = 0.033, a median BMI effect near *MAP2K5* from Speliotes et al. (R^2^ = 0.037%), (ii) b_M>50y_ = 0, and (iii) vary b_F>50y_ and b_M<50y_ on the x- and y-axes respectively.
(TIF)

S10 Fig Power of the AGE x SEX approaches for BMI for varying allele frequencies and varying modelled effect sizes. The figure shows the power to detect age-difference, sex-difference or age x sex-difference in at least one of our scans and for varying scenarios of effect size combinations between the 4 strata. We assume four equally sized strata and a total sample size of N = 300,000 (comparable to the sample size in our BMI analyses). Furthermore, for each plot we (i) set b_F<50y_ to a known BMI effect sizes from Speliotes et al. paper (using a small (*PTPB2)*, medium (*NEGR1*) and the largest (*FTO*) effect size), (ii) set b_M>50y_ = 0, and (iii) vary b_F>50y_ and b_M<50y_ on the axes.
(TIF)

S11 Fig Power of the AGE x SEX approaches for WHR_adjBMI_ for varying allele frequencies and varying modelled effect sizes. The figure shows the power to detect age-difference, sex-difference or age x sex-difference in at least one of our scans and for varying scenarios of effect size combinations between the 4 strata. We assume four equally sized strata and a total sample size of N = 200,000 (comparable to the sample size in our WHR_adjBMI_ analyses). Furthermore, for each plot we (i) set b_F<50y_ to a known WHR_adjBMI_ effect sizes from Heid et al. paper (using a small (*CPEB4)*, medium (*LYPLAL1*) and the largest (*RSPO3*) effect size), (ii) set b_M>50y_ = 0, and (iii) vary b_F>50y_ and b_M<50y_ on the axes.
(TIF)

S12 Fig Differences in effect estimates (beta ±SE) between young adults, adults ≤50y, and adults >50y for BMI loci selected for age-differences. Loci are ordered according to trends in absolute magnitude of effect: 1) where the absolute magnitude of effect is largest in adolescent/youngest adults (ages 16–25y)^1^, 2) where absolute magnitude is largest in adults (≤50y), and 3) where absolute magnitude is largest in older adults (>50y). BMI: Body mass index; SE: standard error; Details for men and women ages 16–25 have been described elsewhere (Graff et al.: “Genome-wide analysis of BMI in adolescents and young adults reveals additional insight into the effects of genetic loci over the life course.” Human Molecular Genetics 2013).
(TIF)

S13 Fig The most significant SNPs, rs6088552 and rs6088735, for sex-differences with WHR_adjBMI_ each identified to be a sex-different cis-eQTL for the *ACSS2* and *MYH7B* genes, respectively, on chromosome 20. WHR_adjBMI_: waist-to-hip ratio adjusted for body-mass index; eQTL: expression quantitative trait loci. Sex-specific associations were computed to identify cis eQTL signals that were likely to be coincident with the WHR_adjBMI_ using human eQTL in lymphoblastoid cells.
(TIF)

S14 Fig Total stratum-specific explained variance by SNPs meeting varying thresholds of overall association for BMI (A: sex-specific; B: age-group specific) and for WHR_adjBMI_ (C: sex-specific; D: age-specific). (TIF)

S15 Fig Locuszoom plots for 73 novel loci associated with BMI that were either identified by the joint 4df test or by the overall (age-group and sex—combined) analysis. Each plot highlights the most significant SNP for the combined effect (P_Overall_) or for the joint test (P_Joint_) and illustrates p-values for age-differences (P_Agediff_), sex-differences (P_Sexdiff_) and P_Joint_ or P_Overall_ respectively^a^. The figure is sorted according to chromosome and position. The plots are based on GrCh37 build positions and annotations. For three loci we identified two different SNPs that met the significance threshold for the scan of P_Overall_ and P_Joint_. For each set we plotted the SNP with the lowest P-value based on the scan it was identified for. These loci and the SNP plotted are as follows: 1) rs7421089 − Selected for PJoint and rs10804189 − Selected for POverall->rs10804189 is plotted, 2) rs1557765 − Selected for POverall and rs7928810 − Selected for PJoint-> rs7928810 is plotted, and 3) rs11181001− Selected for PJoint & rs1405552− Selected for POverall-> rs1405552 is plotted.
(TIF)

S16 Fig Locuszoom plots for 10 novel loci associated with WHR_adjBMI_ that were either identified by the joint 4df test or by the overall (sex-combined) analysis. Each plot highlights the most significant SNP for the combined effect (P_Overall_) or for the joint test (P_Joint_) and illustrates p-values for age-differences (P_Agediff_), sex-differences (P_Sexdiff_) and P_Joint_ or P_Overall_ respectively. The figure is sorted according to chromosome and position. The plots are based on GrCh37 build positions and annotations.
(TIF)

S1 Table Study design, number of individuals and sample quality control for genome-wide association study cohorts. (XLSX)

S2 Table Information on genotyping methods, quality control of SNPs, imputation, and statistical analysis for genome-wide association study cohorts. (XLSX)

S3 Table Study-specific descriptive statistics of study cohorts. ** There were significant differences in the number of subjects available for different phenotypes. In this case, separate summary statistics were provided.
(XLSX)

S4 Table Stratum-specific results and extended details for the 15 age-specific BMI loci. The table is ordered according to Table 1.
(XLSX)

S5 Table Stratum-specific results and extended details for the 44 sex-specific WHR_adjBMI_ loci. The table is ordered according to Table 2.
(XLSX)

S6 Table Age-specific associations of age-dependent BMI SNPs with blood pressure (BP). Abbreviations: Effect Allele (EA), Other Allele (OA), SNP-by-age interaction effect (b_SNPxAGE_), SNP-by-age interaction effect standard error (SE_SNPxAGE_), SNP-by-age interaction P-value (P_SNPxAGE_).
(XLSX)

S7 Table Sex-specific associations of sexually dimorphic WHR_adjBMI_ SNPs with lipid traits (GLGC). Abbreviations: Effect Alelle (EA), Other Allele (OA), High Density Lipoprotein Cholesterol (HDL), Low Density Lipoprotein (LDL), Total Cholesterol (TC), Triglycerides (TG).
(XLSX)

S8 Table Sex-specific associations of sexually dimorphic WHR_adjBMI_ SNPs with Type 2 Diabetes (T2D, DIAGRAM) and glycemic traits (MAGIC). Abbreviations: Effect Allele (EA), Other Allele (OA), Odds Ratio (OR).
(XLSX)

S9 Table Sex-specific associations of sexually dimorphic WHR_adjBMI_ SNPs with blood pressure (BP) measures. Abbreviations: Effect Allele (EA), Other Allele (OA), Odds Ratio (OR).
(XLSX)

S10 Table Remarkable women-specific associations in the WHR_adjBMI_ lookup data. The table shows SNPs that meet a Bonferroni-corrected significance level (<0.05/44) for its sex-specific association with the lookup trait and no association with the lookup trait in the other sex. Only women-specific loci displayed similar patterns in the look-up data. None of the opposite effect direction loci showed opposite effects (requesting P<0.05 in both sexes) with a look-up trait.
(XLSX)

S11 Table Previously-identified associations listed in the NHGRI GWAS Catalog that lie within 500 kb and r^2^ > 0.1 to our lead BMI SNPs. (XLSX)

S12 Table Previously-identified associations listed in the NHGRI GWAS Catalog that lie within 500 kb and r^2^ > 0.1 to our lead WHR_adjBMI_ SNPs. (XLSX)

S13 Table BMI loci showing significant age-differences in adults < = 50y compared to adults >50y. Analysis was restricted to non-case control studies.
(XLSX)

S14 Table WHR_adjBMI_ loci showing significant sex-differences. Analysis was restricted to non-case control studies.
(XLSX)

S15 Table Effect estimates of BMI loci selected for age-differences and birthweight from 26,836 participants in the EGG consortium. (XLSX)

S16 Table Odds ratios of BMI loci selected for age-differences and childhood obesity from 5,530 cases and 8,318 controls in the EGG consortium. (XLSX)

S17 Table Differences in effect estimates between young adults, adults < = 50y, and adults >50y for BMI loci selected for age-differences. (XLSX)

S18 Table Effect estimates for weight change trajectories in adults between the ages of 20 and 65 years of age in loci showing effect size differences in BMI by age. (XLSX)

S19 Table Top hits of the human sex-specific eQTL lookup of the sex-specific WHR_adjBMI_ associated SNPs. Presented are SNPs with significant sex-differences in eQTL effects, selected according to FDR(P-Sexdiff) < 5%, with and without initial filtering on overall expression effects (FDR(P-Overall) < 20%).
(XLSX)

S20 Table List of tissues in which DEPICT identified significant expression (FDR <0.1) of genes from age-specific BMI associated loci in at least one of the four approaches. (XLSX)

S21 Table Gene sets enriched (FDR<0.1) for harboring SNPs with sex-different effect on WHR_adjBMI_ identified by DEPICT. (XLSX)

S22 Table All significant function or disease annotations identified in IPA for the younger adult-specific BMI loci. Functions that remain significant after B-H (p<0.05) correction are marked in bold.
(XLSX)

S23 Table All significant function or disease annotations identified in IPA for the older adult-specific BMI loci. Functions that remain significant after B-H correction are marked in bold.
(XLSX)

S24 Table All significant canonical pathways identified in IPA for the women-specific WHR_adjBMI_ loci. Pathways that remain significant after B-H correction are marked in bold.
(XLSX)

S25 Table All significant function or disease annotations identified in IPA for the women-specific WHR_adjBMI_ loci. Functions that remain significant after B-H correction are marked in bold.
(XLSX)

S26 Table All significant function or disease annotations identified in IPA for the men-specific WHR_adjBMI_ loci. Functions that remain significant after B-H correction are marked in bold.
(XLSX)

S27 Table Proportion of genetic to phenotypic variance explained for BMI, and WHR_adjBMI_ estimated using GCTA and Heritability estimated using SOLAR. (XLSX)

S28 Table BMI main or joint (main+interaction, 4df) effect findings compared to results from the GIANT BMI group [19]. (XLSX)

S29 Table WHR_adjBMI_ main or joint (main+interaction, 4df) effect findings compared to results from the GIANT WAIST group [18]. (XLSX)

S1 Text Supplementary note. (DOCX)

S2 Text Consortia members and extended acknowledgments. (DOCX)
