# Correction: The Influence of Age and Sex on Genetic Associations with Adult Body Size and Shape: A Large-Scale Genome-Wide Interaction Study

**DOI:** 10.1371/journal.pgen.1006166

**Published:** 2016-06-29

**Authors:** Thomas W. Winkler, Anne E. Justice, Mariaelisa Graff, Llilda Barata, Mary F. Feitosa, Su Chu, Jacek Czajkowski, Tõnu Esko, Tove Fall, Tuomas O. Kilpeläinen, Yingchang Lu, Reedik Mägi, Evelin Mihailov, Tune H. Pers, Sina Rüeger, Alexander Teumer, Georg B. Ehret, Teresa Ferreira, Nancy L. Heard-Costa, Juha Karjalainen, Vasiliki Lagou, Anubha Mahajan, Michael D. Neinast, Inga Prokopenko, Jeannette Simino, Tanya M. Teslovich, Rick Jansen, Harm-Jan Westra, Charles C. White, Devin Absher, Tarunveer S. Ahluwalia, Shafqat Ahmad, Eva Albrecht, Alexessander Couto Alves, Jennifer L. Bragg-Gresham, Anton J. M. de Craen, Joshua C. Bis, Amélie Bonnefond, Gabrielle Boucher, Gemma Cadby, Yu-Ching Cheng, Charleston W. K. Chiang, Graciela Delgado, Ayse Demirkan, Nicole Dueker, Niina Eklund, Gudny Eiriksdottir, Joel Eriksson, Bjarke Feenstra, Krista Fischer, Francesca Frau, Tessel E. Galesloot, Frank Geller, Anuj Goel, Mathias Gorski, Tanja B. Grammer, Stefan Gustafsson, Saskia Haitjema, Jouke-Jan Hottenga, Jennifer E. Huffman, Anne U. Jackson, Kevin B. Jacobs, Åsa Johansson, Marika Kaakinen, Marcus E. Kleber, Jari Lahti, Irene Mateo Leach, Benjamin Lehne, Youfang Liu, Ken Sin Lo, Mattias Lorentzon, Jian'an Luan, Pamela A. F. Madden, Massimo Mangino, Barbara McKnight, Carolina Medina-Gomez, Keri L. Monda, May E. Montasser, Gabriele Müller, Martina Müller-Nurasyid, Ilja M. Nolte, Kalliope Panoutsopoulou, Laura Pascoe, Lavinia Paternoster, Nigel W. Rayner, Frida Renström, Federica Rizzi, Lynda M. Rose, Kathy A. Ryan, Perttu Salo, Serena Sanna, Hubert Scharnagl, Jianxin Shi, Albert Vernon Smith, Lorraine Southam, Alena Stančáková, Valgerdur Steinthorsdottir, Rona J. Strawbridge, Yun Ju Sung, Ioanna Tachmazidou, Toshiko Tanaka, Gudmar Thorleifsson, Stella Trompet, Natalia Pervjakova, Jonathan P. Tyrer, Liesbeth Vandenput, Sander W van der Laan, Nathalie van der Velde, Jessica van Setten, Jana V. van Vliet-Ostaptchouk, Niek Verweij, Efthymia Vlachopoulou, Lindsay L. Waite, Sophie R. Wang, Zhaoming Wang, Sarah H. Wild, Christina Willenborg, James F. Wilson, Andrew Wong, Jian Yang, Loïc Yengo, Laura M. Yerges-Armstrong, Lei Yu, Weihua Zhang, Jing Hua Zhao, Ehm A. Andersson, Stephan J. L. Bakker, Damiano Baldassarre, Karina Banasik, Matteo Barcella, Cristina Barlassina, Claire Bellis, Paola Benaglio, John Blangero, Matthias Blüher, Fabrice Bonnet, Lori L. Bonnycastle, Heather A. Boyd, Marcel Bruinenberg, Aron S Buchman, Harry Campbell, Yii-Der Ida Chen, Peter S. Chines, Simone Claudi-Boehm, John Cole, Francis S. Collins, Eco J. C. de Geus, Lisette C. P. G. M. de Groot, Maria Dimitriou, Jubao Duan, Stefan Enroth, Elodie Eury, Aliki-Eleni Farmaki, Nita G. Forouhi, Nele Friedrich, Pablo V. Gejman, Bruna Gigante, Nicola Glorioso, Alan S. Go, Omri Gottesman, Jürgen Gräßler, Harald Grallert, Niels Grarup, Yu-Mei Gu, Linda Broer, Annelies C. Ham, Torben Hansen, Tamara B. Harris, Catharina A. Hartman, Maija Hassinen, Nicholas Hastie, Andrew T. Hattersley, Andrew C. Heath, Anjali K. Henders, Dena Hernandez, Hans Hillege, Oddgeir Holmen, Kees G Hovingh, Jennie Hui, Lise L. Husemoen, Nina Hutri-Kähönen, Pirro G. Hysi, Thomas Illig, Philip L. De Jager, Shapour Jalilzadeh, Torben Jørgensen, J. Wouter Jukema, Markus Juonala, Stavroula Kanoni, Maria Karaleftheri, Kay Tee Khaw, Leena Kinnunen, Steven J. Kittner, Wolfgang Koenig, Ivana Kolcic, Peter Kovacs, Nikolaj T. Krarup, Wolfgang Kratzer, Janine Krüger, Diana Kuh, Meena Kumari, Theodosios Kyriakou, Claudia Langenberg, Lars Lannfelt, Chiara Lanzani, Vaneet Lotay, Lenore J. Launer, Karin Leander, Jaana Lindström, Allan Linneberg, Yan-Ping Liu, Stéphane Lobbens, Robert Luben, Valeriya Lyssenko, Satu Männistö, Patrik K. Magnusson, Wendy L. McArdle, Cristina Menni, Sigrun Merger, Lili Milani, Grant W. Montgomery, Andrew P. Morris, Narisu Narisu, Mari Nelis, Ken K. Ong, Aarno Palotie, Louis Pérusse, Irene Pichler, Maria G. Pilia, Anneli Pouta, Myriam Rheinberger, Rasmus Ribel-Madsen, Marcus Richards, Kenneth M. Rice, Treva K. Rice, Carlo Rivolta, Veikko Salomaa, Alan R. Sanders, Mark A. Sarzynski, Salome Scholtens, Robert A. Scott, William R. Scott, Sylvain Sebert, Sebanti Sengupta, Bengt Sennblad, Thomas Seufferlein, Angela Silveira, P. Eline Slagboom, Jan H. Smit, Thomas H. Sparsø, Kathleen Stirrups, Ronald P. Stolk, Heather M. Stringham, Morris A Swertz, Amy J. Swift, Ann-Christine Syvänen, Sian-Tsung Tan, Barbara Thorand, Anke Tönjes, Angelo Tremblay, Emmanouil Tsafantakis, Peter J. van der Most, Uwe Völker, Marie-Claude Vohl, Judith M. Vonk, Melanie Waldenberger, Ryan W. Walker, Roman Wennauer, Elisabeth Widén, Gonneke Willemsen, Tom Wilsgaard, Alan F. Wright, M. Carola Zillikens, Suzanne C. van Dijk, Natasja M. van Schoor, Folkert W. Asselbergs, Paul I. W. de Bakker, Jacques S. Beckmann, John Beilby, David A. Bennett, Richard N. Bergman, Sven Bergmann, Carsten A. Böger, Bernhard O. Boehm, Eric Boerwinkle, Dorret I. Boomsma, Stefan R. Bornstein, Erwin P. Bottinger, Claude Bouchard, John C. Chambers, Stephen J. Chanock, Daniel I. Chasman, Francesco Cucca, Daniele Cusi, George Dedoussis, Jeanette Erdmann, Johan G. Eriksson, Denis A. Evans, Ulf de Faire, Martin Farrall, Luigi Ferrucci, Ian Ford, Lude Franke, Paul W. Franks, Philippe Froguel, Ron T. Gansevoort, Christian Gieger, Henrik Grönberg, Vilmundur Gudnason, Ulf Gyllensten, Per Hall, Anders Hamsten, Pim van der Harst, Caroline Hayward, Markku Heliövaara, Christian Hengstenberg, Andrew A Hicks, Aroon Hingorani, Albert Hofman, Frank Hu, Heikki V. Huikuri, Kristian Hveem, Alan L. James, Joanne M. Jordan, Antti Jula, Mika Kähönen, Eero Kajantie, Sekar Kathiresan, Lambertus A. L. M. Kiemeney, Mika Kivimaki, Paul B. Knekt, Heikki A. Koistinen, Jaspal S. Kooner, Seppo Koskinen, Johanna Kuusisto, Winfried Maerz, Nicholas G Martin, Markku Laakso, Timo A. Lakka, Terho Lehtimäki, Guillaume Lettre, Douglas F. Levinson, Lars Lind, Marja-Liisa Lokki, Pekka Mäntyselkä, Mads Melbye, Andres Metspalu, Braxton D. Mitchell, Frans L. Moll, Jeffrey C. Murray, Arthur W. Musk, Markku S. Nieminen, Inger Njølstad, Claes Ohlsson, Albertine J. Oldehinkel, Ben A. Oostra, Lyle J Palmer, James S. Pankow, Gerard Pasterkamp, Nancy L. Pedersen, Oluf Pedersen, Brenda W. Penninx, Markus Perola, Annette Peters, Ozren Polašek, Peter P. Pramstaller, Bruce M. Psaty, Lu Qi, Thomas Quertermous, Olli T. Raitakari, Tuomo Rankinen, Rainer Rauramaa, Paul M. Ridker, John D. Rioux, Fernando Rivadeneira, Jerome I. Rotter, Igor Rudan, Hester M. den Ruijter, Juha Saltevo, Naveed Sattar, Heribert Schunkert, Peter E. H. Schwarz, Alan R. Shuldiner, Juha Sinisalo, Harold Snieder, Thorkild I. A. Sørensen, Tim D. Spector, Jan A. Staessen, Bandinelli Stefania, Unnur Thorsteinsdottir, Michael Stumvoll, Jean-Claude Tardif, Elena Tremoli, Jaakko Tuomilehto, André G. Uitterlinden, Matti Uusitupa, André L. M. Verbeek, Sita H. Vermeulen, Jorma S. Viikari, Veronique Vitart, Henry Völzke, Peter Vollenweider, Gérard Waeber, Mark Walker, Henri Wallaschofski, Nicholas J. Wareham, Hugh Watkins, Eleftheria Zeggini, arcOGEN Consortium, CHARGE Consortium, DIAGRAM Consortium, GLGC Consortium, Global-BPGen Consortium, ICBP Consortium, MAGIC Consortium, Aravinda Chakravarti, Deborah J. Clegg, L. Adrienne Cupples, Penny Gordon-Larsen, Cashell E. Jaquish, D. C. Rao, Goncalo R. Abecasis, Themistocles L. Assimes, Inês Barroso, Sonja I. Berndt, Michael Boehnke, Panos Deloukas, Caroline S. Fox, Leif C. Groop, David J. Hunter, Erik Ingelsson, Robert C. Kaplan, Mark I. McCarthy, Karen L. Mohlke, Jeffrey R. O'Connell, David Schlessinger, David P. Strachan, Kari Stefansson, Cornelia M. van Duijn, Joel N. Hirschhorn, Cecilia M. Lindgren, Iris M. Heid, Kari E. North, Ingrid B. Borecki, Zoltán Kutalik, Ruth J. F. Loos

The arcOGEN Consortium should be listed as an author of this article. They contributed to the genome-wide association study results presented in this work. They should be listed in the author byline at position 292 and affiliated with The Arthritis Research UK Osteoarthritis Genetics Consortium. They should also be included in the footnote designating consortia which is underneath the author affiliation list in the PDF version of the article, and in the S2 Text. Please view the correct S2 Text below, containing correct consortia members.

## Supporting Information

S2 Text Consortia members and extended acknowledgments. (DOCX)
