# Supplementary figures and images for: The Influence of Age and Sex on Genetic Associations with Adult Body Size and Shape: A Large-Scale Genome-Wide Interaction Study
Source: PLoS Genet. 2015 Oct 1;11(10):e1005378. doi: 10.1371/journal.pgen.1005378 (PMC4591371; doi:10.1371/journal.pgen.1005378)

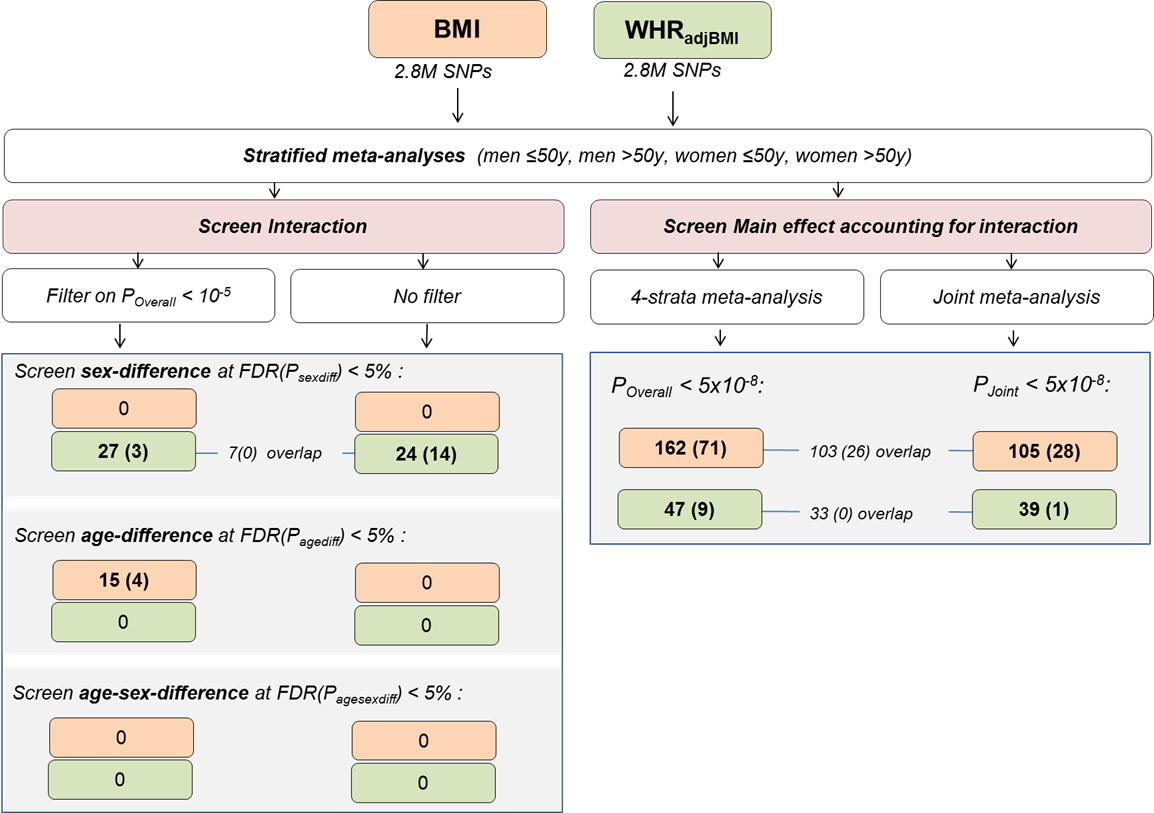

Supplement: S1 Fig — The numbers stated are the number of identified independent loci for the respective analysis. Given in brackets is the number of the identified loci that are novel loci for the trait, i.e. have not been previously reported for association with the trait. (TIF) [file pgen.1005378.s001.tif]

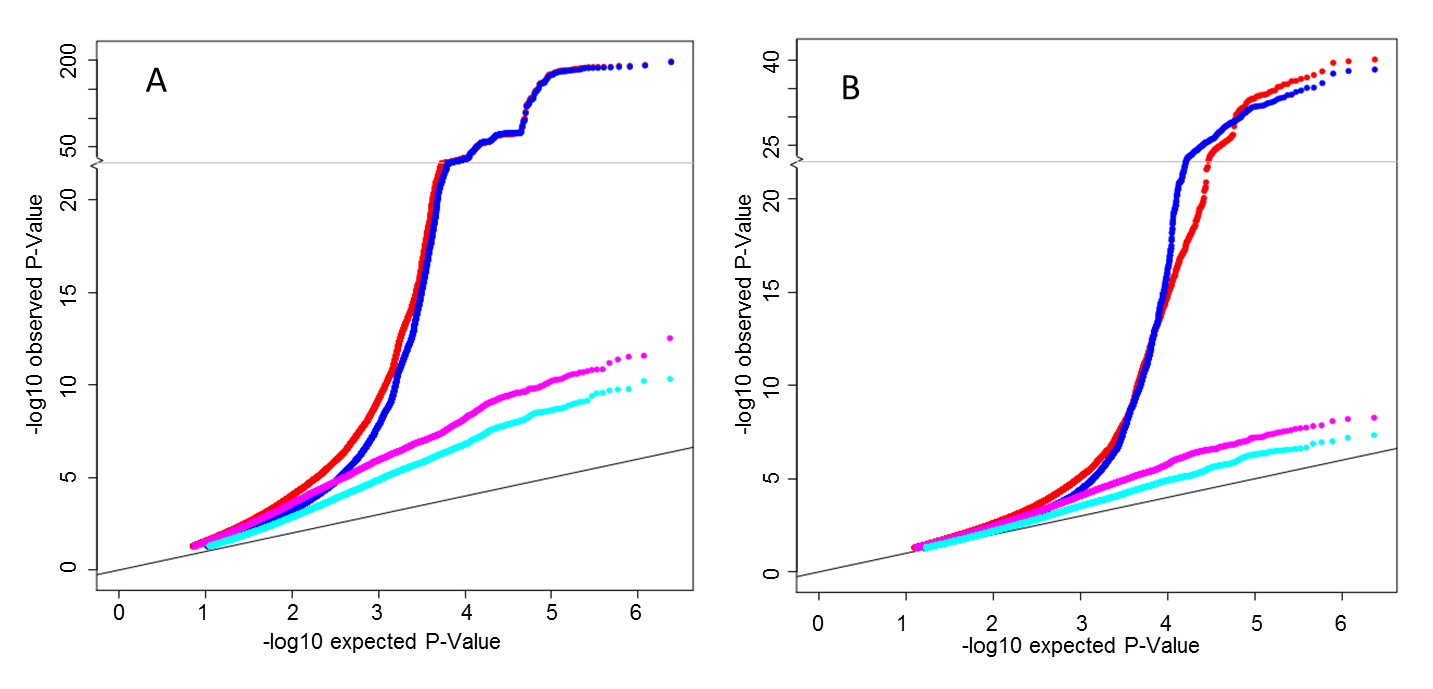

Supplement: S2 Fig — QQ-plots for BMI (A) and WHRadjBMI (B) depicting overall association P-Values (red) and joint test P-Values (blue) for all SNPs and after excluding previously published BMI or WHRadjBMI associated regions (POverall: magenta; Pjoint: cyan). (TIF) [file pgen.1005378.s002.tif]

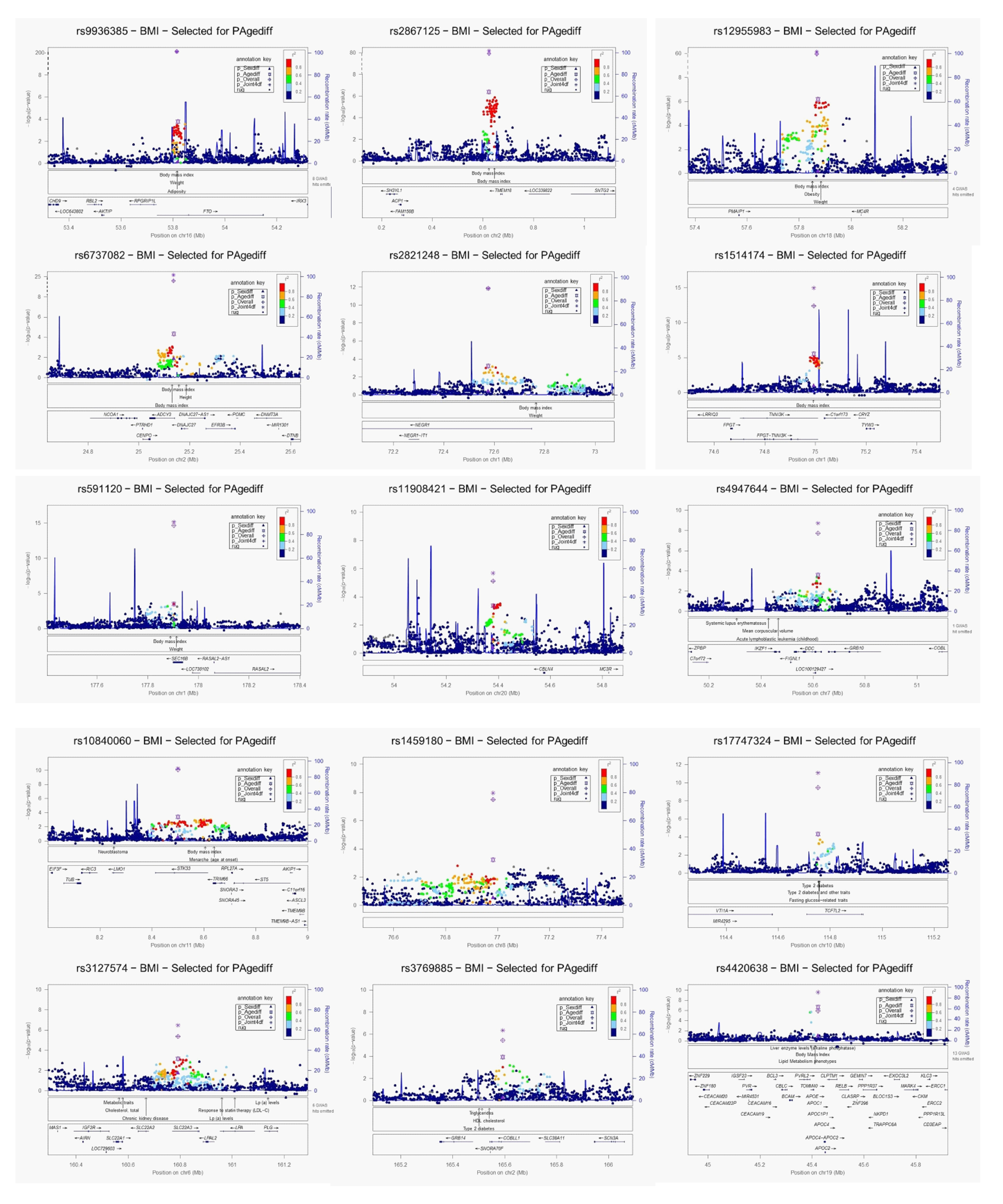

Supplement: S3 Fig — Each plot highlights the most significant SNP for age-differences and illustrates p-values for age-differences (Pagediff), sex-differences (Psexdiff), all strata combined (POverall), and the joint test (PJoint). The figure is sorted according to Table 1. The plots are based on GrCh37 build positions and annotations. (TIF) [file pgen.1005378.s003.tif]

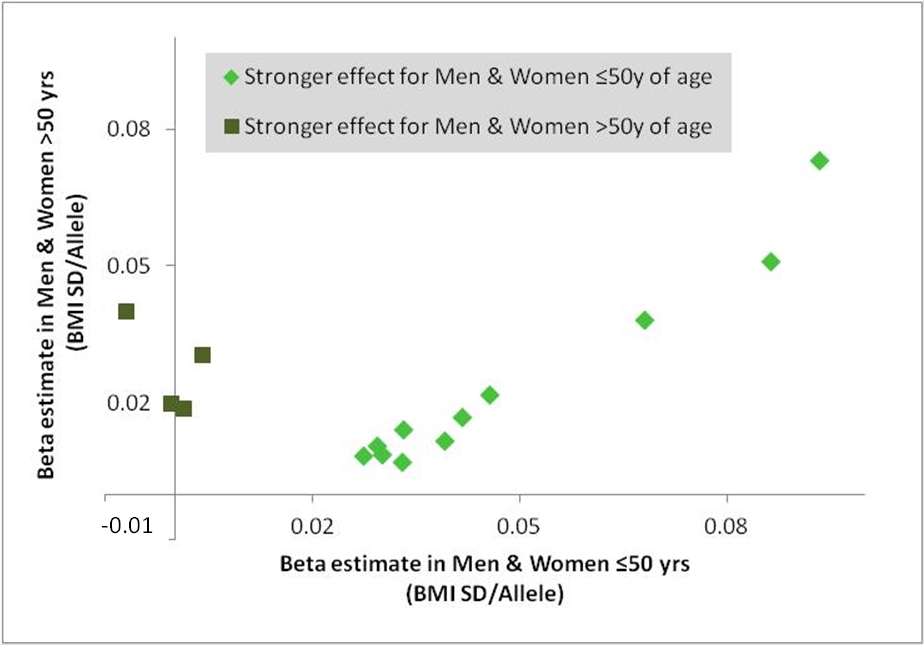

Supplement: S4 Fig — (TIF) [file pgen.1005378.s004.tif]

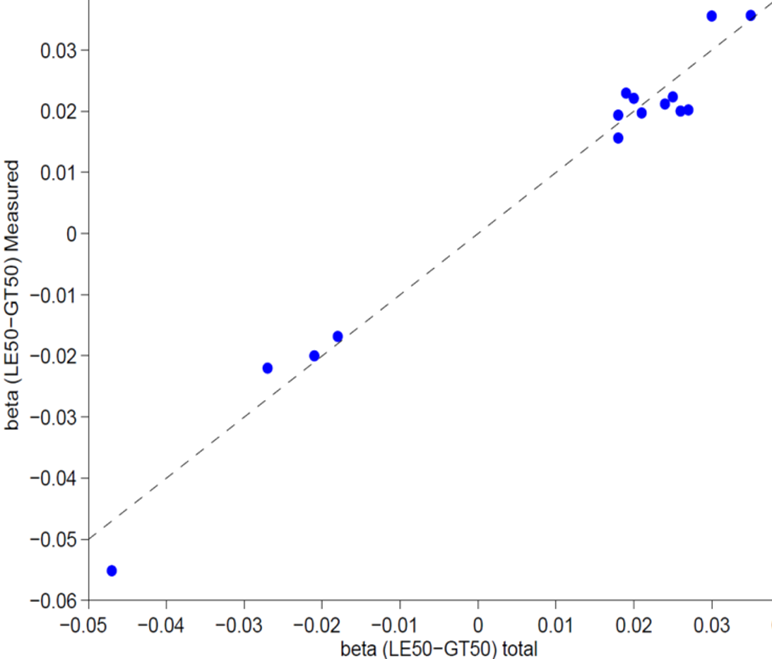

Supplement: S5 Fig — (TIF) [file pgen.1005378.s005.tif]

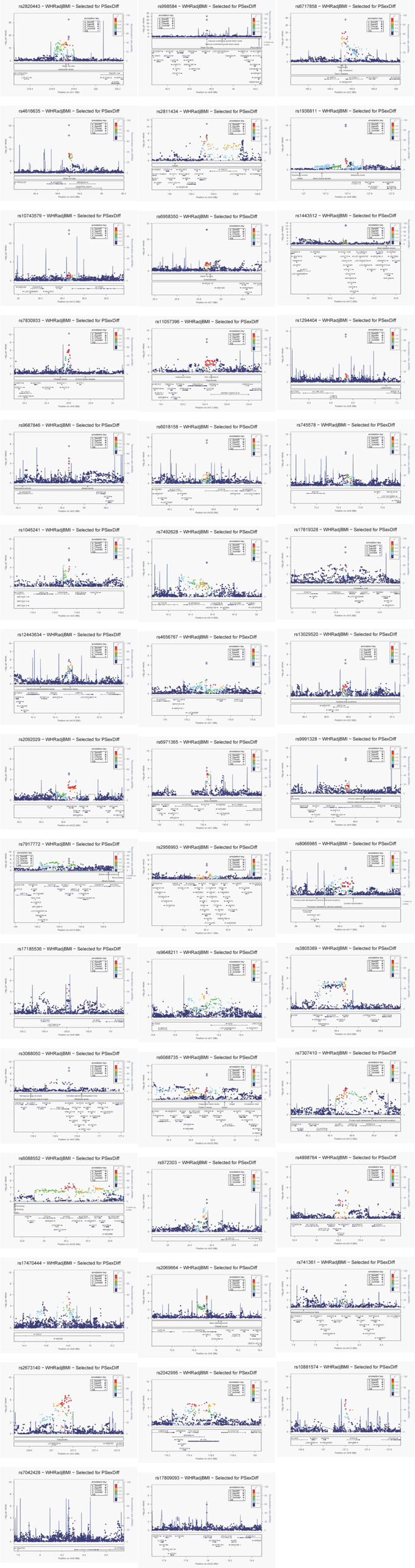

Supplement: S6 Fig — Each plot highlights the most significant SNP for sex-differences and illustrates p-values for age-differences (Pagediff), sex-differences(Psexdiff), all strata combined (POverall), and the joint test (PJoint). The figure is sorted according to Table 2. The plots are based on GrCh37 build positions and annotations. (TIF) [file pgen.1005378.s006.tif]

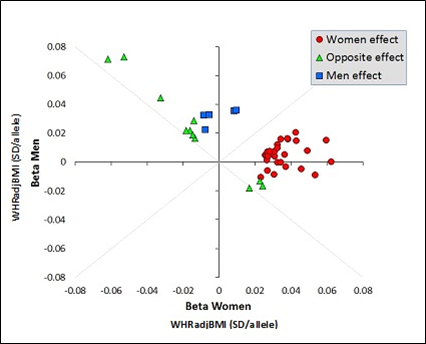

Supplement: S7 Fig — (TIF) [file pgen.1005378.s007.tif]

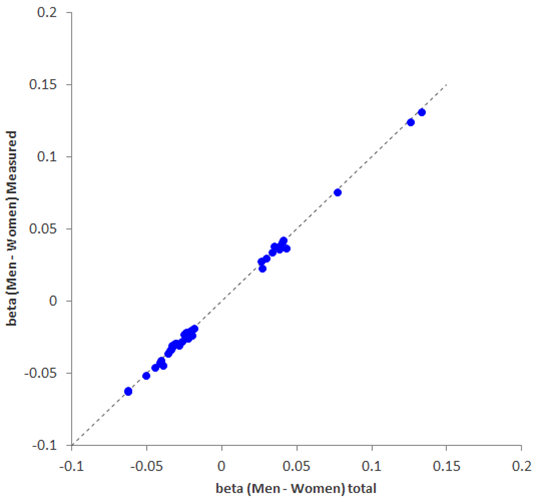

Supplement: S8 Fig — (TIF) [file pgen.1005378.s008.tif]

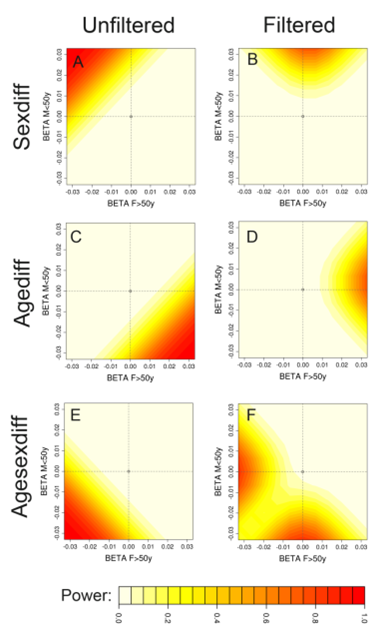

Supplement: S9 Fig — The figures illustrate the power of scanning Psexdiff (A: unfiltered, B: pre-filtered on POverall), Pagediff (C: unfiltered, D: pre-filtered on POverall), and Pagesexdiff (E: unfiltered, F: pre-filtered on Psexdiff or on Pagediff). We assume four equally sized strata, a total sample size of N = 300,000 (comparable to the sample size in our BMI analyses). To investigate varying scenarios of interaction effects, we set (i) bF<50y = 0.033, a median BMI effect near MAP2K5 from Speliotes et al. (R2 = 0.037%), (ii) bM>50y = 0, and (iii) vary bF>50y and bM<50y on the x- and y-axes respectively. (TIF) [file pgen.1005378.s009.tif]

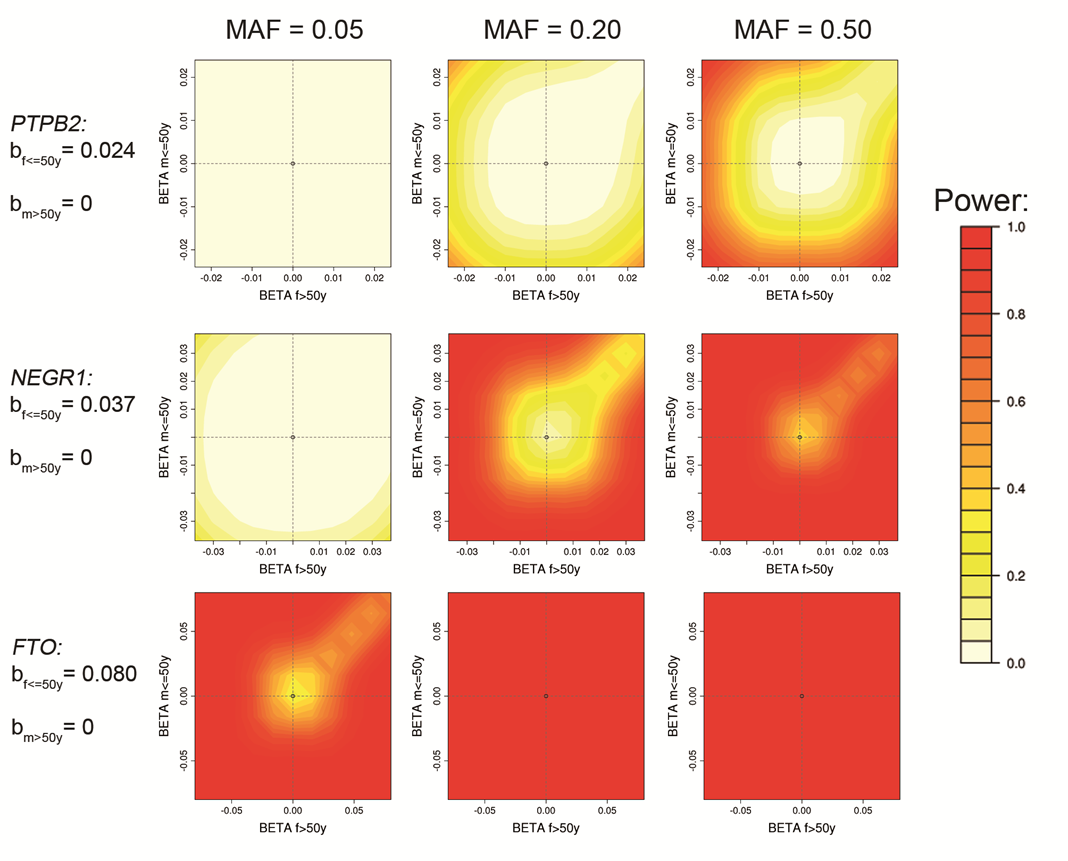

Supplement: S10 Fig — The figure shows the power to detect age-difference, sex-difference or age x sex-difference in at least one of our scans and for varying scenarios of effect size combinations between the 4 strata. We assume four equally sized strata and a total sample size of N = 300,000 (comparable to the sample size in our BMI analyses). Furthermore, for each plot we (i) set bF<50y to a known BMI effect sizes from Speliotes et al. paper (using a small (PTPB2), medium (NEGR1) and the largest (FTO) effect size), (ii) set bM>50y = 0, and (iii) vary bF>50y and bM<50y on the axes. (TIF) [file pgen.1005378.s010.tif]

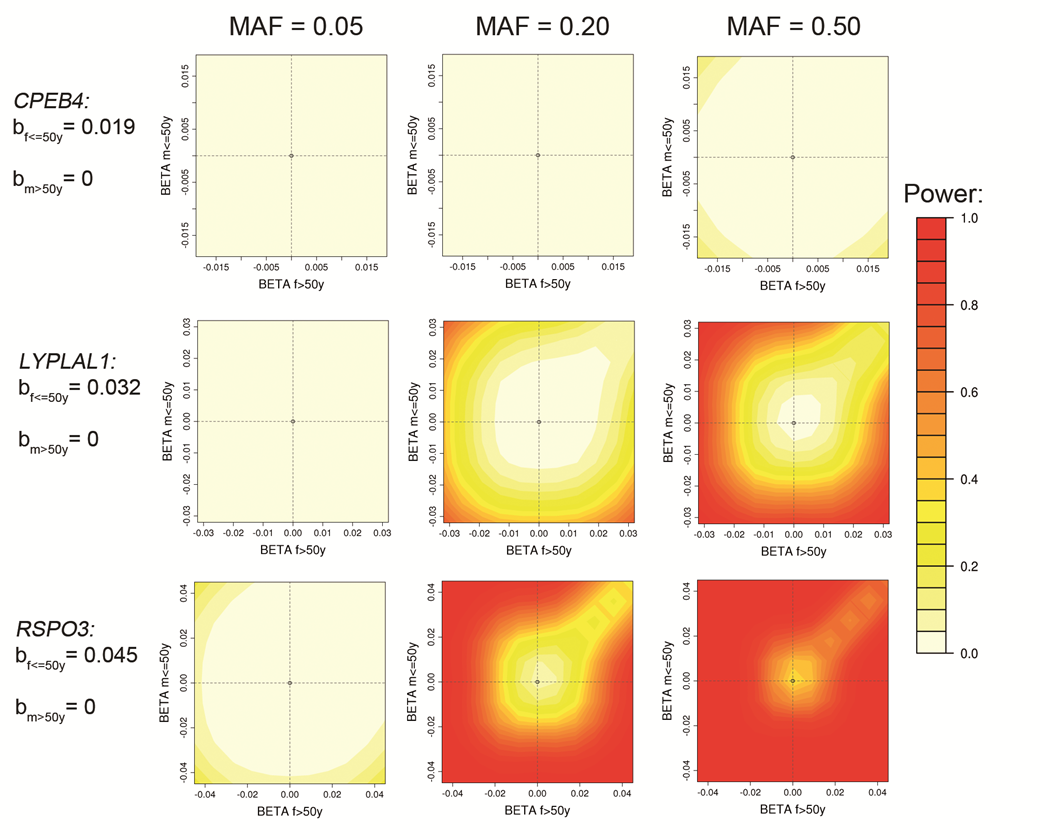

Supplement: S11 Fig — The figure shows the power to detect age-difference, sex-difference or age x sex-difference in at least one of our scans and for varying scenarios of effect size combinations between the 4 strata. We assume four equally sized strata and a total sample size of N = 200,000 (comparable to the sample size in our WHRadjBMI analyses). Furthermore, for each plot we (i) set bF<50y to a known WHRadjBMI effect sizes from Heid et al. paper (using a small (CPEB4), medium (LYPLAL1) and the largest (RSPO3) effect size), (ii) set bM>50y = 0, and (iii) vary bF>50y and bM<50y on the axes. (TIF) [file pgen.1005378.s011.tif]

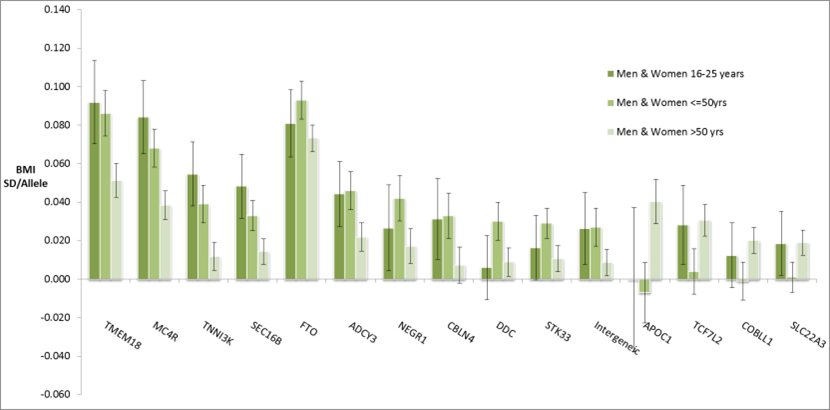

Supplement: S12 Fig — Loci are ordered according to trends in absolute magnitude of effect: 1) where the absolute magnitude of effect is largest in adolescent/youngest adults (ages 16–25y)1, 2) where absolute magnitude is largest in adults (≤50y), and 3) where absolute magnitude is largest in older adults (>50y). BMI: Body mass index; SE: standard error; Details for men and women ages 16–25 have been described elsewhere (Graff et al.: “Genome-wide analysis of BMI in adolescents and young adults reveals additional insight into the effects of genetic loci over the life course.” Human Molecular Genetics 2013). (TIF) [file pgen.1005378.s012.tif]

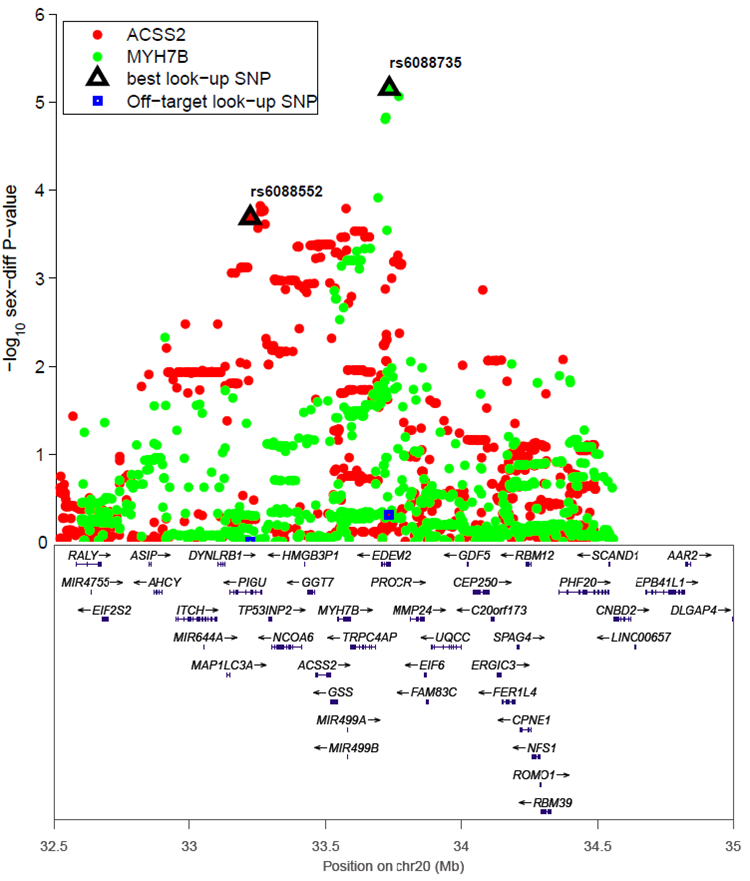

Supplement: S13 Fig — WHRadjBMI: waist-to-hip ratio adjusted for body-mass index; eQTL: expression quantitative trait loci. Sex-specific associations were computed to identify cis eQTL signals that were likely to be coincident with the WHRadjBMI using human eQTL in lymphoblastoid cells. (TIF) [file pgen.1005378.s013.tif]

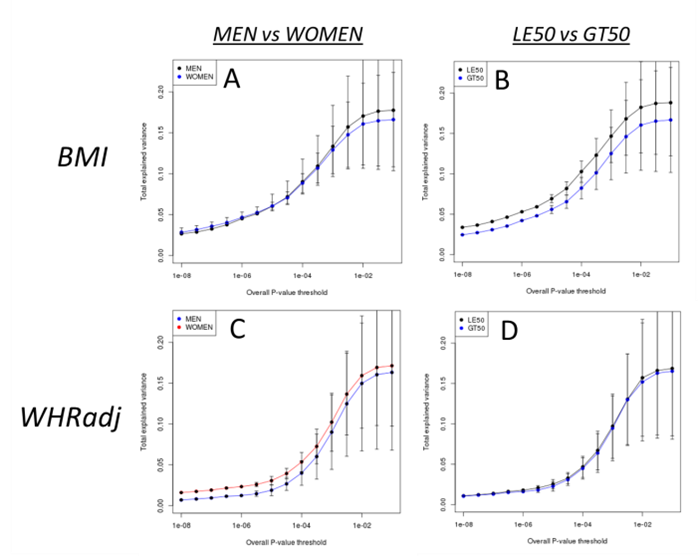

Supplement: S14 Fig — (TIF) [file pgen.1005378.s014.tif]

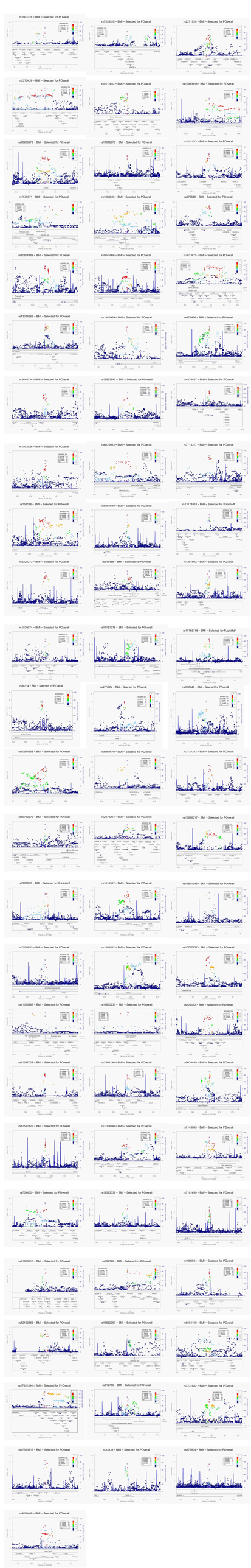

Supplement: S15 Fig — Each plot highlights the most significant SNP for the combined effect (POverall) or for the joint test (PJoint) and illustrates p-values for age-differences (PAgediff), sex-differences (PSexdiff) and PJoint or POverall respectivelya. The figure is sorted according to chromosome and position. The plots are based on GrCh37 build positions and annotations. For three loci we identified two different SNPs that met the significance threshold for the scan of POverall and PJoint. For each set we plotted the SNP with the lowest P-value based on the scan it was identified for. These loci and the SNP plotted are as follows: 1) rs7421089 − Selected for PJoint and rs10804189 − Selected for POverall->rs10804189 is plotted, 2) rs1557765 − Selected for POverall and rs7928810 − Selected for PJoint-> rs7928810 is plotted, and 3) rs11181001− Selected for PJoint & rs1405552− Selected for POverall-> rs1405552 is plotted. (TIF) [file pgen.1005378.s015.tif]

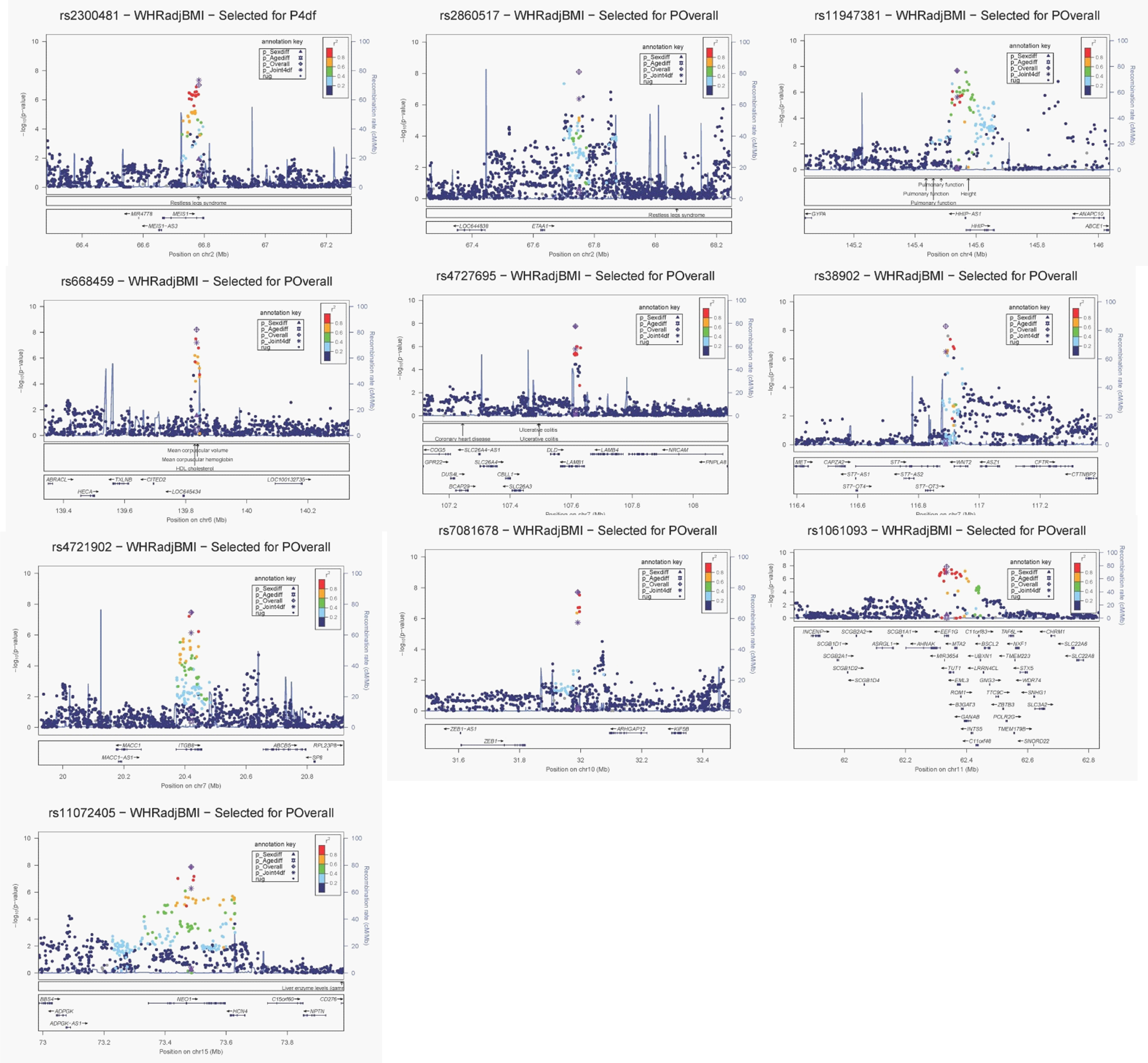

Supplement: S16 Fig — Each plot highlights the most significant SNP for the combined effect (POverall) or for the joint test (PJoint) and illustrates p-values for age-differences (PAgediff), sex-differences (PSexdiff) and PJoint or POverall respectively. The figure is sorted according to chromosome and position. The plots are based on GrCh37 build positions and annotations. (TIF) [file pgen.1005378.s016.tif]
