# Supplementary material for: The Influence of Age and Sex on Genetic Associations with Adult Body Size and Shape: A Large-Scale Genome-Wide Interaction Study
Source: PLoS Genet. 2015 Oct 1;11(10):e1005378. doi: 10.1371/journal.pgen.1005378 (PMC4591371; doi:10.1371/journal.pgen.1005378)
Supplement: S1 Text — (DOCX) [file pgen.1005378.s046.docx]

**Supplementary Note**

**Look up in GWAS for BMI of individuals aged 16-25 years**

To further examine the evidence for age-specific effects across the life course, we examined the 15 variants with age-specific effects on BMI in a previously performed GWA meta-analysis for BMI in individuals (N = up to 29,880) aged 16 to 25 years [[1](#_ENREF_1)] and compared effect sizes from this study with the two age strata from the present study (16-25y, 18-50y, >50y) (**S10 Figure**, **S17 Table**).

All 11 loci with stronger effects in younger adults (18-50y) compared to older adults (>50y) showed directionally consistent association with BMI in the 16-25y old group, nine of which reached nominal significance: Four of the nine loci (*TMEM18, MC4R, TNNI3K, SEC16B*) had a more pronounced effect in the 16-25y age group, supporting a decreasing trend in effect size across the three age groups, and the remaining five showed no difference between the youngest (16-25y) and the younger adults (18-50y) group. The *DDC* and the *STK33* locus had only a weak effect on BMI in the 16-25y group, which increased in the 18-50y adults, and then decreased in the >50y group, resulting in an inverse U-shaped trend (*P* = 0.0015 < 0.05/15, Bonferroni corrected).

In summary, the data from the youngest age-group (16-25y) did not provide strong evidence that the age-trends seen for adults could be extrapolated to young adults. This might be due to our currently limited understanding of genetic influences on BMI across the life course and/or because of the relatively small sample of the youngest age group. The lack of evidence may also be due to the substantial overlap in age and in individuals between the youngest (16-25y) and the young adult (18-50y) groups. Specifically, nine of the 14 youngest cohorts were also part of our young adult sample. A larger-scale GWAS on BMI at a younger age in childhood might be needed to observe more distinct trends across the life course.

**Gene set and tissue or cell type enrichment analysis with DEPICT**

For BMI, DEPICT identified “abdominal subcutaneous fat”, “abdominal fat”, and “adipose tissue” as tissues in which genes from age-specific BMI associated loci were highly expressed (**S20 Table**). No significantly enriched sets for the genes harboring loci with BMI age-specific effects were identified. For WHR_adjBMI,_ no tissues were significantly enriched for expression of sex-specific WHR_adjBMI_ associated loci. However, several gene sets enriched for SNPs with sex-dependent effect on WHR_adjBMI_ were identified (**S21 Table**), mostly involving transcriptional regulation (e.g. SMAD3 protein complex, STAT2 protein complex).

**Pathway analyses and functional annotation with IPA**

While there were no significant canonical pathways, we did observe significant enrichment for disease and functional annotations for genes harbored by loci that show age-specific effects for BMI (**S22-S23 Tables**). Genes in these age-specific BMI loci were highly enriched for cancer and functions related to cell morphology and nervous system development and function (at FDR < 5%). Loci that showed larger effects in older adults were enriched for genes influencing behavior (e.g. social and learning behaviors, and flexibility in behavior), and metabolic and gastrointestinal disorders likely to influence weight later in life, e.g. Crohn’s disease or type 2 diabetes (at FDR < 5%).

A total of 45 canonical pathways reached significance (at FDR < 5%) for the WHR_adjBMI_ SNPs that exhibited a greater effect in women (**S24 Table**), of which PPARα/RXRα activation was the most significant. While genes in loci with more pronounced effects in women vs men and vice versa were significantly enriched for functional categories related to organismal and cellular growth and development, there are several differences between these gene sets (**S25-S26 Tables**). The loci that show more pronounced effect in men were enriched for disease and abnormal morphology annotations; while those with greater effects in women were significantly enriched for other functional categories, including energy production, lipid and carbohydrate metabolism, behavior, reproductive system and development and small molecule biochemistry (including those related to homeostasis of steroids and lipids).

**Functional follow-up of the identified loci via bioinformatics tools**

Only one sex-dependent SNP associated with WHR_adjBMI_ (rs6088552) near the gene *PIGU* on chromosome 20 was found to tag a nearby CNV (chr20: 32,705,988-32,707,384). Using RegulomeDB, evidence of regulatory activity was found for two WHR_adjBMI_ associated SNPs (near *NSD1* and *EDEM2)* and for two BMI associated loci (near *ADCY3* and *STK33*)*,* all involving eQTL, TF binding and DNase peak evidence. Finally, estrogen receptor motifs were found in vicinity of seven of our sex-specific WHR_adjBMI_ SNPs (near *PLXND1, IRS1, ITPR2, HOXC13, HMGXB4, LY86, NFE2L3*, and *CCDC92*).

**REFERENCES**

1. Graff, M., et al., *Genome-wide analysis of BMI in adolescents and young adults reveals additional* 1872 *insight into the effects of genetic loci over the life course.* Hum Mol Genet, 2013. **22**(17): p. 3597-1873 607.
