# Supplementary material for: The Influence of Age and Sex on Genetic Associations with Adult Body Size and Shape: A Large-Scale Genome-Wide Interaction Study
Source: PLoS Genet. 2015 Oct 1;11(10):e1005378. doi: 10.1371/journal.pgen.1005378 (PMC4591371; doi:10.1371/journal.pgen.1005378)
Supplement: S2 Text — (DOCX) [file pgen.1005378.s047.docx]

**Text S1:**

**Consortia members** Page 2

**Extended acknowledgements** Page 25

**Consortia members**

**The DIAGRAM Consortium (sex-specific SNP look-ups on type 2 diabetes)**

Andrew P Morris^1^, Benjamin F Voight^2,3^ , Tanya M Teslovich^4^, Teresa Ferreira^1^, Ayellet V Segrè^2,5,6^, Valgerdur Steinthorsdottir^7^, Rona J Strawbridge^8,9^, Hassan Khan^10^, Harald Grallert^11^, Anubha Mahajan^1^, Inga Prokopenko^1,12^, Hyun Min Kang^4^, Christian Dina^13^–^15^, Tonu Esko^16,17^, Ross M Fraser^18^, Stavroula Kanoni^19^, Ashish Kumar^1^, Vasiliki Lagou^1^, Claudia Langenberg^20^, Jian’an Luan^20^, Cecilia M Lindgren^1^, Martina Müller-Nurasyid^21^–^23^, Sonali Pechlivanis^24^, N William Rayner^1,12^, Laura J Scott^4^, Steven Wiltshire^1^, Loic Yengo^25,26^, Leena Kinnunen^27^, Elizabeth J Rossin^2,5,28,29^, Soumya Raychaudhuri^2,30,31^, Andrew D Johnson^32^, Antigone S Dimas^1,33,34^, Ruth J F Loos^20,35^–^37^, Sailaja Vedantam^38,39^, Han Chen^40^, Jose C Florez^5,6,38,41^, Caroline Fox^32,42^, Ching-Ti Liu^40^, Denis Rybin^43^, David J Couper^44^, Wen Hong L Kao^45^, Man Li^45^, Marilyn C Cornelis^46^, Peter Kraft^46,47^, Qi Sun^46,48^, Rob M van Dam^46,49^, Heather M Stringham^4^, Peter S Chines^50^, Krista Fischer^16^, Pierre Fontanillas^2^, Oddgeir L Holmen^51^, Sarah E Hunt^19^, Anne U Jackson^4^, Augustine Kong^7^, Robert Lawrence^52^, Julia Meyer^22^, John R B Perry^1,53^, Carl G P Platou^51,54^, Simon Potter^19^, Emil Rehnberg^55^, Neil Robertson^1,12^, Suthesh Sivapalaratnam^56^, Alena Stančáková^57^, Kathleen Stirrups^19^, Gudmar Thorleifsson^7^, Emmi Tikkanen^58,59^, Andrew R Wood^53^, Peter Almgren^60^, Mustafa Atalay^61^, Rafn Benediktsson^62,63^, Lori L Bonnycastle^50^, Noël Burtt^2^, Jason Carey^2^, Guillaume Charpentier^64^, Andrew T Crenshaw^2^, Alex S F Doney^65,66^, Mozhgan Dorkhan^60^, Sarah Edkins^19^, Valur Emilsson^67^, Elodie Eury^25^, Tom Forsen^68,69^, Karl Gertow^8,9^, Bruna Gigante^70^, George B Grant^2^, Christopher J Groves^12^, Candace Guiducci^2^, Christian Herder^71^, Astradur B Hreidarsson^63^, Jennie Hui^72^–^75^, Alan James^72,76,77^, Anna Jonsson^60^, Wolfgang Rathmann^78^, Norman Klopp^11^, Jasmina Kravic^60^, Kaarel Krjutškov^16^, Cordelia Langford^19^, Karin Leander^70^, Eero Lindholm^60^, Stéphane Lobbens^25^, Satu Männistö^59^, Ghazala Mirza^1^, Thomas W Mühleisen^79,80^, Bill Musk^72,75,77,81^, Melissa Parkin^2^, Loukianos Rallidis^82^, Jouko Saramies^83^, Bengt Sennblad^8,9^, Sonia Shah^84^, Gunnar Sigurðsson^63,67^, Angela Silveira^8,9^, Gerald Steinbach^85^, Barbara Thorand^86^, Joseph Trakalo^1^, Fabrizio Veglia^87^, Roman Wennauer^85^, Wendy Winckler^2^, Delilah Zabaneh^84^, Harry Campbell^18,88^, Cornelia van Duijn^89,90^, Andre G Uitterlinden^89^–^91^, Albert Hofman^89^, Eric Sijbrands^91^, Goncalo R Abecasis^4^, Katharine R Owen^12,92^, Eleftheria Zeggini^19^, Mieke D Trip^56^, Nita G Forouhi^20^, Ann-Christine Syvänen^93^, Johan G Eriksson^59,68,94,95^, Leena Peltonen^139^, Markus M Nöthen^79,80^, Beverley Balkau^96,97^, Colin N A Palmer^65,66^, Valeriya Lyssenko^60^, Tiinamaija Tuomi^95,98^, Bo Isomaa^95,99^, David J Hunter^46^–^48^, Lu Qi^46,48^, Alan R Shuldiner^101,103^, Michael Roden^71,104^, Ines Barroso^19,105,106^, Tom Wilsgaard^107^, John Beilby^72^–^74^, Kees Hovingh^56^, Jackie F Price^18^, James F Wilson^18,88^, Rainer Rauramaa^108,109^, Timo A Lakka^61,108^, Lars Lind^110^, George Dedoussis^111^, Inger Njølstad^107^, Nancy L Pedersen^55^, Kay-Tee Khaw^10^, Nicholas J Wareham^20^, Sirkka M Keinanen-Kiukaanniemi^112,113^, Timo E Saaristo^114,115^, Eeva Korpi-Hyövälti^116^, Juha Saltevo^117^, Markku Laakso^57^, Johanna Kuusisto^57^, Andres Metspalu^16,17^, Francis S Collins^50^, Karen L Mohlke^118^, Richard N Bergman^119^, Jaakko Tuomilehto^27,116,120,121^, Bernhard O Boehm^122^, Christian Gieger^22^, Kristian Hveem^51^, Stephane Cauchi^25^, Philippe Froguel^25,123^, Damiano Baldassarre^87,124^, Elena Tremoli^87,124^, Steve E Humphries^125^, Danish Saleheen^10,126^, John Danesh^10^, Erik Ingelsson^55^, Samuli Ripatti^19,58,59^, Veikko Salomaa^59^, Raimund Erbel^127^, Karl-Heinz Jöckel^24^, Susanne Moebus^24^, Annette Peters^86^, Thomas Illig^11,128^, Ulf de Faire^70^, Anders Hamsten^8,9^, Andrew D Morris^65,66^, Peter J Donnelly^1,129^, Timothy M Frayling^53^, Andrew T Hattersley^130^, Eric Boerwinkle^131,132^, Olle Melander^60^, Sekar Kathiresan^2,5,133^, Peter M Nilsson^60^, Panos Deloukas^19^, Unnur Thorsteinsdottir^7,62^, Leif C Groop^60^, Kari Stefansson^7,62^, Frank Hu^46,48^, James S Pankow^134^, Josée Dupuis^32,40^, James B Meigs^6,135^, David Altshuler^2,5,6,136,138,^, Michael Boehnke^4^ & Mark I McCarthy^1,12,92^

1. Wellcome Trust Centre for Human Genetics, University of Oxford, Oxford, UK.

2. Broad Institute of Harvard and Massachusetts Institute of Technology (MIT), Cambridge, Massachusetts, USA.

3. Department of Pharmacology, University of Pennsylvania–Perelman School of Medicine, Philadelphia, Pennsylvania, USA.

4. Department of Biostatistics, University of Michigan, Ann Arbor, Michigan, USA.

5. Center for Human Genetic Research, Massachusetts General Hospital, Boston, Massachusetts, USA.

6. Department of Medicine, Harvard Medical School, Boston, Massachusetts, USA.

7. deCODE Genetics, Reykjavik, Iceland.

8. Atherosclerosis Research Unit, Department of Medicine Solna, Karolinska Institutet, Stockholm, Sweden.

9. Center for Molecular Medicine, Karolinska University Hospital Solna, Stockholm, Sweden.

10. Department of Public Health and Primary Care, University of Cambridge, Cambridge, UK.

11. Research Unit of Molecular Epidemiology, Helmholtz Zentrum Muenchen, Neuherberg, Germany.

12. Oxford Centre for Diabetes, Endocrinology and Metabolism, University of Oxford, Oxford, UK.

13. Institut National de la Santé et de la Recherche Médicale (INSERM) Unité Mixte de Recherche (UMR) 1087, Nantes, France.

14. Centre National de la Recherche Scientifique (CNRS) UMR 6291, Nantes, France.

15. Department of Biology, Medicine and Health, Nantes University, Nantes, France.

16. Estonian Genome Center, University of Tartu, Tartu, Estonia.

17. Institute of Molecular and Cell Biology, University of Tartu, Tartu, Estonia.

18. Centre for Population Health Sciences, University of Edinburgh, Edinburgh, UK.

19. Wellcome Trust Sanger Institute, Hinxton, Cambridge, UK.

20. Medical Research Council (MRC) Epidemiology Unit, Institute of Metabolic Science, Addenbrooke’s Hospital, Cambridge, UK.

21. Institute of Medical Informatics, Biometry and Epidemiology, Ludwig-Maximilians-Universität, Munich, Germany.

22. Institute of Genetic Epidemiology, Helmholtz Zentrum Muenchen, Neuherberg, Germany.

23. Department of Medicine I, University Hospital Grosshadern, Ludwig-Maximilians-Universität, Munich, Germany.

24. Institute for Medical Informatics, Biometry and Epidemiology, University Hospital of Essen, University Duisburg-Essen, Essen, Germany.

25. CNRS UMR 8199, Institute of Biology and Lille 2 University, Pasteur Institute, Lille, France.

26. Laboratory of Mathematics, CNRS UMR 8524, University Lille 1, Model for Data Analysis and Learning (MODAL) Team, Institut National de Recherche en Informatique et en Automatique (INRIA) Lille Nord-Europe, Lille, France.

27. Diabetes Prevention Unit, National Institute for Health and Welfare, Helsinki, Finland.

28. Health Science and Technology MD Program, Harvard University and Massachusetts Institute of Technology, Boston, Massachusetts, USA.

29. Harvard Biological and Biomedical Sciences Program, Harvard University, Boston, Massachusetts, USA.

30. Division of Rheumatology, Immunology and Allergy, Brigham and Women’s Hospital, Harvard Medical School, Boston, Massachusetts, USA.

31. Partners Center for Personalized Genomic Medicine, Boston, Massachusetts, USA.

32. National Heart, Lung, and Blood Institute’s Framingham Heart Study, Framingham, Massachusetts, USA.

33. Department of Genetic Medicine and Development, University of Geneva Medical School, Geneva, Switzerland.

34. Biomedical Sciences Research Center Al Fleming, Vari, Greece.

35. Charles R Bronfman Institute for Personalized Medicine, Mount Sinai School of Medicine, New York, New York, USA.

36. Child Health and Development Institute, Mount Sinai School of Medicine, New York, New York, USA.

37. Department of Preventive Medicine, Mount Sinai School of Medicine, New York, New York, USA.

38. Program in Medical and Population Genetics, Broad Institute of Harvard and MIT, Cambridge, Massachusetts, USA.

39. Division of Genetics and Endocrinology, Children’s Hospital, Boston, Massachusetts, USA.

40. Department of Biostatistics, Boston University School of Public Health, Boston, Massachusetts, USA.

41. Diabetes Research Center, Diabetes Unit, Massachusetts General Hospital, Boston, Massachusetts, USA.

42. Division of Endocrinology and Metabolism, Brigham and Women’s Hospital and Harvard Medical School, Boston, Massachusetts, USA.

43. Boston University Data Coordinating Center, Boston, Massachusetts, USA.

44. Collaborative Studies Coordinating Center, Department of Biostatistics, University of North Carolina at Chapel Hill, Chapel Hill, North Carolina, USA.

45. Department of Epidemiology, Johns Hopkins Bloomberg School of Public Health, Baltimore, Maryland, USA.

46. Department of Nutrition and Epidemiology, Harvard School of Public Health, Boston, Massachusetts, USA.

47. Program in Molecular and Genetic Epidemiology, Harvard School of Public Health, Boston, Massachusetts, USA.

48. Channing Laboratory, Department of Medicine, Brigham and Women’s Hospital and Harvard Medical School, Boston, Massachusetts, USA.

49. Saw Swee Hock School of Public Health, National University of Singapore, Singapore.

50. National Human Genome Research Institute, US National Institutes of Health, Bethesda, Maryland, USA.

51. Nord-Trondelag Health Study (HUNT) Research Centre, Department of Public Health and General Practice, Norwegian University of Science and Technology, Levanger, Norway.

52. Centre for Genetic Epidemiology and Biostatistics, The University of Western Australia, Nedlands, Western Australia, Australia.

53. Genetics of Complex Traits, Institute of Biomedical and Clinical Science, Peninsula Medical School, University of Exeter, Exeter, UK.

54. Department of Internal Medicine, Levanger Hospital, Nord-Trøndelag Health Trust, Levanger, Norway.

55. Department of Medical Epidemiology and Biostatistics, Karolinska Institutet, Stockholm, Sweden.

56. Department of Vascular Medicine, Academic Medical Center, University of Amsterdam, Amsterdam, The Netherlands.

57. Department of Medicine, University of Eastern Finland and Kuopio University Hospital, Kuopio, Finland.

58. Institute for Molecular Medicine Finland (FIMM), Helsinki, Finland.

59. Department of Chronic Disease Prevention, National Institute for Health and Welfare, Helsinki, Finland.

60. Department of Clinical Science Malmö, Lund University Diabetes Centre, Scania University Hospital, Lund University, Malmö, Sweden.

61. Institute of Biomedicine, Physiology, University of Eastern Finland, Kuopio, Finland.

62. Faculty of Medicine, University of Iceland, Reykjavík, Iceland.

63. Department of Endocrinology and Metabolism, Landspitali University Hospital, Reykjavík, Iceland.

64. Endocrinology-Diabetology Unit, Corbeil-Essonnes Hospital, Corbeil-Essonnes, France.

65. Diabetes Research Centre, Biomedical Research Institute, University of Dundee, Ninewells Hospital, Dundee, UK.

66. Pharmacogenomics Centre, Biomedical Research Institute, University of Dundee, Ninewells Hospital, Dundee, UK.

67. Icelandic Heart Association, Kopavogur, Iceland.

68. Department of General Practice and Primary Health Care, University of Helsinki, Helsinki, Finland.

69. Vaasa Health Care Centre, Vaasa, Finland.

70. Division of Cardiovascular Epidemiology, Institute of Environmental Medicine, Karolinska Institutet, Stockholm, Sweden.

71. Institute for Clinical Diabetology, German Diabetes Center, Leibniz Center for Diabetes Research at Heinrich Heine University Düsseldorf, Düsseldorf, Germany.

72. Busselton Population Medical Research Institute, Sir Charles Gairdner Hospital, Nedlands, Western Australia, Australia.

73. PathWest Laboratory Medicine of Western Australia, Queen Elizabeth II Medical Centre, Nedlands, Western Australia, Australia.

74. School of Pathology and Laboratory Medicine, The University of Western Australia, Nedlands, Western Australia, Australia.

75. School of Population Health, The University of Western Australia, Nedlands, Western Australia, Australia.

76. Department of Pulmonary Physiology and Sleep Medicine, West Australian Sleep Disorders Research Institute, Queen Elizabeth II Medical Centre, Nedlands, Western Australia, Australia.

77. School of Medicine and Pharmacology, University of Western Australia, Nedlands, Western Australia, Australia.

78. Institute of Biometrics and Epidemiology, German Diabetes Center, Leibniz Center for Diabetes Research at Heinrich Heine University Düsseldorf, Düsseldorf, Germany.

79. Institute of Human Genetics, University of Bonn, Bonn, Germany.

80. Department of Genomics, Life & Brain Center, University of Bonn, Bonn, Germany.

81. Respiratory Medicine, Sir Charles Gairdner Hospital, Nedlands, Western Australia, Australia.

82. Department of Cardiology, University General Hospital Attikon, Athens, Greece.

83. South Karelia Central Hospital, Lappeenranta, Finland.

84. Department of Genetics, Evolution and Environment, University College London (UCL) Genetics Institute, University College London, London, UK.

85. Department of Clinical Chemistry and Central Laboratory, University of Ulm, Ulm, Germany.

86. Institute of Epidemiology II, Helmholtz Zentrum Muenchen, Neuherberg, Germany.

87. Centro Cardiologico Monzino, IRCCS, Milan, Italy.

88. MRC Institute of Genetics and Molecular Medicine at the University of Edinburgh, Western General Hospital, Edinburgh, UK.

89. Department of Epidemiology, Erasmus University Medical Center, Rotterdam, The Netherlands.

90. Netherland Genomics Initiative, Netherlands Consortium for Healthy Ageing and Centre for Medical Systems Biology, Rotterdam, The Netherlands.

91. Department of Internal Medicine, Erasmus University Medical Center, Rotterdam, The Netherlands.

92. Oxford National Institute for Health Research Biomedical Research Centre, Churchill Hospital, Oxford, UK.

93. Molecular Medicine, Department of Medical Sciences, Uppsala University, Uppsala, Sweden.

94. Unit of General Practice, Helsinki University General Hospital, Helsinki, Finland.

95. Folkhälsan Research Center, Helsinki, Finland.

96. INSERM CESP U1018, Villejuif, France.

97. University Paris Sud 11, UMRS 1018, Villejuif, France.

98. Department of Medicine, Helsinki University Hospital, University of Helsinki, Helsinki, Finland.

99. Department of Social Services and Health Care, Jakobstad, Finland.

101. Division of Endocrinology, Diabetes and Nutrition, University of Maryland School of Medicine, Baltimore, Maryland, USA.

102. Geriatric Research Education and Clinical Center, Baltimore Veterans Administration Medical Center, Baltimore, Maryland, USA.

103. Program in Personalized and Genomic Medicine, University of Maryland School of Medicine, Baltimore, Maryland, USA.

104. Department of Medicine/Metabolic Diseases, Heinrich Heine University Düsseldorf, Düsseldorf, Germany.

105. University of Cambridge Metabolic Research Laboratories, Institute of Metabolic Science, Addenbrooke’s Hospital, Cambridge, UK.

106. National Institute for Health Research (NIHR) Cambridge Biomedical Research Centre, Institute of Metabolic Science, Addenbrooke’s Hospital, Cambridge, UK.

107. Department of Community Medicine, Faculty of Health Sciences, University of Tromsø, Tromsø, Norway.

108. Kuopio Research Institute of Exercise Medicine, Kuopio, Finland.

109. Department of Clinical Physiology and Nuclear Medicine, Kuopio University Hospital, Kuopio, Finland.

110. Department of Medical Sciences, Uppsala University, Akademiska Sjukhuset, Uppsala, Sweden.

111. Department of Dietetics-Nutrition, Harokopio University, Athens, Greece.

112. Faculty of Medicine, Institute of Health Sciences, University of Oulu, Oulu, Finland.

113. Unit of General Practice, Oulu University Hospital, Oulu, Finland.

114. Finnish Diabetes Association, Tampere, Finland.

115. Pirkanmaa Hospital District, Tampere, Finland.

116. Department of Internal Medicine, South Ostrobothnia Central Hospital, Seinäjoki, Finland.

117. Department of Medicine, Central Finland Central Hospital, Jyväskylä, Finland.

118. Department of Genetics, University of North Carolina at Chapel Hill, Chapel Hill, North Carolina, USA.

119. Diabetes and Obesity Research Institute, Cedars-Sinai Medical Center, Los Angeles, California, USA.

120. Red RECAVA Grupo RD06/0014/0015, Hospital Universitario La Paz, Madrid, Spain.

121. Centre for Vascular Prevention, Danube-University Krems, Krems, Austria.

122. Division of Endocrinology and Diabetes, Department of Internal Medicine, University Medical Centre Ulm, Ulm, Germany.

123. Genomic Medicine, Imperial College London, Hammersmith Hospital, London, UK.

124. Department of Pharmacological Sciences, University of Milan, Milan, Italy.

125. Institute of Cardiovascular Science, University College London, London, UK.

126. Center for Non-Communicable Diseases Pakistan, Karachi, Pakistan.

127. Clinic of Cardiology, West German Heart Centre, University Hospital of Essen, University Duisburg-Essen, Essen, Germany.

128. Hannover Unified Biobank, Hannover Medical School, Hannover, Germany.

129. Department of Statistics, University of Oxford, Oxford, UK.

130. Diabetes Genetics, Institute of Biomedical and Clinical Science, Peninsula Medical School, University of Exeter, Exeter, UK.

131. Human Genetics Center, University of Texas Health Science Center at Houston, Houston, Texas, USA.

132. Human Genome Sequencing Center at Baylor College of Medicine, Houston, Texas, USA.

133. Cardiovascular Research Center, Massachusetts General Hospital, Boston, Massachusetts, USA.

134. Division of Epidemiology and Community Health, University of Minnesota, Minneapolis, Minnesota, USA.

135. General Medicine Division, Massachusetts General Hospital, Boston, Massachusetts, USA.

136. Department of Genetics, Harvard Medical School, Boston, Massachusetts, USA.

137. Department of Molecular Biology, Harvard Medical School, Boston, Massachusetts, USA.

138. Diabetes Unit, Massachusetts General Hospital, Boston, Massachusetts, USA.

**The GLGC Consortium (sex-specific SNP look-ups on lipid traits)**

Tanya M. Teslovich^1^ , Kiran Musunuru^2,3,4,5,6^, Albert V. Smith^7,8^, Andrew C. Edmondson^9,10^, Ioannis M. Stylianou^10^, Masahiro Koseki^11^, James P. Pirruccello^2,5,6^, Samuli Ripatti^12,13^, Daniel I. Chasman^4,14^, Cristen J. Willer^1^, Christopher T. Johansen^15^, Sigrid W. Fouchier^16^, Aaron Isaacs^17^, Gina M. Peloso^18,19^, Maja Barbalic^20^, Sally L. Ricketts^21^, Joshua C. Bis^22^, Yurii S. Aulchenko^17^, Gudmar Thorleifsson^23^, Mary F. Feitosa^24^, John Chambers^25^, Marju Orho-Melander^26^, Olle Melander^26^, Toby Johnson^27^, Xiaohui Li^28^, Xiuqing Guo^28^, Mingyao Li^9,10^, Yoon Shin Cho^29^, Min Jin Go^29^, Young Jin Kim^29^, Jong-Young Lee^29^, Taesung Park^30,31^, Kyunga Kim^32^, Xueling Sim^33^, Rick Twee- Hee Ong^34^, Damien C. Croteau-Chonka^35^, Leslie A. Lange^35^, Joshua D. Smith^36^, Kijoung Song^37^, Jing Hua Zhao^38^, Xin Yuan^37^, Jian'an Luan^38^, Claudia Lamina^39^, Andreas Ziegler^40^, Weihua Zhang^25^, Robert Y.L. Zee^4,14^, Alan F. Wright^41^, Jacqueline C.M. Witteman^17,42^, James F. Wilson^43^, Gonneke Willemsen^44^, H-Erich Wichmann^45^, John B. Whitfield^46^, Dawn M. Waterworth^37^, Nicholas J. Wareham^38^, Gérard Waeber^47^, Peter Vollenweider^47^, Benjamin F. Voight^2,5^, Veronique Vitart^41^, Andre G. Uitterlinden^17,42,48^, Manuela Uda^49^, Jaakko Tuomilehto^50^, John R. Thompson^51^, Toshiko Tanaka^52,53^, Ida Surakka^12,13^, Heather M. Stringham^1^, Tim D. Spector^54^, Nicole Soranzo^54,55^, Johannes H. Smit^56^, Juha Sinisalo^57^, Kaisa Silander^12,13^, Eric J.G. Sijbrands^17,48^, Angelo Scuteri^58^, James Scott^59^, David Schlessinger^60^, Serena Sanna^49^, Veikko Salomaa^13^, Juha Saharinen^61^, Chiara Sabatti^62^, Aimo Ruokonen^63^, Igor Rudan^43^, Lynda M. Rose^14^, Robert Roberts^64^, Mark Rieder^36^, Bruce M. Psaty^65^, Peter P. Pramstaller^66^, Irene Pichler^66^, Markus Perola^12,13^, Brenda W.J.H. Penninx^56^, Nancy L. Pedersen^67^, Cristian Pattaro^66^, Alex N. Parker^68^, Guillaume Pare^69^, Ben A. Oostra^70^, Christopher J. O'Donnell^4,19^, Markku S. Nieminen^57^, Deborah A. Nickerson^36^, Grant W. Montgomery^46^, Thomas Meitinger^71,72^, Ruth McPherson^64^, Mark I. McCarthy^73,74,75^, Wendy McArdle^76^, David Masson^11^, Nicholas G. Martin^46^, Fabio Marroni^77^, Massimo Mangino^54^, Patrik K.E. Magnusson^67^, Gavin Lucas^78^, Robert Luben^21^, Ruth J. F. Loos^38^, Maisa Lokki^38^, Guillaume Lettre^79^, Claudia Langenberg^38^, Lenore J. Launer^80^, Edward G. Lakatta^60^, Reijo Laaksonen^81^, Kirsten O. Kyvik^82^, Florian Kronenberg^39^, Inke R. König^40^, Kay-Tee Khaw^21^, Jaakko Kaprio^12,13,83^, Lee M. Kaplan^84^, Åsa Johansson^85^, Marjo-Riitta Jarvelin^86,87^, A. Cecile J.W. Janssens^17^, Erik Ingelsson^67^, Wilmar Igl^85^, G. Kees Hovingh^16^, Jouke-Jan Hottenga^44^, Albert Hofman^17,42^, Andrew A. Hicks^66^, Christian Hengstenberg^88^, Iris M. Heid^45,89^, Caroline Hayward^41^, Aki S. Havulinna^50,90^, Nicholas D. Hastie^41^, Tamara B. Harris^80^, Talin Haritunians^28^, Alistair S. Hall^91^, Ulf Gyllensten^85^, Candace Guiducci^5^, Leif C. Groop^26,92^, Elena Gonzalez^5^, Christian Gieger^45^, Nelson B. Freimer^93^, Luigi Ferrucci^94^, Jeanette Erdmann^95^, Paul Elliott^86,96^, Kenechi G. Ejebe^5^, Angela Döring^45^, Anna F. Dominiczak^97^, Serkalem Demissie^18,19^, Panagiotis Deloukas^55^, Eco J.C. de Geus^44^, Ulf de Faire^98^, Gabriel Crawford^5^, Francis S. Collins^99^, Yii-der I. Chen^28^, Mark J. Caulfield^27^, Harry Campbell^43^, Noel P. Burtt^5^, Lori L. Bonnycastle^99^, Dorret I. Boomsma^44^, S. Matthijs Boekholdt^100^, Richard N. Bergman^101^, Inês Barroso^55^, Stefania Bandinelli^102^, Christie M. Ballantyne^103^, Themistocles L. Assimes^104^, Thomas Quertermous^104^, David Altshuler^2,4,5^, Mark Seielstad^34^, Tien Y. Wong^105^, E-Shyong Tai^106^, Alan B. Feranil^107^, Christopher W. Kuzawa^108^, Linda S. Adair^109^, Herman A. Taylor Jr.^110^, Ingrid B. Borecki^24^, Stacey B. Gabriel^5^, James G. Wilson^110^, Kari Stefansson^23^, Unnur Thorsteinsdottir^23^, Vilmundur Gudnason^7,111^, Ronald M. Krauss^112^, Karen L. Mohlke^35^, Jose M. Ordovas^113^, Patricia B. Munroe^114^, Jaspal S. Kooner^59^, Alan R. Tall^11^, Robert A. Hegele^15^, John J.P. Kastelein^16^, Eric E. Schadt^115^, Jerome I. Rotter^28^, Eric Boerwinkle^20^, David P. Strachan^116^, Vincent Mooser^37^, Hilma Holm^23^, Muredach P. Reilly^9,10^, Nilesh J Samani^61,117^, Heribert Schunkert^95^, L. Adrienne Cupples^18,19^, Manjinder S. Sandhu^21,38,55^, Paul M Ridker^4,14^, Daniel J. Rader^9,10^, Cornelia M. van Duijn^17,42^, Leena Peltonen^5,12,13,55^, Gonçalo R. Abecasis^1^, Michael Boehnke^1^ , Sekar Kathiresan^2,3,4,5^

1. Center for Statistical Genetics, Department of Biostatistics, University of Michigan, Ann Arbor, Michigan 48109, USA

2. Center for Human Genetic Research, Massachusetts General Hospital, Boston, Massachusetts 02114, USA

3. Cardiovascular Research Center, Massachusetts General Hospital, Boston, Massachusetts 02114, USA

4. Department of Medicine, Harvard Medical School, Boston, Massachusetts 02115, USA

5. Broad Institute, Cambridge, Massachusetts 02142, USA

6. Johns Hopkins University School of Medicine, Baltimore, Maryland 21287, USA

7. Icelandic Heart Association, Heart Preventive Clinic and Research Institute, Kopavogur, Iceland

8. University of Iceland, Reykjavik, Iceland

9. Cardiovascular Institute, University of Pennsylvania School of Medicine, Philadelphia, Pennsylvania 19104, USA

10. Institute for Translational Medicine and Therapeutics, University of Pennsylvania School of Medicine, Philadelphia, Pennsylvania 19104, USA

11. Division of Molecular Medicine, Department of Medicine, Columbia University, New York, New York 10032, USA

12. Institute for Molecular Medicine Finland FIMM, University of Helsinki, P.O. Box 20, FI-00014 Helsinki, Finland

13. National Institute for Health and Welfare, P.O. Box 104, FI-00251 Helsinki, Finland

14. Division of Preventive Medicine, Brigham and Women's Hospital, Boston Massachusetts 02215, USA

15. Robarts Research Institute, University of Western Ontario, London, Ontario N6A 5K8, Canada

16. Department of Vascular Medicine, Academic Medical Centre at the University of Amsterdam, Amsterdam, The Netherlands

17. Department of Epidemiology, Erasmus University Medical Center, P.O. Box 2040, 3000 CA Rotterdam, The Netherlands

18. Department of Biostatistics, Boston University School of Public Health, Boston, Massachusetts 02118, USA

19. National Heart, Lung and Blood Institute's Framingham Heart Study, Framingham, Massachusetts 01702, USA

20. Human Genetics Center, University of Texas Health Science Center at Houston, Houston, Texas 77030, USA

21. Department of Public Health and Primary Care, Strangeways Research Laboratory, University of Cambridge, Cambridge, UK

22. Cardiovascular Health Research Unit and Department of Medicine, University of Washington, Seattle, Washington, USA

23. deCODE Genetics, 101 Reykjavik, Iceland

24. Division of Statistical Genomics in the Center for Genome Sciences, Washington University School of Medicine, St. Louis, Missouri 63108, USA

25. Department of Epidemiology and Public Health, Imperial College London, London W2 1PG, UK

26. Department of Clinical Sciences, Lund University, SE 205 02, Malmö, Sweden

27. Clinical Pharmacology and Barts and the London Genome Centre, William Harvey Research Institute, Barts and the London School of Medicine, Queen Mary University of London, London EC1M 6BQ, UK

28. Medical Genetics Institute, Cedars-Sinai Medical Center, Los Angeles, California, USA

29. Center for Genome Science, National Institute of Health, Seoul, Korea

30. Interdisciplinary Program in Bioinformatics, College of Natural Science, Seoul National University, Seoul, Korea

31. Department of Statistics, College of Natural Science, Seoul National University, Seoul, Korea

32. Department of Statistics, Sookmyung Women's University, Seoul, Korea

33. Centre for Molecular Epidemiology, National University of Singapore, Singapore

34. Genome Institute of Singapore, Singapore

35. Department of Genetics, University of North Carolina, Chapel Hill, North Carolina 27599, USA

36. Department of Genome Sciences, University of Washington, Seattle, Washington 98195, USA

37. Genetics Division, GlaxoSmithKline R&D, King of Prussia, Pennsylvania, USA

38. MRC Epidemiology Unit, Institute of Metabolic Science, Addenbrooke's Hospital, Cambridge, UK

39. Division of Genetic Epidemiology, Department of Medical Genetics, Molecular and Clinical Pharmacology, Innsbruck Medical University, Schoepfstrasse 41, A-6020 Innsbruck, Austria

40. Institut für Medizinische Biometrie und Statistik, Universität zu Lübeck, Lübeck, Germany

41. MRC Human Genetics Unit, Institute of Genetics and Molecular Medicine, Edinburgh, UK

42. Netherlands Genomics Initiative (NGI)-sponsored Netherlands Consortium for Healthy Aging (NCHA) and Center of Medical Systems Biology (CMSB), The Netherlands

43. Centre for Population Health Sciences, University of Edinburgh, Edinburgh, UK

44. Department of Biological Psychology, VU University Amsterdam, Van der Boechorststraat 1, 1081 BT Amsterdam, The Netherlands

45. Institute of Epidemiology, Helmholtz Zentrum Munchen – German Research Center for Environmental Health, 85764 Neuherberg, Germany

46. Genetic Epidemiology Unit, Queensland Institute of Medical Research, PO Royal Brisbane Hospital, Queensland 4029, Australia

47. Department of Internal Medicine, Centre Hospitalier Universitaire Vaudois, Lausanne, Switzerland

48. Department of Internal Medicine, Erasmus University Medical Center, PO Box 2040, 3000 CA Rotterdam, The Netherlands

49. Istituto di Neurogenetica e Neurofarmacologia (INN), Consiglio Nazionale delle Ricerche, c/o Cittadella Universitaria di Monserrato, Monserrato, Cagliari 09042, Italy

50. Department of Chronic Disease Prevention, National Institute for Health and Welfare, Helsinki, Finland

51. Department of Health Sciences, University of Leicester, Leicester, UK

52. Clinical Research Branch, National Institute on Aging, National Institutes of Health, Baltimore, Maryland 21225, USA

53. Medstar Research Institute, Baltimore, Maryland, USA

54. Department of Twin Research and Genetic Epidemiology, King's College London, London, SE1 7EH, UK

55. Wellcome Trust Sanger Institute, Hinxton, Cambridge, UK

56. Department of Psychiatry, EMGO Institute, Neuroscience Campus Amsterdam, VU University Medical Center, Amsterdam, The Netherlands

57. Division of Cardiology, Department of Medicine, Helsinki University Central Hospital (HUCH), Helsinki, Finland

58. Unita Operativa Geriatria, Istituto Nazionale Ricovero e Cura Anziani (INRCA), Istituto Ricovero e Cura a Carattere Scientifico (IRCCS), Via Cassia 1167, 00189 Rome, Italy

59. Hammersmith Hospital, National Heart and Lung Institute, Imperial College London, London W12 0NN, UK

60. Gerontology Research Center, National Institute on Aging, 5600 Nathan Shock Drive, Baltimore, Maryland 21224, USA

61. FIMM, Institute for Molecular Medicine, Finland, Biomedicum, P.O. Box 104, 00251 Helsinki, Finland

62. Department of Human Genetics, UCLA School of Medicine, University of California, 695 Charles E. Young Drive South, Los Angeles, California 90095, USA

63. Department of Clinical Chemistry, University of Oulu, 90220 Oulu, Finland

64. The John & Jennifer Ruddy Canadian Cardiovascular Genetics Centre, University of Ottawa, Ottawa, Canada

65. Departments of Medicine, Epidemiology, and Health Services, University of Washington, Seattle, WA; Group Health Research Institute, Group Health Cooperative, Seattle, Washington, USA

66. Institute of Genetic Medicine, European Academy Bozen/Bolzano (EURAC), Viale Druso 1, 39100 Bolzano, Italy – affiliated institute of the University of Lübeck, Germany

67. Department of Medical Epidemiology and Biostatistics, Karolinska Institutet, Stockholm, 17177, Sweden

68. Amgen, Thousand Oaks, California 91320, USA

69. Genetic and Molecular Epidemiology Laboratory, McMaster University, Hamilton, Ontario L8N3Z5, Canada

70. Department of Clinical Genetics, Erasmus University Medical Center, Rotterdam, The Netherlands

71. Institut fur Humangenetik, Helmholtz Zentrum Munchen, Deutsches Forschungszentrum fur Umwelt und Gesundheit, D-85764 Neuherberg, Germany

72. Institute of Human Genetics, Klinikum rechts der Isar, Technische Universität München, 81675 Muenchen, Germany

73. Wellcome Trust Centre for Human Genetics, University of Oxford, Roosevelt Drive, Oxford OX3 7BN, UK

74. Oxford Centre for Diabetes, Endocrinology and Medicine, University of Oxford, Churchill Hospital, Oxford OX3 7LJ, UK

75. Oxford NIHR Biomedical Research Centre, Churchill Hospital, Oxford OX3 7LJ, UK

76. Avon Longitudinal Study of Parents and Children, University of Bristol, Bristol, UK

77. Institute of Applied Genomics, via Linussio 51, 33100, Udine, Italy

78. Cardiovascular Epidemiology and Genetics, Institut Municipal d'Investigacio Medica, 08003 Barcelona, Spain

79. Montreal Heart Institute (Research Center), Université de Montréal, Montréal, Québec, Canada

80. Laboratory of Epidemiology, Demography, and Biometry, National Institute of Aging, National Institutes of Health, Bethesda, Maryland 20892, USA

81. Science Center, Tampere University Hospital, Tampere, Finland

82. Institute of Regional Health Research and the Danish Twin Registry, Institute of Public Health, University of Southern Denmark, J. B. Winsløws Vej 9B, DK-5000, Odense, Denmark

83. Faculty of Medicine, Department of Public Health, University of Helsinki, Helsinki, P.O. Box 41, FIN-00014, Finland

84. Massachusetts General Hospital Weight Center, Boston, Massachusetts 02114, USA

85. Department of Genetics and Pathology, Rudbeck Laboratory, University of Uppsala, Uppsala, Sweden

86. Department of Epidemiology & Biostatistics, Imperial College London, St Mary's Campus, Norfolk Place, London W2 1PG, UK

87. Department of Public Health Science and General Practice, University of Oulu, Finland

88. Klinik und Poliklinik für Innere Medizin II, Universität Regensburg, Regensburg, Germany

89. Department of Epidemiology and Preventive Medicine Regensburg University Medical Center Franz-Josef-Strauss-Allee 11 93053 Regensburg, Germany

90. Department of Biomedical Engineering and Computational Science, Helsinki University of Technology, Espoo, Finland

91. LIGHT Research Institute, Faculty of Medicine and Health, University of Leeds, Leeds, UK

92. Department of Medicine, Helsinki University Hospital, Helsinki 00029, Finland

93. Department of Psychiatry, Center for Neurobehavioral Genetics, The Jane and Terry Semel Institute for Neuroscience and Human Behavior, David Geffen School of Medicine, University of California, Los Angeles, California 90095, USA

94. Clinical Research Branch, National Institute on Aging, National Institutes of Health, Baltimore, Maryland 21225, USA

95. Medizinische Klinik II, Universität zu Lübeck, Lübeck, Germany

96. MRC-HPA Centre for Environment and Health, Imperial College London, UK

97. BHF Glasgow Cardiovascular Research Centre, University of Glasgow, 126 University Place, Glasgow, G12 8TA, UK

98. Division of Cardiovascular Epidemiology, Institute of Environmental Medicine, Karolinska Institutet, S-171 77 Stockholm, Sweden

99. National Human Genome Research Institute, National Institutes of Health, Bethesda, Maryland 20892, USA

100. Departments of Vascular Medicine & Cardiology, Academic Medical Center, Amsterdam, The Netherlands

101. Department of Physiology and Biophysics, University of Southern California, Los Angeles, California 90033, USA

102. Geriatric Unit, Azienda Sanitaria Firenze (ASF), Florence, Italy

103. Department of Medicine, Baylor College of Medicine, Houston, Texas, USA

104. Department of Medicine, Stanford University School of Medicine, Stanford, California 94305, USA

105. Singapore Eye Research Institute, National University of Singapore, Singapore

106. Departments of Medicine/Epidemiology and Public Health, Yong Loo Lin School of Medicine, National University of Singapore, Singapore

107. Office of Population Studies Foundation, University of San Carlos, Cebu City 6000, Philippines

108. Department of Anthropology, Northwestern University, Evanston, Illinois 60208, USA

109. Department of Nutrition, Carolina Population Center, University of North Carolina, Chapel Hill, North Carolina 27516, USA

110. Department of Medicine, University of Mississippi Medical Center, Jackson, Mississippi, USA

111. University of Iceland, Reykjavik, Iceland

112. Children's Hospital Oakland Research Institute, Oakland, California 94609, USA

113. Department of Cardiovascular Epidemiology and Population Genetics, Centro Nacional de Investigaciones Cardiovasculares, Madrid, Spain, and Nutrition and Genomics Laboratory, Jean Mayer United States Department of Agriculture Human Nutrition Research Center on Aging at Tufts University, Boston, Massachusetts 02111, USA

114. Clinical Pharmacology and Barts and The London Genome Centre, William Harvey Research Institute, Barts and The London School of Medicine and Dentistry, Queen Mary University of London, Charterhouse Square, London EC1M 6BQ, UK

115. Sage Bionetworks, Seattle, Washington 98109, USA

116. Division of Community Health Sciences, St George's, University of London, London, UK

117. Department of Cardiovascular Sciences, University of Leicester, Glenfield Hospital, Leicester, UK; Leicester NIHR Biomedical Research Unit in Cardiovascular Disease, Glenfield Hospital, Leicester, LE3 9QP, UK

**The ICBP Consortium (sex-specific SNP look-ups on blood pressure traits)**

*Steering Committee*

Gonçalo Abecasis, Murielle Bochud, Mark Caulfield (co-chair), Aravinda Chakravarti, Dan Chasman, Georg Ehret (co-chair), Paul Elliott, Andrew Johnson, Louise Johnson, Martin Larson, Daniel Levy (co-chair), Patricia Munroe (co-chair), Christopher Newton-Cheh (co-chair), Paul O'Reilly, Walter Palmas, Bruce Psaty, Kenneth Rice, Albert Smith, Harold Snider, Martin Tobin, Cornelia Van Duijn, Germaine Verwoert.

*Authors*

Georg B. Ehret^1,2,3^, Patricia B. Munroe^4^, Kenneth M. Rice^5^, Murielle Bochud^2^, Andrew D. Johnson^6,7^, Daniel I. Chasman^8,9^, Albert V. Smith^10,11^, Martin D. Tobin^12^, Germaine C. Verwoert^13,14,15^, Shih-Jen Hwang^6,16,7^, Vasyl Pihur^1^, Peter Vollenweider^17^, Paul F. O'Reilly^18^, Najaf Amin^13^, Jennifer L Bragg-Gresham^19^, Alexander Teumer^20^, Nicole L. Glazer^21^, Lenore Launer^22^, Jing Hua Zhao^23^, Yurii Aulchenko^13^, Simon Heath^24^, Siim Sõber^25^, Afshin Parsa^26^, Jian'an Luan^23^, Pankaj Arora^27^, Abbas Dehghan^13,14,15^, Feng Zhang^28^, Gavin Lucas^29^, Andrew A. Hicks^30^, Anne U. Jackson^31^, John F Peden^32^, Toshiko Tanaka^33^, Sarah H. Wild^34^, Igor Rudan^35,36^, Wilmar Igl^37^, Yuri Milaneschi^33^, Alex N. Parker^38^, Cristiano Fava^39,40^, John C. Chambers^18,41^, Ervin R. Fox^42^, Meena Kumari^43^, Min Jin Go^44^, Pim van der Harst^45^, Wen Hong Linda Kao^46^, Marketa Sjögren^39^, D. G. Vinay^47^, Myriam Alexander^48^, Yasuharu Tabara^49^, Sue Shaw-Hawkins^4^, Peter H. Whincup^50^, Yongmei Liu^51^, Gang Shi^52^, Johanna Kuusisto^53^, Bamidele Tayo^54^, Mark Seielstad^55,56^, Xueling Sim^57^, Khanh-Dung Hoang Nguyen^1^, Terho Lehtimäki^58^, Giuseppe Matullo^59,60^, Ying Wu^61^, Tom R. Gaunt^62^, N. Charlotte Onland-Moret^63,64^, Matthew N. Cooper^65^, Carl G.P. Platou^66^, Elin Org^25^, Rebecca Hardy^67^, Santosh Dahgam^68^, Jutta Palmen^69^, Veronique Vitart^70^, Peter S. Braund^71,72^, Tatiana Kuznetsova^73^, Cuno S.P.M. Uiterwaal^63^, Adebowale Adeyemo^74^, Walter Palmas^75^, Harry Campbell^35^, Barbara Ludwig^76^, Maciej Tomaszewski^71,72^, Ioanna Tzoulaki^77,78^, Nicholette D. Palmer^79^, Thor Aspelund^10,11^, Melissa Garcia^22^, Yen-Pei C. Chang^26^, Jeffrey R. O'Connell^26^, Nanette I. Steinle^26^, Diederick E. Grobbee^63^, Dan E. Arking^1^, Sharon L. Kardia^81^, Alanna C. Morrison^82^, Dena Hernandez^83^, Samer Najjar^84,85^, Wendy L. McArdle^86^, David Hadley^50,87^, Morris J. Brown^88^, John M. Connell^89^, Aroon D. Hingorani^90^, Ian N.M. Day^62^, Debbie A. Lawlor^62^, John P. Beilby^91,92^, Robert W. Lawrence^65^, Robert Clarke^93^, Rory Collins^93^, Jemma C Hopewell^93^, Halit Ongen^32^, Albert W. Dreisbach^42^, Yali Li^94^, J H. Young^95^, Joshua C. Bis^21^, Mika Kähönen^96^, Jorma Viikari^97^, Linda S. Adair^98^, Nanette R. Lee^99^, Ming-Huei Chen^100^, Matthias Olden^101,102^, Cristian Pattaro^30^, Judith A. Hoffman Bolton^103^, Anna Köttgen^104,103^, Sven Bergmann^105,106^, Vincent Mooser^107^, Nish Chaturvedi^108^, Timothy M. Frayling^109^, Muhammad Islam^110^, Tazeen H. Jafar^110^, Jeanette Erdmann^111^, Smita R. Kulkarni^112^, Stefan R. Bornstein^76^, Jürgen Grässler^76^, Leif Groop^113,114^, Benjamin F. Voight^115^, Johannes Kettunen^116,126^, Philip Howard^117^, Andrew Taylor^43^, Simonetta Guarrera^60^, Fulvio Ricceri^59,60^, Valur Emilsson^118^, Andrew Plump^118^, Inês Barroso^119,120^, Kay-Tee Khaw^48^, Alan B. Weder^121^, Steven C. Hunt^122^, Yan V. Sun^81^, Richard N. Bergman^123^, Francis S. Collins^124^, Lori L. Bonnycastle^124^, Laura J. Scott^31^, Heather M. Stringham^31^, Leena Peltonen^119,125,126,127^, Markus Perola^125^, Erkki Vartiainen^125^, Stefan-Martin Brand^128,129^, Jan A. Staessen^73^, Thomas J. Wang^6,130^, Paul R. Burton^12,72^, Maria Soler Artigas^12^, Yanbin Dong^131^, Harold Snieder^132,131^, Xiaoling Wang^131^, Haidong Zhu^131^, Kurt K. Lohman^133^, Megan E. Rudock^51^, Susan R Heckbert^134,135^, Nicholas L Smith^134,136,135^, Kerri L Wiggins^137^, Ayo Doumatey^74^, Daniel Shriner^74^, Gudrun Veldre^25,138^, Margus Viigimaa^139,140^, Sanjay Kinra^141^, Dorairajan Prabhakaran^142^, Vikal Tripathy^142^, Carl D. Langefeld^79^, Annika Rosengren^143^, Dag S. Thelle^144^, Anna Maria Corsi^145^, Andrew Singleton^83^, Terrence Forrester^146^, Gina Hilton^1^, Colin A. McKenzie^146^, Tunde Salako^147^, Naoharu Iwai^148^, Yoshikuni Kita^149^, Toshio Ogihara^150^, Takayoshi Ohkubo^149,151^, Tomonori Okamura^148^, Hirotsugu Ueshima^152^, Satoshi Umemura^153^, Susana Eyheramendy^154^, Thomas Meitinger^155,156^, H.-Erich Wichmann^157,158,159^, Yoon Shin Cho^44^, Hyung-Lae Kim^44^, Jong-Young Lee^44^, James Scott^160^, Joban S. Sehmi^160,41^, Weihua Zhang^18^, Bo Hedblad^39^, Peter Nilsson^39^, George Davey Smith^62^, Andrew Wong^67^, Narisu Narisu^124^, Alena Stančáková^53^, Leslie J. Raffel^161^, Jie Yao^161^, Sekar Kathiresan^162,27^, Chris O'Donnell^163,27,9^, Stephen M. Schwartz^134^, M. Arfan Ikram^13,15^, W. T. Longstreth Jr.^164^, Thomas H. Mosley^165^, Sudha Seshadri^166^, Nick R.G. Shrine^12^, Louise V. Wain^12^, Mario A. Morken^124^, Amy J. Swift^124^, Jaana Laitinen^167^, Inga Prokopenko^51,168^, Paavo Zitting^169^, Jackie A. Cooper^69^, Steve E. Humphries^69^, John Danesh^48^, Asif Rasheed^170^, Anuj Goel^32^, Anders Hamsten^171^, Hugh Watkins^32^, Stephan J.L. Bakker^172^, Wiek H. van Gilst^45^, Charles S. Janipalli^47^, K. Radha Mani^47^, Chittaranjan S. Yajnik^112^, Albert Hofman^13^, Francesco U.S. Mattace-Raso^13,14^, Ben A. Oostra^173^, Ayse Demirkan^13^, Aaron Isaacs^13^, Fernando Rivadeneira^13,14^, Edward G Lakatta^174^, Marco Orru^175,176^, Angelo Scuteri^174^, Mika Ala-Korpela^177,178,179^, Antti J Kangas^177^, Leo-Pekka Lyytikäinen^58^, Pasi Soininen^177,178^, Taru Tukiainen^180,181,177^, Peter Würtz^177,18,180^, Rick Twee-Hee Ong^56,57,182^, Marcus Dörr^183^, Heyo K. Kroemer^184^, Uwe Völker^20^, Henry Völzke^185^, Pilar Galan^186^, Serge Hercberg^186^, Mark Lathrop^24^, Diana Zelenika^24^, Panos Deloukas^119^, Massimo Mangino^28^, Tim D. Spector^28^, Guangju Zhai^28^, James F. Meschia^187^, Michael A. Nalls^83^, Pankaj Sharma^188^, Janos Terzic^189^, M. J. Kranthi Kumar^47^, Matthew Denniff^71^, Ewa Zukowska-Szczechowska^190^, Lynne E. Wagenknecht^79^, F. Gerald R. Fowkes^191^, Fadi J. Charchar^192^, Peter E.H. Schwarz^193^, Caroline Hayward^70^, Xiuqing Guo^161^, Charles Rotimi^74^, Michiel L. Bots^63^, Eva Brand^194^, Nilesh J. Samani^71,72^, Ozren Polasek^195^, Philippa J. Talmud^69^, Fredrik Nyberg^68,196^, Diana Kuh^67^, Maris Laan^25^, Kristian Hveem^66^, Lyle J. Palmer^197,198^, Yvonne T. van der Schouw^63^, Juan P. Casas^199^, Karen L. Mohlke^61^, Paolo Vineis^200,60^, Olli Raitakari^201^, Santhi K. Ganesh^202^, Tien Y. Wong^203,204^, E Shyong Tai^205,57,206^, Richard S. Cooper^54^, Markku Laakso^53^, Dabeeru C. Rao^207^, Tamara B. Harris^22^, Richard W. Morris^208^, Anna F. Dominiczak^209^, Mika Kivimaki^210^, Michael G. Marmot^210^, Tetsuro Miki^49^, Danish Saleheen^170,48^, Giriraj R. Chandak^47^, Josef Coresh^211^, Gerjan Navis^212^, Veikko Salomaa^125^, Bok-Ghee Han^44^, Xiaofeng Zhu^94^, Jaspal S. Kooner^160,41^, Olle Melander^39^, Paul M Ridker^8,213,9^, Stefania Bandinelli^214^, Ulf B. Gyllensten^37^, Alan F. Wright^70^, James F. Wilson^34^, Luigi Ferrucci^33^, Martin Farrall^32^, Jaakko Tuomilehto^215,216,217,218^, Peter P. Pramstaller^30,219^, Roberto Elosua^29,220^, Nicole Soranzo^119,28^, Eric J.G. Sijbrands^13,14^, David Altshuler^221,115^, Ruth J.F. Loos^23^, Alan R. Shuldiner^26,222^, Christian Gieger^157^, Pierre Meneton^223^, Andre G. Uitterlinden^13,14,15^, Nicholas J. Wareham^23^, Vilmundur Gudnason^10,11^, Jerome I. Rotter^161^, Rainer Rettig^224^, Manuela Uda^175^, David P. Strachan^50^, Jacqueline C.M. Witteman^13,15^, Anna-Liisa Hartikainen^225^, Jacques S. Beckmann^105,226^, Eric Boerwinkle^227^, Ramachandran S. Vasan^6,228^, Michael Boehnke^31^, Martin G. Larson^6,229^, Marjo-Riitta Järvelin^18,230,231,232,233^, Bruce M. Psaty^21,135*^, Gonçalo R Abecasis^19*^, Aravinda Chakravarti^1^, Paul Elliott^18,233*^, Cornelia M. van Duijn^13,234*^, Christopher Newton-Cheh^27,115^, Daniel Levy^6,16,7^, Mark J. Caulfield^4^, Toby Johnson^4^

*Affiliations*

1. Center for Complex Disease Genomics, McKusick-Nathans Institute of Genetic Medicine, Johns Hopkins University School of Medicine, Baltimore, MD 21205, USA

2. Institute of Social and Preventive Medicine (IUMSP), Centre Hospitalier Universitaire Vaudois and University of Lausanne, Bugnon 17, 1005 Lausanne, Switzerland

3. Cardiology, Department of Specialties of Internal Medicine, Geneva University Hospital, Rue Gabrielle-Perret-Gentil 4, 1211 Geneva 14, Switzerland

4. Clinical Pharmacology and The Genome Centre, William Harvey Research Institute, Barts and The London School of Medicine and Dentistry, Queen Mary University of London, London EC1M 6BQ, UK

5. Department of Biostatistics, University of Washington, Seattle, WA, USA

6. Framingham Heart Study, Framingham, MA, USA

7. National Heart Lung, and Blood Institute, Bethesda, MD, USA

8. Division of Preventive Medicine, Brigham and Women's Hospital, 900 Commonwealth Avenue East, Boston MA 02215, USA

9. Harvard Medical School, Boston, MA, USA

10. Icelandic Heart Association, Kopavogur, Iceland

11. University of Iceland, Reykajvik, Iceland

12. Department of Health Sciences, University of Leicester, University Rd, Leicester LE1 7RH, UK

13. Department of Epidemiology, Erasmus Medical Center, PO Box 2040, 3000 CA, Rotterdam, The Netherlands

14. Department of Internal Medicine, Erasmus Medical Center, Rotterdam, The Netherlands

15. Netherlands Consortium for Healthy Aging (NCHA), Netherland Genome Initiative (NGI), The Netherlands

16. Center for Population Studies, National Heart Lung, and Blood Institute, Bethesda, MD, USA

17. Department of Internal Medicine, Centre Hospitalier Universitaire Vaudois, 1011 Lausanne, Switzerland

18. Department of Epidemiology and Biostatistics, School of Public Health, Imperial College London, Norfolk Place, London W2 1PG, UK

19. Center for Statistical Genetics, Department of Biostatistics, University of Michigan School of Public Health, Ann Arbor, MI 48103, USA

20. Interfaculty Institute for Genetics and Functional Genomics, Ernst-Moritz-Arndt-University Greifswald, 17487 Greifswald, Germany

21. Cardiovascular Health Research Unit, Departments of Medicine, Epidemiology and Health Services, University of Washington, Seattle, WA, USA

22. Laboratory of Epidemiology, Demography, Biometry, National Institute on Aging, National Institutes of Health, Bethesda, Maryland 20892, USA

23. MRC Epidemiology Unit, Institute of Metabolic Science, Cambridge CB2 0QQ, UK

24. Centre National de Génotypage, Commissariat à L'Energie Atomique, Institut de Génomique, Evry, France

25. Institute of Molecular and Cell Biology, University of Tartu, Riia 23, Tartu 51010, Estonia

26. University of Maryland School of Medicine, Baltimore, MD, USA, 21201, USA

27. Center for Human Genetic Research, Cardiovascular Research Center, Massachusetts General Hospital, Boston, Massachusetts, 02114, USA

28. Department of Twin Research & Genetic Epidemiology, King's College London, UK

29. Cardiovascular Epidemiology and Genetics, Institut Municipal d'Investigacio Medica, Barcelona Biomedical Research Park, 88 Doctor Aiguader, 08003 Barcelona, Spain

30. Institute of Genetic Medicine, European Academy Bozen/Bolzano (EURAC), Viale Druso 1, 39100 Bolzano, Italy - Affiliated Institute of the University of Lübeck, Germany

31. Department of Biostatistics, Center for Statistical Genetics, University of Michigan, Ann Arbor, Michigan, 48109, USA

32. Department of Cardiovascular Medicine, The Wellcome Trust Centre for Human Genetics, University of Oxford, Oxford, OX3 7BN, UK

33. Clinical Research Branch, National Institute on Aging, Baltimore MD 21250, USA

34. Centre for Population Health Sciences, University of Edinburgh, EH89AG, UK

35. Centre for Population Health Sciences and Institute of Genetics and Molecular Medicine, College of Medicine and Vet Medicine, University of Edinburgh, EH8 9AG, UK

36. Croatian Centre for Global Health, University of Split, Croatia

37. Department of Genetics and Pathology, Rudbeck Laboratory, Uppsala University, SE-751 85 Uppsala, Sweden

38. Amgen, 1 Kendall Square, Building 100, Cambridge, MA 02139, USA

39. Department of Clinical Sciences, Lund University, Malmö, Sweden

40. Department of Medicine, University of Verona, Italy

41. Ealing Hospital, London, UB1 3HJ, UK

42. Department of Medicine, University of Mississippi Medical Center, USA

43. Genetic Epidemiology Group, Epidemiology and Public Health, UCL, London, WC1E 6BT, UK

44. Center for Genome Science, National Institute of Health, Seoul, Korea

45. Department of Cardiology, University Medical Center Groningen, University of Groningen, The Netherlands

46. Departments of Epidemiology and Medicine, Johns Hopkins University, Baltimore MD, USA

47. Centre for Cellular and Molecular Biology (CCMB), Council of Scientific and Industrial Research (CSIR), Uppal Road, Hyderabad 500 007, India

48. Department of Public Health and Primary Care, University of Cambridge, CB1 8RN, UK

49. Department of Basic Medical Research and Education, and Department of Geriatric Medicine, Ehime University Graduate School of Medicine, Toon, 791-0295, Japan

50. Division of Community Health Sciences, St George's University of London, London, SW17 0RE, UK

51. Epidemiology & Prevention, Division of Public Health Sciences, Wake Forest University School of Medicine, Winston-Salem, NC 27157, USA

52. Division of Biostatistics and Department of Genetics, School of Medicine, Washington University in St. Louis, Saint Louis, Missouri 63110, USA

53. Department of Medicine, University of Eastern Finland and Kuopio University Hospital, 70210 Kuopio, Finland

54. Department of Preventive Medicine and Epidemiology, Loyola University Medical School, Maywood, IL, USA

55. Department of Laboratory Medicine & Institute of Human Genetics, University of California San Francisco, 513 Parnassus Ave. San Francisco CA 94143, USA

56. Genome Institute of Singapore, Agency for Science, Technology and Research, Singapore, 138672, Singapore

57. Centre for Molecular Epidemiology, Yong Loo Lin School of Medicine, National University of Singapore, Singapore, 117597, Singapore

58. Department of Clinical Chemistry, University of Tampere and Tampere University Hospital, Tampere, 33521, Finland

59. Department of Genetics, Biology and Biochemistry, University of Torino, Via Santena 19, 10126, Torino, Italy

60. Human Genetics Foundation (HUGEF), Via Nizza 52, 10126, Torino, Italy

61. Department of Genetics, University of North Carolina, Chapel Hill, NC, 27599, USA

62. MRC Centre for Causal Analyses in Translational Epidemiology, School of Social & Community Medicine, University of Bristol, Bristol BS8 2BN, UK

63. Julius Center for Health Sciences and Primary Care, University Medical Center Utrecht, Heidelberglaan 100, 3508 GA Utrecht, The Netherlands

64. Complex Genetics Section, Department of Medical Genetics - DBG, University Medical Center Utrecht, 3508 GA Utrecht, The Netherlands

65. Centre for Genetic Epidemiology and Biostatistics, University of Western Australia, Crawley, WA, Australia

66. HUNT Research Centre, Department of Public Health and General Practice, Norwegian University of Science and Technology, 7600 Levanger, Norway

67. MRC Unit for Lifelong Health & Ageing, London, WC1B 5JU, UK

68. Occupational and Environmental Medicine, Department of Public Health and Community Medicine, Institute of Medicine, Sahlgrenska Academy, University of Gothenburg, 40530 Gothenburg, Sweden

69. Centre for Cardiovascular Genetics, University College London, London WC1E 6JF, UK

70. MRC Human Genetics Unit and Institute of Genetics and Molecular Medicine, Edinburgh, EH2, UK

71. Department of Cardiovascular Sciences, University of Leicester, Glenfield Hospital, Leicester, LE3 9QP, UK

72. Leicester NIHR Biomedical Research Unit in Cardiovascular Disease, Glenfield Hospital, Leicester, LE3 9QP, UK

73. Studies Coordinating Centre, Division of Hypertension and Cardiac Rehabilitation, Department of Cardiovascular Diseases, University of Leuven, Campus Sint Rafaël, Kapucijnenvoer 35, Block D, Box 7001, 3000 Leuven, Belgium

74. Center for Research on Genomics and Global Health, National Human Genome Research Institute, Bethesda, MD 20892, USA

75. Columbia University, NY, USA

76. Department of Medicine III, Medical Faculty Carl Gustav Carus at the Technical University of Dresden, 01307 Dresden, Germany

77. Epidemiology and Biostatistics, School of Public Health, Imperial College, London, W2 1PG, UK

78. Clinical and Molecular Epidemiology Unit, Department of Hygiene and Epidemiology, University of Ioannina School of Medicine, Ioannina, Greece

79. Wake Forest University Health Sciences, Winston-Salem, NC 27157, USA

81. Department of Epidemiology, School of Public Health, University of Michigan, Ann Arbor, MI 48109, USA

82. Division of Epidemiology, Human Genetics and Environmental Sciences, School of Public Health, University of Texas at Houston Health Science Center, 12 Herman Pressler, Suite 453E, Houston, TX 77030, USA

83. Laboratory of Neurogenetics, National Institute on Aging, Bethesda, MD 20892, USA

84. Laboratory of Cardiovascular Science, Intramural Research Program, National Institute on Aging, NIH, Baltimore, Maryland, USA

85. Washington Hospital Center, Division of Cardiology, Washington DC, USA

86. ALSPAC Laboratory, University of Bristol, Bristol, BS8 2BN, UK

87. Pediatric Epidemiology Center, University of South Florida, Tampa, FL, USA

88. Clinical Pharmacology Unit, University of Cambridge, Addenbrookes Hospital, Hills Road, Cambridge CB2 2QQ, UK

89. University of Dundee, Ninewells Hospital &Medical School, Dundee, DD1 9SY, UK

90. Genetic Epidemiology Group, Department of Epidemiology and Public Health, UCL, London WC1E 6BT, UK

91. Pathology and Laboratory Medicine, University of Western Australia, Crawley, WA, Australia

92. Molecular Genetics, PathWest Laboratory Medicine, Nedlands, WA, Australia

93. Clinical Trial Service Unit and Epidemiological Studies Unit, University of Oxford, Oxford, OX3 7LF, UK

94. Department of Epidemiology and Biostatistics, Case Western Reserve University, 2103 Cornell Road, Cleveland, OH 44106, USA

95. Department of Medicine, Johns Hopkins University, Baltimore, USA

96. Department of Clinical Physiology, University of Tampere and Tampere University Hospital, Tampere, 33521, Finland

97. Department of Medicine, University of Turku and Turku University Hospital, Turku, 20521, Finland

98. Department of Nutrition, University of North Carolina, Chapel Hill, NC, 27599, USA

99. Office of Population Studies Foundation, University of San Carlos, Talamban, Cebu City 6000, Philippines

100. Department of Neurology and Framingham Heart Study, Boston University School of Medicine, Boston, MA, 02118, USA

101. Department of Internal Medicine II, University Medical Center Regensburg, 93053 Regensburg, Germany

102. Department of Epidemiology and Preventive Medicine, University Medical Center Regensburg, 93053 Regensburg, Germany

103. Department of Epidemiology, Johns Hopkins University, Baltimore MD, USA

104. Renal Division, University Hospital Freiburg, Germany

105. Département de Génétique Médicale, Université de Lausanne, 1015 Lausanne, Switzerland

106. Swiss Institute of Bioinformatics, 1015 Lausanne, Switzerland

107. Division of Genetics, GlaxoSmithKline, Philadelphia, Pennsylvania 19101, USA

108. International Centre for Circulatory Health, National Heart & Lung Institute, Imperial College, London, UK

109. Genetics of Complex Traits, Peninsula Medical School, University of Exeter, UK

110. Department of Community Health Sciences & Department of Medicine, Aga Khan University, Karachi, Pakistan

111. Medizinische Klinik II, Universität zu Lübeck, Lübeck, Germany

112. Diabetes Unit, KEM Hospital and Research Centre, Rasta Peth, Pune-411011, Maharashtra, India

113. Department of Clinical Sciences, Diabetes and Endocrinology Research Unit, University Hospital, Malmö, Sweden

114. Lund University, Malmö 20502, Sweden

115. Program in Medical and Population Genetics, Broad Institute of Harvard and MIT, Cambridge, Massachusetts, 02139, USA

116. Department of Chronic Disease Prevention, National Institute for Health and Welfare, FIN-00251 Helsinki, Finland

117. William Harvey Research Institute, Barts and The London School of Medicine and Dentistry, Queen Mary University of London, London EC1M 6BQ, UK

118. Merck Research Laboratory, 126 East Lincoln Avenue, Rahway, NJ 07065, USA

119. Wellcome Trust Sanger Institute, Hinxton, CB10 1SA, UK

120. University of Cambridge Metabolic Research Labs, Institute of Metabolic Science Addenbrooke's Hospital, CB2 OQQ, Cambridge, UK

121. Division of Cardiovascular Medicine, Department of Internal Medicine, University of Michigan Medical School, Ann Arbor, MI, USA

122. Cardiovascular Genetics, University of Utah School of Medicine, Salt Lake City, UT, USA

123. Department of Physiology and Biophysics, Keck School of Medicine, University of Southern California, Los Angeles, California 90033, USA

124. National Human Genome Research Institute, National Institutes of Health, Bethesda, Maryland 20892,USA

125. National Institute for Health and Welfare, 00271 Helsinki, Finland

126. FIMM, Institute for Molecular Medicine, Finland, Biomedicum, P.O. Box 104, 00251 Helsinki, Finland

127. Broad Institute, Cambridge, Massachusetts 02142, USA

128. Leibniz-Institute for Arteriosclerosis Research, Department of Molecular Genetics of Cardiovascular Disease, University of Münster, Münster, Germany

129. Medical Faculty of the Westfalian Wilhelms University Muenster, Department of Molecular Genetics of Cardiovascular Disease, University of Muenster, Muenster, Germany

130. Division of Cardiology, Massachusetts General Hospital, Boston, MA, USA

131. Georgia Prevention Institute, Department of Pediatrics, Medical College of Georgia, Augusta, GA, USA

132. Unit of Genetic Epidemiology and Bioinformatics, Department of Epidemiology, University Medical Center Groningen, University of Groningen, Groningen, The Netherlands

133. Department of Biostatical Sciences, Division of Public Health Sciences, Wake Forest University School of Medicine, Winston-Salem, NC 27157, USA

134. Department of Epidemiology, University of Washington, Seattle, WA, 98195, USA

135. Group Health Research Institute, Group Health Cooperative, Seattle, WA, USA

136. Seattle Epidemiologic Research and Information Center, Veterans Health Administration Office of Research & Development, Seattle, WA 98108, USA

137. Department of Medicine, University of Washington, 98195, USA

138. Department of Cardiology, University of Tartu, L. Puusepa 8, 51014 Tartu, Estonia

139. Tallinn University of Technology, Institute of Biomedical Engineering, Ehitajate tee 5, 19086 Tallinn, Estonia

140. Centre of Cardiology, North Estonia Medical Centre, Sütiste tee 19, 13419 Tallinn, Estonia

141. Division of Non-communicable disease Epidemiology, The London School of Hygiene and Tropical Medicine London, Keppel Street, London WC1E 7HT, UK

142. South Asia Network for Chronic Disease, Public Health Foundation of India, C-1/52, SDA, New Delhi 100016, India

143. Department of Emergency and Cardiovascular Medicine, Institute of Medicine, Sahlgrenska Academy, University of Gothenburg, 41685 Gothenburg, Sweden

144. Department of Biostatistics, Institute of Basic Medical Sciences, University of Oslo, 0317 Oslo, Norway

145. Tuscany Regional Health Agency, Florence, Italy

146. Tropical Medicine Research Institute, University of the West Indies, Mona, Kingston, Jamaica

147. University of Ibadan, Ibadan, Nigeria

148. Department of Genomic Medicine, and Department of Preventive Cardiology, National Cerebral and Cardiovascular Research Center, Suita, 565-8565, Japan

149. Department of Health Science, Shiga University of Medical Science, Otsu, 520-2192, Japan

150. Department of Geriatric Medicine, Osaka University Graduate School of Medicine, Suita, 565-0871, Japan

151. Tohoku University Graduate School of Pharmaceutical Sciences and Medicine, Sendai, 980-8578, Japan

152. Lifestyle-related Disease Prevention Center, Shiga University of Medical Science, Otsu, 520-2192, Japan

153. Department of Medical Science and Cardiorenal Medicine, Yokohama City University School of Medicine, Yokohama, 236-0004, Japan

154. Department of Statistics, Pontificia Universidad Catolica de Chile, Vicuña Mackena 4860, Santiago, Chile

155. Institute of Human Genetics, Helmholtz Zentrum Munich, German Research Centre for Environmental Health, 85764 Neuherberg, Germany

156. Institute of Human Genetics, Klinikum rechts der Isar, Technical University of Munich, 81675 Munich, Germany

157. Institute of Epidemiology, Helmholtz Zentrum Munich, German Research Centre for Environmental Health, 85764 Neuherberg, Germany

158. Chair of Epidemiology, Institute of Medical Informatics, Biometry and Epidemiology, Ludwig-Maximilians-Universität, 81377 Munich, Germany

159. Klinikum Grosshadern, 81377 Munich, Germany

160. National Heart and Lung Institute, Imperial College London, London, UK, W12 0HS, UK

161. Medical Genetics Institute, Cedars-Sinai Medical Center, Los Angeles, CA, USA

162. Medical Population Genetics, Broad Institute of Harvard and MIT, 5 Cambridge Center, Cambridge MA 02142, USA

163. National Heart, Lung and Blood Institute and its Framingham Heart Study, 73 Mount Wayte Ave., Suite #2, Framingham, MA 01702, USA

164. Department of Neurology and Medicine, University of Washington, Seattle, USA

165. Department of Medicine (Geriatrics), University of Mississippi Medical Center, Jackson, MS, USA

166. Department of Neurology, Boston University School of Medicine, USA

167. Finnish Institute of Occupational Health, Finnish Institute of Occupational Health, Aapistie 1, 90220 Oulu, Finland

168. Wellcome Trust Centre for Human Genetics, University of Oxford, UK

169. Lapland Central Hospital, Department of Physiatrics, Box 8041, 96101 Rovaniemi, Finland

170. Center for Non-Communicable Diseases Karachi, Pakistan

171. Atherosclerosis Research Unit, Department of Medicine, Karolinska Institute, Stockholm, Sweden

172. Department of Internal Medicine, University Medical Center Groningen, University of Groningen, The Netherlands

173. Department of Medical Genetics, Erasmus Medical Center, Rotterdam, The Netherlands

174. Gerontology Research Center, National Institute on Aging, Baltimore, MD 21224, USA

175. Istituto di Neurogenetica e Neurofarmacologia, Consiglio Nazionale delle Ricerche, Cittadella Universitaria di Monserrato, Monserrato, Cagliari, Italy

176. Unita` Operativa Semplice Cardiologia, Divisione di Medicina, Presidio Ospedaliero Santa Barbara, Iglesias, Italy

177. Computational Medicine Research Group, Institute of Clinical Medicine, University of Oulu and Biocenter Oulu, 90014 University of Oulu, Oulu, Finland

178. NMR Metabonomics Laboratory, Department of Biosciences, University of Eastern Finland, 70211 Kuopio, Finland

179. Department of Internal Medicine and Biocenter Oulu, Clinical Research Center, 90014 University of Oulu, Oulu, Finland

180. Institute for Molecular Medicine Finland FIMM, 00014 University of Helsinki, Helsinki, Finland

181. Department of Biomedical Engineering and Computational Science, School of Science and Technology, Aalto University, 00076 Aalto, Espoo, Finland

182. NUS Graduate School for Integrative Sciences & Engineering (NGS) Centre for Life Sciences (CeLS), Singapore, 117456, Singapore

183. Department of Internal Medicine B, Ernst-Moritz-Arndt-University Greifswald, 17487 Greifswald, Germany

184. Institute of Pharmacology, Ernst-Moritz-Arndt-University Greifswald, 17487 Greifswald, Germany

185. Institute for Community Medicine, Ernst-Moritz-Arndt-University Greifswald, 17487 Greifswald, Germany

186. U557 Institut National de la Santé et de la Recherche Médicale, U1125 Institut National de la Recherche Agronomique, Université Paris 13, Bobigny, France

187. Department of Neurology, Mayo Clinic, Jacksonville, FL, USA

188. Imperial College Cerebrovascular Unit (ICCRU), Imperial College, London, W6 8RF, UK

189. Faculty of Medicine, University of Split, Croatia

190. Department of Internal Medicine, Diabetology, and Nephrology, Medical University of Silesia, 41-800, Zabrze, Poland

191. Public Health Sciences section, Division of Community Health Sciences, University of Edinburgh, Medical School, Teviot Place, Edinburgh, EH8 9AG, UK

192. School of Science and Engineering, University of Ballarat, 3353 Ballarat, Australia

193. Prevention and Care of Diabetes, Department of Medicine III, Medical Faculty Carl Gustav Carus at the Technical University of Dresden, 01307 Dresden, Germany

194. University Hospital Münster, Internal Medicine D, Münster, Germany

195. Department of Medical Statistics, Epidemiology and Medical Informatics, Andrija Stampar School of Public Health, University of Zagreb, Croatia

196. AstraZeneca R&D, 431 83 Mölndal, Sweden

197. Genetic Epidemiology & Biostatistics Platform, Ontario Institute for Cancer Research, Toronto

198. Samuel Lunenfeld Institute for Medical Research, University of Toronto, Canada

199. Faculty of Epidemiology and Population Health, London School of Hygiene and Tropical Medicine, UK

200. Department of Epidemiology and Public Health, Imperial College, Norfolk Place London W2 1PG, UK

201. Research Centre of Applied and Preventive Cardiovascular Medicine, University of Turku and the Department of Clinical Physiology, Turku University Hospital, Turku, 20521, Finland

202. Department of Internal Medicine, Division of Cardiovascular Medicine, University of Michigan Medical Center, Ann Arbor, Michigan, USA

203. Singapore Eye Research Institute, Singapore, 168751, Singapore

204. Department of Ophthalmology, National University of Singapore, Singapore, 119074, Singapore

205. Department of Medicine, Yong Loo Lin School of Medicine, National University of Singapore, Singapore, 119074, Singapore

206. Duke-National University of Singapore Graduate Medical School, Singapore, 169857, Singapore

207. Division of Biostatistics, Washington University School of Medicine, Saint Louis, MO, 63110, USA

208. Department of Primary Care & Population Health, UCL, London, UK, NW3 2PF, UK

209. BHF Glasgow Cardiovascular Research Centre, University of Glasgow, 126 University Place, Glasgow, G12 8TA, UK

210. Epidemiology Public Health, UCL, London, UK, WC1E 6BT, UK

211. Departments of Epidemiology, Biostatistics, and Medicine, Johns Hopkins University, Baltimore MD, USA

212. Division of Nephrology, Department of Internal Medicine, University Medical Center Groningen, University of Groningen, The Netherlands

213. Division of Cardiology, Brigham and Women's Hospital, 900 Commonwealth Avenue East, Boston MA 02215, USA

214. Geriatric Rehabilitation Unit, Azienda Sanitaria Firenze (ASF), Florence, Italy

215. National Institute for Health and Welfare, Diabetes Prevention Unit, 00271 Helsinki, Finland

216. Hjelt Institute, Department of Public Health, University of Helsinki, 00014 Helsinki, Finland

217. South Ostrobothnia Central Hospital, 60220 Seinäjoki, Finland

218. Red RECAVA Grupo RD06/0014/0015, Hospital Universitario La Paz, 28046 Madrid, Spain

219. Department of Neurology, General Central Hospital, 39100 Bolzano, Italy

220. CIBER Epidemiología y Salud Pública, 08003 Barcelona

221. Department of Medicine and Department of Genetics, Harvard Medical School, Boston, Massachusetts 02115, USA

222. Geriatric Research and Education Clinical Center, Veterans Administration Medical Center, Baltimore, MD, USA

223. U872 Institut National de la Santé et de la Recherche Médicale, Centre de Recherche des Cordeliers, Paris, France

224. Institute of Physiology, Ernst-Moritz-Arndt-University Greifswald, 17487 Greifswald, Germany

225. Institute of Clinical Medicine/Obstetrics and Gynecology, University of Oulu, Finland

226. Service of Medical Genetics, Centre Hospitalier Universitaire Vaudois, 1011 Lausanne, Switzerland

227. Human Genetics Center, 1200 Hermann Pressler, Suite E447 Houston, TX 77030, USA

228. Division of Epidemiology and Prevention, Boston University School of Medicine, Boston, MA, USA

229. Department of Mathematics, Boston University, Boston, MA, USA

230. Institute of Health Sciences, University of Oulu, BOX 5000, 90014 University of Oulu, Finland

231. Biocenter Oulu, University of Oulu, BOX 5000, 90014 University of Oulu, Finland

232. National Institute for Health and Welfare, Box 310, 90101 Oulu, Finland

233. MRC-HPA Centre for Environment and Health, School of Public Health, Imperial College London, Norfolk Place, London W2 1PG, UK

234. Centre of Medical Systems Biology (CMSB 1-2), NGI Erasmus Medical Center, Rotterdam, The Netherlands

**The ICBP/CHARGE/Global-BPGen Consortia (age-specific SNP look-ups on blood pressure traits)**

Jeannette Simino^1^, Gang Shi^1^, Joshua C. Bis^2^, Daniel I. Chasman^3,4^, Georg B. Ehret^5,6^, Xiangjun Gu^7^, Xiuqing Guo^8^, Shih-Jen Hwang^9,10^, Eric Sijbrands^11^, Albert V. Smith^12,13^, Germaine C. Verwoert^11,14^, Jennifer L. Bragg-Gresham^15^, Gemma Cadby^16,17,18^, Peng Chen^19,20^, Ching-Yu Cheng^19,20,21,22,23,24^, Tanguy Corre^25,26^, Rudolf A. de Boer^27^, Anuj Goel^28,29^, Toby Johnson^30^, Chiea-Chuen Khor^19,20,21,22,31,32,33^, LifeLines Cohort Study^,^ Carla Lluı´s-Ganella^34^, Jian’an Luan^35^, Leo-Pekka Lyytika¨inen^36,37^, Ilja M. Nolte^38^, Xueling Sim^15,39^, Siim So˜ber^40^, Peter J. van der Most^38^, Niek Verweij^27^, Jing Hua Zhao^35^, Najaf Amin^14^, Eric Boerwinkle^41^, Claude Bouchard^42^, Abbas Dehghan^14^, Gudny Eiriksdottir^12^, Roberto Elosua^34,43^, Oscar H. Franco^14^, Christian Gieger^44^, Tamara B. Harris^45^, Serge Hercberg^46^, Albert Hofman^14^, Alan L. James^47,48^, Andrew D. Johnson^9,49^, Mika Ka¨ho¨nen^50,51^, Kay-Tee Khaw^52^, Zoltan Kutalik^25,26^, Martin G. Larson^9,53^, Lenore J. Launer^45^, Guo Li^2^, Jianjun Liu^19,20,31^, Kiang Liu^54^, Alanna C. Morrison^41^, Gerjan Navis^55^, Rick Twee-Hee Ong^19,20^, George J. Papanicolau^56^, Brenda W. Penninx^57,58,59^, Bruce M. Psaty^2,60,61,62^, Leslie J. Raffel^63^, Olli T. Raitakari^64,65^, Kenneth Rice^66^, Fernando Rivadeneira^11,14^, Lynda M. Rose^3^, Serena Sanna^67^, Robert A. Scott^35^, David S. Siscovick^2,60^, Ronald P. Stolk^38^, Andre G. Uitterlinden^11,14,68^, Dhananjay Vaidya^69^, Melanie M. van der Klauw^70^, Ramachandran S. Vasan^9,71^, Eranga Nishanthie Vithana^21,22,23,72^, Uwe Völker^73^, Henry Vo¨lzke^74^, Hugh Watkins^28,29^, Terri L. Young^75,76^, Tin Aung^21,22,23^, Murielle Bochud^77^, Martin Farrall^28,29^, Catharina A. Hartman^78^, Maris Laan^40^, Edward G. Lakatta^79^, Terho Lehtima¨ki^36,37^, Ruth J.F. Loos^35,80,81,82^, Gavin Lucas^34^, Pierre Meneton^83^, Lyle J. Palmer^17,18^, Rainer Rettig^84^, Harold Snieder^38^, E. Shyong Tai^19,20,85,86^, Yik-Ying Teo^19,20,87,88,89^, Pim van der Harst^27,90,91^, Nicholas J. Wareham^35^, Cisca Wijmenga^90^, Tien Yin Wong^21,22,23^, Myriam Fornage^7,41^, Vilmundur Gudnason^12,13^, Daniel Levy^9,10,92^, Walter Palmas^93^, Paul M. Ridker^3,4^, Jerome I. Rotter^8^, Cornelia M. van Duijn^14,68,94^, Jacqueline C.M. Witteman^14^, Aravinda Chakravarti^5,^ Dabeeru C. Rao^1,95^

1. Division of Biostatistics, Washington University School of Medicine, St. Louis, MO 63110, USA

2. Cardiovascular Health Research Unit, Department of Medicine, University of Washington, Seattle, WA 98101, USA

3. Division of Preventive Medicine, Brigham and Women’s Hospital, Boston, MA 02215, USA

4. Harvard Medical School, Boston, MA 02115, USA

5. Center for Complex Disease Genomics, McKusick-Nathans Institute of Genetic Medicine, Johns Hopkins University School of Medicine, Baltimore, MD 21205, USA

6. Cardiology, Department of Specialties of Internal Medicine, Geneva University Hospitals, Geneva 1211, Switzerland

7. Research Center for Human Genetics, Brown Foundation Institute of Molecular Medicine, University of Texas Health Science Center, Houston, TX 77030, USA

8. Institute for Translational Genomics and Population Sciences, Los Angeles Biomedical Research Institute and Department of Pediatrics, Harbor-UCLA Medical Center, Torrance, CA 90502, USA

9. Framingham Heart Study, Framingham, MA 01702, USA

10. Center for Population Studies, National Heart, Lung, and Blood Institute, Framingham, MA 01702, USA

11. Department of Internal Medicine, Erasmus University Medical Center, 3015 GE Rotterdam, the Netherlands

12. Icelandic Heart Association, 201 Kopavogur, Iceland

13. Faculty of Medicine, University of Iceland, 101 Reykjavik, Iceland

14. Department of Epidemiology, Erasmus University Medical Center, 3015 GE Rotterdam, the Netherlands

15. Center for Statistical Genetics, University of Michigan, Ann Arbor, MI 48109, USA

16. Centre for Genetic Origins of Health and Disease, University of Western Australia, Nedlands, WA 6009, Australia

17. Genetic Epidemiology and Biostatistics Platform, Ontario Institute for Cancer Research, Toronto, ON M5G 0A3, Canada

18. Samuel Lunenfeld Research Institute, Toronto, ON M5T 3L9, Canada

19. Saw Swee Hock School of Public Health, National University of Singapore, Singapore 117597, Singapore

20. Saw Swee Hock School of Public Health, National University Health System, Singapore 117597, Singapore

21. Department of Ophthalmology, Yong Loo Lin School of Medicine, National University of Singapore, Singapore 119228, Singapore

22. Department of Ophthalmology, National University Health System, Singapore 119228, Singapore

23. Singapore Eye Research Institute, Singapore 168751, Singapore

24. Centre for Quantitative Medicine, Office of Clinical Sciences, Duke-NUS Graduate Medical School, Singapore 169857, Singapore

25. Department of Medical Genetics, University of Lausanne, 1005 Lausanne, Switzerland

26. Swiss Institute of Bioinformatics, 1015 Lausanne, Switzerland

27. Department of Cardiology, University of Groningen, UniversityMedical Center Groningen, 9700 RB Groningen, the Netherlands

28. Wellcome Trust Centre for Human Genetics, University of Oxford, Oxford OX3 7BN, UK

29. Division of Cardiovascular Medicine, Radcliffe Department of Medicine, University of Oxford, Oxford OX3 9DU, UK

30. Clinical Pharmacology, Barts and The London School of Medicine and Dentistry, Queen Mary University of London, London EC1M 6BQ, UK

31. Division of Human Genetics, Genome Institute of Singapore, Singapore 138672, Singapore

32. Department of Paediatrics, Yong Loo Lin School of Medicine, National University of Singapore, Singapore 119228, Singapore

33. Department of Paediatrics, National University Health System, Singapore 119074, Singapore

34. Cardiovascular Epidemiology and Genetics, IMIM (Hospital del Mar Medical Research Institute), 08003 Barcelona, Spain

35. MRC Epidemiology Unit, Institute of Metabolic Science, University of Cambridge, Cambridge CB2 0QQ, UK

36. Department of Clinical Chemistry, Fimlab Laboratories, Tampere 30101, Finland

37. Department of Clinical Chemistry, University of Tampere School of Medicine, Tampere 33101, Finland

38. Department of Epidemiology, University of Groningen, University Medical Center Groningen, 9700 RB Groningen, the Netherlands

39. Centre for Molecular Epidemiology, National University of Singapore, Singapore 119260, Singapore

40. Human Molecular Genetics Group, Institute of Molecular and Cell Biology, University of Tartu, Tartu 51010, Estonia

41. Human Genetics Center, University of Texas Health Sciences Center, Houston, TX 77225, USA

42. Human Genomics Laboratory, Pennington Biomedical Research Center, Baton Rouge, LA 70808, USA

43. Epidemiology and Public Health Network (CIBERESP), 08036 Barcelona, Spain

44. Institute of Genetic Epidemiology, Helmholtz Zentrum München—German Research Center for Environmental Health, Ingolstädter Landstr. 1, 85764 Neuherberg, Germany

45. Laboratory of Epidemiology, Demography, and Biometry, National Institute on Aging, NIH, Bethesda, MD 20892, USA

46. U557 Institut National de la Sante´ et de la Recherche Me´dicale, U1125 Institut National de la Recherche Agronomique, Universite´ Paris 13, 93000 Bobigny, France

47. Department of Pulmonary Physiology and Sleep Medicine, Sir Charles Gairdner Hospital, Nedlands,WA 6009, Australia

48. School of Medicine and Pharmacology, University of Western Australia, Nedlands, WA 6009, Australia

49. Cardiovascular Epidemiology and Human Genomics Branch, National Heart, Lung, and Blood Institute, NIH, Bethesda, MD 20892, USA

50. Department of Clinical Physiology, Tampere University Hospital, Tampere 33521, Finland

51. Department of Clinical Physiology, University of Tampere School of Medicine, Tampere 33521, Finland

52. Department of Public Health and Primary Care, Institute of Public Health, University of Cambridge, Cambridge CB2 2SR, UK

53. Department of Mathematics, Boston University, Boston, MA 02215, USA

54. Department of Preventive Medicine, Northwestern University Feinberg School of Medicine, Chicago, IL 60611, USA

55. Department of Internal Medicine, University of Groningen, University Medical Center Groningen, 9700 RB Groningen, the Netherlands

56. Division of Cardiovascular Sciences, National Heart, Lung, & Blood Institute, NIH, Bethesda, MD 20892, USA

57. Department of Psychiatry/EMGO Institute/Neuroscience Campus, VU University Medical Centre, 1081 BT Amsterdam, the Netherlands

58. Department of Psychiatry, Leiden University Medical Centre, 2333 ZD Leiden, the Netherlands

59. Department of Psychiatry, University of Groningen, University Medical Center Groningen, 9700 RB Groningen, the Netherlands

60. Department of Epidemiology, University of Washington, Seattle, WA 98195, USA

61. Department of Health Services, University of Washington, Seattle, WA 98195, USA

62. Group Health Research Institute, Group Health Cooperative, Seattle, WA 98101, USA

63. Medical Genetics Institute, Cedars-Sinai Medical Center, Pacific Theatres, Los Angeles, CA 90048, USA

64. Department of Clinical Physiology and Nuclear Medicine, Turku University Hospital, Turku 20521, Finland

65. Research Centre of Applied and Preventive Cardiovascular Medicine, University of Turku, Turku 20521, Finland

66. Department of Biostatistics, University of Washington, Seattle, WA 98195, USA

67. Istituto di Ricerca Genetica e Biomedica, CNR, Monserrato 09042, Italy

68. Netherland Genomics Inititiative, Netherlands Center for Healthy Aging, The Hague 2509, the Netherlands

69. Department of Medicine, Johns Hopkins University, Baltimore, MD 21202, USA

70. Department of Endocrinology, University of Groningen, University Medical Center Groningen, 9700 RB Groningen, the Netherlands

71. Divisions of Epidemiology and Cardiology, Department of Medicine, Boston University School of Medicine, Boston, MA 02118, USA

72. Neuroscience and Behavioural Disorders (NBD) Program, Duke-NUS Graduate Medical School, Singapore 169857, Singapore

73. Interfaculty Institute for Genetics and Functional Genomics, University of Greifswald, 17487 Greifswald, Germany

74. Institute for Community Medicine, University of Greifswald, 17487 Greifswald, Germany

75. Department of Ophthalmology, Duke University Medical Center, Durham, NC 27710, USA

76. Division of Neuroscience, Duke-National University of Singapore, Singapore 169857, Singapore

77. Institute of Social and Preventive Medicine, Lausanne University Hospital, 1010 Lausanne, Switzerland

78. Interdisciplinary Center for Pathology of Emotions, University of Groningen, University Medical Center Groningen, 9700 RB Groningen, the Netherlands

79. Laboratory of Cardiovascular Science, National Institute on Aging, NIH, Bethesda, MD 21224, USA

80. The Charles Bronfman Institute for Personalized Medicine, Icahn School of Medicine at Mount Sinai, New York, NY 10029, USA

81. The Genetics of Obesity and Related Metabolic Traits Program, The Icahn School of Medicine at Mount Sinai, New York, NY 10029, USA

82. The Mindich Child Health and Development Institute, Icahn School of Medicine at Mount Sinai, New York, NY 10029, USA

83. U872 Institut National de la Sante´ et de la Recherche Me´dicale, Centre de Recherche des Cordeliers, Paris 75006, France

84. Institute of Physiology, University of Greifswald, 17495 Karlsburg, Germany

85. Department of Medicine, National University Health System and Yong Loo Lin School of Medicine, National University of Singapore, Singapore 119228, Singapore

86. Duke-National University of Singapore Graduate Medical School, Singapore 169857, Singapore

87. Life Sciences Institute, National University of Singapore, Singapore 117456, Singapore

88. Department of Statistics and Applied Probability, National University of Singapore, Singapore 117543, Singapore

89. Genome Institute of Singapore, A*STAR, Singapore 138672, Singapore

90. Department of Genetics, University of Groningen, University Medical Center Groningen, 9700 RB Groningen, the Netherlands

91. Durrer Center for Cardiogenetic Research, 3501 DG Utrecht, the Netherlands

92. Boston University School of Medicine, Boston, MA 02118, USA

93. Department of Medicine, Columbia University, New York, NY 10032, USA

94. Netherland Genomics Initiative, Centre for Medical Systems Biology, 2300 RC Leiden, the Netherlands

95. Departments of Psychiatry, Genetics, and Mathematics, Washington University School of Medicine, St. Louis, MO 63110, USA

**The MAGIC Consortium (sex-specific SNP look-ups on glycemic traits)**

Robert A Scott^1^, Vasiliki Lagou^2,3^, Ryan P Welch^4,6^, Eleanor Wheeler^7^, May E Montasser^8^, Jian’an Luan^1^, Reedik Mägi^2,9^, Rona J Strawbridge^10,11^, Emil Rehnberg^12^, Stefan Gustafsson^12^, Stavroula Kanoni^7^, Laura J Rasmussen-Torvik^13^, Loïc Yengo^14,15^, Cecile Lecoeur^14,15^, Dmitry Shungin^16,18^, Serena Sanna^19^, Carlo Sidore^5,6,19,20^ Paul C D Johnson^21^, J Wouter Jukema^22,23^, Toby Johnson^24,25^, Anubha Mahajan^2^, Niek Verweij^26^, Gudmar Thorleifsson^27^, Jouke-Jan Hottenga^28^, Sonia Shah^29^, Albert V Smith^30,31^, Bengt Sennblad^10^ Christian Gieger^32^, Perttu Salo^33^, Markus Perola^9,33,34^, Nicholas J Timpson^35^, David M Evans^35^, Beate St Pourcain^36^, Ying Wu^37^, Jeanette S Andrews^38^, Jennie Hui^39,40,41,42^, Lawrence F Bielak^43^, Wei Zhao^43^, Momoko Horikoshi^2,3^, Pau Navarro^44^, Aaron Isaacs^45,46^, Jeffrey R O’Connell^8^, Kathleen Stirrups^7^, Veronique Vitart^44^, Caroline Hayward^44^, Tönu Esko^9,47^, Evelin Mihailov^47^, Ross M Fraser^48^, Tove Fall^12^, Benjamin F Voight^49,50^ Soumya Raychaudhuri^51^, Han Chen^52^, Cecilia M Lindgren^2^, Andrew P Morris^2^, Nigel W Rayner^2,3^, Neil Robertson^2,3^, Denis Rybin^53^, Ching-Ti Liu^52^, Jacques S Beckmann^54,55^, Sara M Willems^46^, Peter S Chines^56^, Anne U Jackson^5,6^, Hyun Min Kang^5,6^, Heather M Stringham^5,6^, Kijoung Song^57^, Toshiko Tanaka^58^, John F Peden^2,59^, Anuj Goel^2,60^ Andrew A Hicks^61^, Ping An^62^, Martina Müller-Nurasyid^32,63,64^, Anders Franco-Cereceda^65^, Lasse Folkersen^10,11^, Letizia Marullo^2,66^, Hanneke Jansen^67^, Albertine J Oldehinkel^68^, Marcel Bruinenberg^69^, James S Pankow^70^ Kari E North^71,72^, Nita G Forouhi^1^, Ruth J F Loos^1^, Sarah Edkins^7^, Tibor V Varga^16^, Göran Hallmans^73^, Heikki Oksa^74^, Mulas Antonella^19^, Ramaiah Nagaraja^75^, Stella Trompet^22,23^, Ian Ford^21^, Stephan J L Bakker^76^, Augustine Kong^27^, Meena Kumari^77^, Bruna Gigante^78^, Christian Herder^79^, Patricia B Munroe^24,25^, Mark Caulfield^24,25^, Jula Antti^33^, Massimo Mangino^80^ Kerrin Small^80^ Iva Miljkovic^81^, Yongmei Liu^82^, Mustafa Atalay^83^, Wieland Kiess^84,85^, Alan L James^39,86,87^, Fernando Rivadeneira^45,88,90^ Andre G Uitterlinden^45,88,89,90^ Colin N A Palmer^91^, Alex S F Doney^91^, Gonneke Willemsen^28^, Johannes H Smit^92^, Susan Campbell^44^, Ozren Polasek^93^, Lori L Bonnycastle^56^, Serge Hercberg^94^, Maria Dimitriou^95^, Jennifer L Bolton^96^, Gerard R Fowkes^96^, Peter Kovacs^97^, Jaana Lindström^98^, Tatijana Zemunik^93^, Stefania Bandinelli^99^, Sarah H Wild^48^, Hanneke V Basart^100^ Wolfgang Rathmann^101^, Harald Grallert^102^, Winfried Maerz^104,105^, Marcus E Kleber^105,106^, Bernhard O Boehm^107^, Annette Peters^108^, Peter P Pramstaller^61,109,110^ Michael A Province^62^, Ingrid B Borecki^62^, Nicholas D Hastie^44^, Igor Rudan^48^, Harry Campbell^48^, Hugh Watkins^2,60^ Martin Farrall^2,60^ Michael Stumvoll^84,111^, Luigi Ferrucci^58^, Dawn M Waterworth^57^, Richard N Bergman^112^, Francis S Collins^56^, Jaakko Tuomilehto^113,114,115,116^, Richard M Watanabe^117,118^, Eco J C de Geus^28^, Brenda W Penninx^92^, Albert Hofman^90^ Ben A Oostra^45,46,89^, Bruce M Psaty^119,120,121,122^, Peter Vollenweider^123^, James F Wilson^48^, Alan F Wright^44^, G Kees Hovingh^100^ Andres Metspalu^9,47^, Matti Uusitupa^124,125^, Patrik K E Magnusson^12^, Kirsten O Kyvik^126,127^, Jaakko Kaprio^34,128,129^, Jackie F Price^96^, George V Dedoussis^95^, Panos Deloukas^7^, Pierre Meneton^130^ Lars Lind^131^, Michael Boehnke^5,6^, Alan R Shuldiner^8,132^, Cornelia M van Duijn^45,46,89,90^ Andrew D Morris^91^, Anke Toenjes^84,111^, Patricia A Peyser^43^, John P Beilby^39,41,42^, Antje Körner^84,85^, Johanna Kuusisto^133^, Markku Laakso^133^, Stefan R Bornstein^134^, Peter E H Schwarz^134^, Timo A Lakka^83,135^, Rainer Rauramaa^135,136^, Linda S Adair^137^, George Davey Smith^35^, Tim D Spector^80^ Thomas Illig^102,138^, Ulf de Faire^78^, Anders Hamsten^10,11,139^, Vilmundur Gudnason^30,31^, Mika Kivimaki^77^, Aroon Hingorani^77^, Sirkka M Keinanen-Kiukaanniemi^140,141^, Timo E Saaristo^74,142^, Dorret I Boomsma^28^, Kari Stefansson^27,31^, Pim van der Harst^26^, Josée Dupuis^52,143^, Nancy L Pedersen^12^, Naveed Sattar^144^, Tamara B Harris^145^, Francesco Cucca^19,20^ Samuli Ripatti^146,147,148^, Veikko Salomaa^149^, Karen L Mohlke^37^, Beverley Balkau^150,151^, Philippe Froguel^14,15,152^, Anneli Pouta^153,154^, Marjo-Riitta Jarvelin^154,155,156,157^, Nicholas J Wareham^1^, Nabila Bouatia-Naji^14,15,158^, Mark I McCarthy^2,3,159^, Paul W Franks^16,17,160^ James B Meigs^161,162^, Tanya M Teslovich^5,6^, Jose C Florez^162,165^, Claudia Langenberg^1,77^, Erik Ingelsson^12^, Inga Prokopenko^2,3^, and Inês Barroso^7,166,167^

1. Medical Research Council (MRC) Epidemiology Unit, Institute of Metabolic Science, Addenbrooke’s Hospital, Cambridge, UK.

2. Wellcome Trust Center for Human Genetics, University of Oxford, Oxford, UK.

3. Oxford Centre for Diabetes, Endocrinology and Metabolism, University of Oxford, Oxford, UK.

4. Bioinformatics Graduate Program, University of Michigan Medical School, Ann Arbor, Michigan, USA.

5. Center for Statistical Genetics, University of Michigan, Ann Arbor, Michigan, USA.

6. Department of Biostatistics, University of Michigan, Ann Arbor, Michigan, USA.

7. Wellcome Trust Sanger Institute, Wellcome Trust Genome Campus, Hinxton, UK.

8. Division of Endocrinology, Diabetes and Nutrition, University of Maryland, School of Medicine, Baltimore, Maryland, USA.

9. Estonian Genome Center, University of Tartu, Tartu, Estonia.

10. Atherosclerosis Research Unit, Department of Medicine Solna, Karolinska Institutet, Stockholm, Sweden.

11. Center for Molecular Medicine, Karolinska University Hospital, Stockholm, Sweden.

12. Department of Medical Epidemiology and Biostatistics, Karolinska Institutet, Stockholm, Sweden.

13. Department of Preventive Medicine, Northwestern University Feinberg School of Medicine, Chicago, Illinois, USA.

14. Universite Lille Nord de France, Lille, France.

15. Le Centre national de la recherche scientifique (CNRS) UMR8199, Institut Pasteur de Lille, France.

16. Department of Clinical Sciences, Genetic and Molecular Epidemiology Unit, Lund University, Skåne University Hospital Malmö, Malmö, Sweden.

17. Department of Public Health & Clinical Medicine, Umeå University, Umeå, Sweden.

18. Department of Odontology, Umeå University, Umeå, Sweden.

19. Istituto di Ricerca Genetica e Biomedica, CNR, Monserrato, Italy.

20. Dipartimento di Scienze Biomediche, Università di Sassari, Sassari, Italy.

21. Robertson Centre for Biostatistics, University of Glasgow, Glasgow, UK.

22. Interuniversity Cardiology Institute of the Netherlands (ICIN), Durrer Center for Cardiogenetic Research, Utrecht, The Netherlands.

23. Departmentt of Cardiology, Leiden University Medical Center, Leiden, The Netherlands.

24. Department of Clinical Pharmacology, William Harvey Research Institute, Barts and The London School of Medicine and Dentistry, Queen Mary University of London, Charterhouse Square, London, UK.

25. The Genome Centre, Barts and The London School of Medicine and Dentistry, Queen Mary University of London, Charterhouse Square, London, UK.

26. Department of Cardiology, University of Groningen, University Medical Center Groningen, The Netherlands.

27. deCODE genetics, Rekjavik, Iceland.

28. Department of Biological Psychology, VU University & EMGO+ Institute, Amsterdam, The Netherlands.

29. University College London Genetics Institute (UGI), University College London, London, UK.

30. Icelandic Heart Association, Kopavogur, Iceland.

31. Faculty of Medicine, University of Iceland, Reykjavìk, Iceland.

32. Institute of Genetic Epidemiology, Helmholtz Zentrum München - German Research Center for Environmental Health, Neuherberg, Germany.

33. Department of Chronic Disease Prevention, National Institute for Health and Welfare, Helsinki, Finland.

34. University of Helsinki, Institute of Molecular Medicine, Finland (FIMM), Helsinki, Finland.

35. MRC Council Centre for Causal Analyses in Translational Epidemiology (CAiTE) Centre, School of Social and Community Medicine, University of Bristol, UK.

36. School of Social and Community Medicine, University of Bristol, UK.

37. Department of Genetics, University of North Carolina, Chapel Hill, North Carolina, USA.

38. Department of Biostatistical Sciences, Division of Public Health Sciences, Wake Forest School of Medicine, Winston-Salem, North Carolina, USA.

39. Busselton Population Medical Research Institute, Sir Charles Gairdner Hospital, Nedlands, Western Australia, Australia.

40. School of Population Health, The University of Western Australia, Nedlands, Western Australia, Australia.

41. School of Pathology and Laboratory Medicine, The University of Western Australia, Nedlands, Western Australia, Australia.

42. PathWest Laboratory Medicine WA, Nedlands, Western Australia, Australia.

43. Department of Epidemiology, School of Public Health, University of Michigan, Ann Arbor, Michigan, USA.

44. MRC Human Genetics Unit at the Medical Research Council Institute of Genetics and Molecular Medicine, University of Edinburgh, Western General Hospital, Edinburgh, UK.

45. Centre for Medical Systems Biology (CMSB), Leiden, The Netherlands.

46. Genetic Epidemiology Unit, Department of Epidemiology, Erasmus University Medical Center, Rotterdam, The Netherlands.

47. Institute of Molecular and Cell Biology, University of Tartu, Tartu, Estonia.

48. Centre for Population Health Sciences, University of Edinburgh, Teviot Place, Edinburgh, UK.

49. The Broad Institute of Harvard and MIT, Boston, Massachusetts, USA.

50. Department of Pharmacology, University of Pennsylvania Perelman School of Medicine, Philadelphia, Pennsylvania, USA.

51. Divisions of Genetics & Rheumatology, Brigham and Women’s Hospital, Boston, Massachusetts, USA.

52. Department of Biostatistics, Boston University School of Public Health, Boston, Massachusetts, USA.

53. Boston University Data Coordinating Center, Boston, Massachusetts, USA.

54. Department of Medical Genetics, University of Lausanne, Lausanne, Switzerland.

55. The Service of Medical Genetics, CHUV, University Hospital, Lausanne Switzerland.

56. Genome Technology Branch, National Human Genome Research Institute, National Institutes of Health (NIH), Bethesda, Maryland, USA.

57. Genetics, GlaxoSmithKline, Upper Merion, Pennsylvania, USA.

58. Clinical Research Branch, National Institute on Aging, Baltimore, Maryland, USA.

59. Illumina Inc., Chesterford Research Park, Essex, UK.

60. Department of Cardiovascular Medicine, University of Oxford, Oxford, UK.

61. Centre for Biomedicine, European Academy Bozen/Bolzano (EURAC), Bolzano, Italy - Affiliated Institute of the University of Lübeck, Lübeck, Germany.

62. Division of Statistical Genomics, Washington University School of Medicine, St. Louis, Missouri, USA.

63. Institute of Medical Informatics, Biometry and Epidemiology, Ludwig-Maximilians-Universität, Munich, Germany.

64. Department of Medicine I, University Hospital Grosshadern, Ludwig-Maximilians-Universität, Munich, Germany.

65. Cardiothoracic Surgery Unit, Department of Molecular Medicine and Surgery, Karolinska Institutet, Stockholm, Sweden.

66. Department of Evolutionary Biology, Genetic Section, University of Ferrara, Ferrara, Italy.

67. Department of Epidemiology, University Medical Center Groningen, University of Groningen, Groningen, The Netherlands.

68. Interdisciplinary Center for Pathology of Emotions, University of Groningen, University Medical Center Groningen, Groningen, The Netherlands.

69. University Medical Center Groningen, University of Groningen, Groningen, The Netherlands.

70. Division of Epidemiology and Community Health, University of Minnesota, Minneapolis, Minnesota, USA.

71. Carolina Center for Genome Sciences, University of North Carolina-Chapel Hill, Chapel Hill, North Carolina, USA.

72. Department of Epidemiology, University of North Carolina-Chapel Hill, Chapel Hill, North Carolina, USA.

73. Department of Public Health and Clinical Medicine, Section for Nutritional Research, Umeå University Hospital, Umeå, Sweden.

74. Pirkanmaa Hospital District, Tampere, Finland.

75. Laboratory of Genetics, National Institute on Aging, NIH, Baltimore, Maryland, USA.

76. Department of Internal Medicine, University of Groningen, University Medical Center Groningen, Groningen, The Netherlands.

77. Department of Epidemiology and Public Health, University College London, London UK.

78. Division of Cardiovascular Epidemiology, Institute of Environmental Medicine, Karolinska Institutet, Stockholm, Sweden.

79. Institute for Clinical Diabetology, German Diabetes Center, Leibniz Center for Diabetes Research at Heinrich Heine University Düsseldorf, Düsseldorf, Germany.

80. Department of Twin Research and Genetic Epidemiology, King’s College London, London, UK.

81. Department of Epidemiology, Center for Aging and Population Health, University of Pittsburgh, Pittsburgh, Pennsylvania, USA.

82. Department of Epidemiology and Prevention, Division of Public Health Sciences, Wake Forest School of Medicine, Winston-Salem, North Carolina, USA.

83. Institute of Biomedicine, Physiology, University of Eastern Finland, Kuopio Campus, Kuopio, Finland.

84. University of Leipzig, IFB Adiposity Diseases, Leipzig, Germany.

85. Pediatric Research Center, Department of Women’s & Child Health, University of Leipzig, Leipzig, Germany.

86. School of Medicine and Pharmacology, The University of Western Australia, Nedlands, Western Australia, Australia.

87. Pulmonary Physiology, Sir Charles Gairdner Hospital, Nedlands, Western Australia, Australia.

88. Department of Internal Medicine, Erasmus University Medical Center, Rotterdam, The Netherlands.

89. Netherlands Consortium for Healthy Ageing of the Netherlands (NCHAH) of the Genomics Initiative (NGI), Leiden, The Netherlands.

90. Department of Epidemiology, Erasmus University Medical Center, Rotterdam, The Netherlands.

91. Medical Research Institute, University of Dundee, Dundee, UK.

92. Department of Psychiatry, VU University Medical Centre, Amsterdam, The Netherlands.

93. Faculty of Medicine, University of Split, Split, Croatia.

94. U557 Institut National de la Santé et de la Recherche Médicale, U1125 Institut National de la Recherche Agronomique, Université Paris 13, Bobigny, France.

95. Department of Dietetics-Nutrition, Harokopio University, Athens, Greece.

96. Centre for Population Health Sciences, University of Edinburgh, Edinburgh, UK.

97. University of Leipzig, Interdisciplinary Center for Clinical Research, Leipzig, Germany.

98. National Institute for Health and Welfare, Diabetes Prevention Unit, Helsinki, Finland.

99. Geriatric Department Azienda Sanitaria Firenze, Florence Italy.

100. Department Vascular Medicine, Academic Medical Center, Amsterdam, The Netherlands.

101. Institute of Biometrics and Epidemiology, German Diabetes Center, Leibniz Center for Diabetes Research at Heinrich Heine University Düsseldorf, Düsseldorf, Germany.

102. Research Unit of Molecular Epidemiology, Helmholtz Zentrum München - German Research Center for Environmental Health, Neuherberg, Germany.

104. Synlab Academy, Mannheim, Germany.

105. Mannheim Institute of Public Health, Social and Preventive Medicine, Medical Faculty of Mannheim, University of Heidelberg, Mannheim, Germany.

106. Ludwigshafen Risk and Cardiovascular Health (LURIC) Study nonprofit LLC, Freiburg, Germany.

107. Division of Endocrinology and Diabetes, Department of Medicine, University Hospital, Ulm, Germany.

108. Institute of Epidemiology II, Helmholtz Zentrum München - German Research Center for Environmental Health, Neuherberg, Germany.

109. Department of Neurology, General Central Hospital, Bolzano, Italy.

110. Department of Neurology, University of Lübeck, Lübeck, Germany.

111. Department of Medicine, University of Leipzig, Leipzig, Germany.

112. Diabetes and Obesity Research Institute, Cedars-Sinai Medical Center, Los Angeles, California, USA.

113. Diabetes Prevention Unit, National Institute for Health and Welfare, Helsinki, Finland.

114. South Ostrobothnia Central Hospital, Seinäjoki, Finland.

115. Red RECAVA Grupo RD06/0014/0015, Hospital Universitario La Paz, Madrid, Spain.

116. Centre for Vascular Prevention, Danube-University Krems, Krems, Austria.

117. Department of Preventive Medicine, Keck School of Medicine of USC, Los Angeles, California, USA.

118. Department of Physiology & Biophysics, Keck School of Medicine of USC, Los Angeles, California, USA.

119. Cardiovascular Health Research Unit, Departments of Medicine, University of Washington, Seattle, Washington, USA.

120. Group Health Research Institute, Group Health Cooperative, Seattle, Washington, USA.

121. Department of Epidemiology, University of Washington, Seattle, Washington, USA.

122. Department of Health Services, University of Washington, Seattle, Washington, USA.

123. Department of Internal Medicine, University Hospital and University of Lausanne, Lausanne, Switzerland.

124. Department of Public Health and Clinical Nutrition, University of Eastern Finland, Kuopio, Finland.

125. Research Unit, Kuopio University Hospital, Kuopio, Finland.

126. Odense Patient data Explorative Network (OPEN), Odense, Denmark.

127. Institute of Regional Health Services Research, Odense, Denmark.

128. Hjelt Institute, Department of Public Health, University of Helsinki, Helsinki, Finland.

129. National Institute for Health and Welfare, Department of Mental Health and Substance Abuse Services, Helsinki, Finland.

130. U872 Institut National de la Santé et de la Recherche Médicale, Centre de Recherche des Cordeliers, Paris, France.

131. Department of Medical Sciences, Uppsala University, Uppsala, Sweden.

132. Geriatric Research and Education Clinical Center, Veterans Administration Medical Center, Baltimore, Maryland, USA.

133. Department of Medicine, University of Eastern Finland and Kuopio University Hospital, Kuopio, Finland.

134. Department of Medicine III, University of Dresden, Medical Faculty Carl Gustav Carus, Dresden, Germany.

135. Kuopio Research Institute of Exercise Medicine, Kuopio, Finland.

136. Department of Clinical Physiology and Nuclear Medicine, Kuopio University Hospital, Kuopio, Finland.

137. Department of Nutrition, University of North Carolina, Chapel Hill, North Carolina, USA.

138. Hannover Unified Biobank, Hannover Medical School, Hannover, Germany.

139. Department of Cardiology, Karolinska University Hospital, Stockholm, Sweden.

140. Faculty of Medicine, Institute of Health Sciences, University of Oulu, Oulu, Finland.

141. Unit of General Practice, Oulu University Hospital, Oulu, Finland.

142. Finnish Diabetes Association, Tampere, Finland.

143. National Heart, Lung, and Blood Institute’s Framingham Heart Study, Framingham, Massachusetts, USA.

144. British Heart Foundation (BHF) Building, Institute of Cardiovascular and Medical Sciences, University of Glasgow, Glasgow, UK.

145. Laboratory of Epidemiology, Demography, and Biometry, National Institute on Ageing, Bethesda, Maryland, USA.

146. Institute for Molecular Medicine Finland, FIMM, University of Helsinki, Helsinki, Finland.

147. Public Health Genomics Unit, National Institute for Health and Welfare, Helsinki, Finland.

148. Wellcome Trust Sanger Institute, Hinxton, UK.

149. Unit of Chronic Disease Epidemiology and Prevention, National Institute for Health and Welfare, Helsinki, Finland.

150. Inserm, Centre de recherche en Épidémiologie et Santé des Populations (CESP) Center for Research in Epidemiology and Public Health, U1018, Epidemiology of diabetes, obesity and chronic kidney disease over the lifecourse, Villejuif, France.

151. University Paris Sud 11, UMRS 1018, Villejuif, France.

152. Department of Genomics of Common Disease, School of Public Health, Imperial College London, Hammersmith Hospital, London, UK.

153. Department of Clinical Sciences/Obstetrics and Gynecology, University of Oulu, Oulu, Finland.

154. Department of Lifecourse and Services, National Institute for Health and Welfare, Oulu, Finland.

155. Biocenter Oulu, University of Oulu, Oulu, Finland.

156. Department of Epidemiology and Biostatistics, School of Public Health, MRC-HPA Centre for Environment and Health, Faculty of Medicine, Imperial College London, London, UK.

157. Institute of Health Sciences, University of Oulu, Oulu, Finland.

158. Inserm U970, Paris Cardiovascular Research Center PARCC, Paris, France.

159. Oxford National Institute for Health Research (NIHR) Biomedical Research Centre, Churchill Hospital, Oxford, UK.

160. Department of Nutrition, Harvard School of Public Health, Boston, Massachusetts, USA.

161. General Medicine Division, Massachusetts General Hospital, Boston, Massachusetts, USA.

162. Department of Medicine, Harvard Medical School, Boston, Massachusetts, USA.

163. Center for Human Genetic Research, Massachusetts General Hospital, Boston, Massachusetts, USA.

164. Diabetes Research Center, Diabetes Unit, Massachusetts General Hospital, Boston, Massachusetts, USA.

165. Program in Medical and Population Genetics, Broad Institute, Cambridge, Massachusetts, USA.

166. NIHR Cambridge Biomedical Research Centre, Institute of Metabolic Science, Addenbrooke’s Hospital, Cambridge, UK.

167. University of Cambridge Metabolic Research Laboratories, Institute of Metabolic Science, Addenbrooke’s Hospital, Cambridge, UK.

**Extended Acknowledgements**

**Cohort Acknowledgements (funding, personal, groups…)**

ADVANCE The ADVANCE study was supported by a grant from the Reynold's Foundation and NHLBI grant HL087647.

AMC-PAS AMC-PAS is greatful to M.D. Trip MD, PhD and S. Sivapalaratnam MD for their input in collecting the

Data.

AGES The Age, Gene/Environment Susceptibility Reykjavik Study has been funded by NIH contract N01-AG-12100, the NIA Intramural Research Program, Hjartavernd (the Icelandic Heart Association), and the Althingi (the Icelandic Parliament). The study is approved by the Icelandic National Bioethics Committee, (VSN: 00-063) and the Data Protection Authority. The researchers are indebted to the participants for their willingness to participate in the study.

AMISH We gratefully acknowledge our Amish liaisons, field workers and clinic staff and the extraordinary cooperation and support of the Amish community without which these studies would not have been possible. The Amish studies are supported by grants and contracts from the NIH, including U01 HL072515-06, U01 HL84756, U01HL105198, U01 GM074518, F32AR059469, from NIDDK, including P30 DK072488, the University of Maryland General Clinical Research Center, grant M01 RR 16500, by the T32 training grant AG000219, and by National Research Initiative Competitive Grant no. 2007-35205-17883 from the USDA National Institute of Food and Agriculture. We thank our Amish research volunteers for their long-standing partnership in research, and the research staff at the Amish Research Clinic for their hard work and dedication.

arcOGEN arcOGEN (http://www.arcogen.org.uk/) was funded by a special purpose grant from Arthritis Research UK (grant 18030). LS, KP and EZ are supported by the Wellcome Trust (grant 098051). KP is funded by Arthritis Research UK (19542).

ARIC The Atherosclerosis Risk in Communities Study is carried out as a collaborative study supported by National Heart, Lung, and Blood Institute contracts N01-HC-55015, N01-HC-55016, N01-HC-55018, N01-HC-55019, N01-HC-55020, N01-HC-55021, N01-HC-55022, R01HL087641, R01HL59367 and R01HL086694; National Human Genome Research Institute contract U01HG004402; and National Institutes of Health contract HHSN268200625226C. Infrastructure was partly supported by Grant Number UL1RR025005, a component of the National Institutes of Health and NIH Roadmap for Medical Research. The project described was supported by Grant Number UL1 RR 025005 from the National Center for Research Resources (NCRR), a component of the National Institutes of Health (NIH) and NIH Roadmap for Medical Research, and its contents are solely the responsibility of the authors and do not necessarily represent the official view of NCRR or NIH. The authors thank the staff and participants of the ARIC Study for their important contributions.

Athero–Express Genotyping was funded by Cavadis B.V. Sander W. Van der Laan is funded through grants from

Biobank Study Interuniversity Cardiology Institute of the Netherlands (ICIN, 09.001) and CVON (GENIUS). Folkert W. Asselbergs is supported by the UCL Hospitals NIHR Biomedical Research Centre. Claudia Tersteeg, Krista den Ouden, Mirjam B. Smeets, and Loes B. Collé are graciously acknowledged for their work on the DNA extraction. Astrid E.M.W. Willems, Evelyn Velema, Kristy M. J. Vons, Sara Bregman, Timo R. ten Brinke, Sara van Laar, Louise M. Catanzariti, Joyce E.P. Vrijenhoek, Sander M. van de Weg, Arjan H. Schoneveld, Petra H. Homoed-van der Kraak, and Aryan Vink are graciously acknowledged for their past and continuing work on the Athero-Express Biobank Study. We would also like to thank all the (former) employees involved in the Athero-Express Biobank Study of the Departments of Surgery of the St. Antonius Hospital Nieuwegein and University Medical Center Utrecht for their continuing work. Jessica van Setten is graciously acknowledged for her help in the quality assurance and quality control of the genotype data. We graciously thank the team of Golden Helix Inc. especially Autumn Laughbaum, Bryce Christensen, Christophe Lambert, Greta Linse Peterson, and Gabe Rudy for their continuing support in data analysis. Lastly, we would like to thank all participants of the Athero-Express Biobank Study; without you these kinds of studies would not be possible.

B58C-WTCCC We acknowledge use of phenotype and genotype data from the British 1958 Birth Cohort DNA collection, funded by the Medical Research Council grant G0000934 and the Wellcome Trust grant 068545/Z/02. (http://www.b58cgene.sgul.ac.uk/). Genotyping for the B58C-WTCCC subset was funded by the Wellcome Trust grant 076113/B/04/Z.

B58C-T1DGC The B58C-T1DGC genotyping utilized resources provided by the Type 1 Diabetes Genetics Consortium, a collaborative clinical study sponsored by the National Institute of Diabetes and Digestive and Kidney Diseases (NIDDK), National Institute of Allergy and Infectious Diseases (NIAID), National Human Genome Research Institute (NHGRI), National Institute of Child Health and Human Development (NICHD), and Juvenile Diabetes Research Foundation International (JDRF) and supported by U01 DK062418. B58C-T1DGC GWAS data were deposited by the Diabetes and Inflammation Laboratory, Cambridge Institute for Medical Research (CIMR), University of Cambridge, which is funded by Juvenile Diabetes Research Foundation International, the Wellcome Trust and the National Institute for Health Research Cambridge Biomedical Research Centre; the CIMR is in receipt of a Wellcome Trust Strategic Award (079895).

BHS The Busselton Health Study acknowledges the generous support for the 1994/5 follow-up study from Healthway, Western Australia and the numerous Busselton community volunteers who assisted with data collection and the study participants from the Shire of Busselton. The Busselton Health Study is supported by The Great Wine Estates of the Margaret River region of Western Australia. The study gratefully acknowledges the assistance of the Western Australian DNA Bank (NHMRC Enabling Facility) with DNA samples and the support provided by the Ark (NHMRC Enabling Facility) for this study.

BLSA The BLSA was supported by the Intramural Research Program of the NIH, National Institute on Aging.

B-PROOF The entire B-PROOF research team, who participated with study design and data collection.

CAHRES The study was supported by funding from the Agency for Science, Technology and Research of Singapore (A*STAR), the United States National Institute of Health (NIH) and the Susan G. Komen Breast Cancer Foundation.

CARDIOGENICS/ Genotyping was funded by the Wellcome Trust (core grant 098051). We like to thank the members of

THISEAS/ the Wellcome Trust Sanger Institute Genotyping Facility.

AMC-PAS/PROMIS

CARDIOGENICS Sample collection in the Cardiogenics Consortium (http://www.cardiogenics.eu/web/) was funded by the 6th Framework Program of the European Union (LSHM-CT-2006-037593) and supported through the Cambridge Bioresource which is funded by the NIHR Cambridge Biomedical research Centre. We thank all the participants and clinicians involved in the recruitment process at Cambridge and Leicester (UK), Luebeck and Regensburg (Germany), and Paris (France).

CHS This CHS research was supported by NHLBI contracts HHSN268201200036C, HHSN268200800007C, N01HC55222, N01HC85079, N01HC85080, N01HC85081, N01HC85082, N01HC85083, N01HC85086; NHLBI grants U01HL080295, R01HL087652, R01HL105756, R01HL103612, and R01HL120393, NIDDK grant R01DK089256 with additional contribution from the National Institute of Neurological Disorders and Stroke (NINDS). Additional support was provided through R01AG023629 from the National Institute on Aging (NIA). A full list of principal CHS investigators and institutions can be found at CHS-NHLBI.org. The provision of genotyping data was supported in part by the National Center for Advancing Translational Sciences, CTSI grant UL1TR000124, and the National Institute of Diabetes and Digestive and Kidney Disease Diabetes Research Center (DRC) grant DK063491 to the Southern California Diabetes Endocrinology Research Center. The content is solely the responsibility of the authors and does not necessarily represent the official views of the National Institutes of Health.

CoLaus The CoLaus study was supported by research grants from the Swiss National Science Foundation (grant no: 3200B0–105993, 3200B0-118308, 33CSCO-122661, 33CS30-139468 and 33CS30-148401), from GlaxoSmithKline, the Leenaards Foundation and the Faculty of Biology and Medicine of Lausanne, Switzerland. The authors also express their gratitude to the participants in the Lausanne CoLaus study and to the investigators who have contributed to the study in particular Vincent Mooser and Dawn Waterworth, and the research nurses for data collection. ZK received financial support from Swiss National Science Foundation (grant no: 31003A-143914) and the Leenaards Foundation.

Corogene The study was supported by grants from Aarne Koskelo Foundation, Helsinki University Central Hospital special government funds (EVO #TYH7215, #TKK2012005, #TYH2012209), and Finnish Foundation for Cardiovascular research. SM is supported by Academy of Finland (Grants 136895 and 263836).

deCODE We thank participants in deCODE cardiovascular- and obesity studies and collaborators for their cooperation. The research performed at deCODE Genetics was part funded through the European Community's Seventh Framework Programme (FP7/2007-2013), ENGAGE project, grant agreement HEALTH-F4-2007- 201413.

D.E.S.I.R This study was supported in part by grants from SFD ("Société Francophone du 358 Diabète"), CPER ("Contrat de Projets État-Région"), and ANR ("Agence Nationale de la 359 Recherche"). The D.E.S.I.R. study has been supported by INSERM contracts with CNAMTS, Lilly, Novartis Pharma and Sanofi-Aventis; by INSERM (Réseaux en Santé Publique, Interactions entre les déterminants de la santé), Cohortes Santé TGIR, the Association Diabète Risque Vasculaire, the Fédération Française de Cardiologie, La Fondation de France, ALFEDIAM, ONIVINS, Ardix Medical, Bayer Diagnostics, Becton Dickinson, Cardionics, Merck Santé, Novo Nordisk, Pierre Fabre, Roche, Topcon. We would also like to acknowledge the D.E.S.I.R study group. INSERM U780: B. Balkau, MA Charles, P. Ducimetière, E. Eschwège; INSERM U367: F. Alhenc-Gelas; CHU D’Angers: Y. Gallois, A. Girault; Bichat Hospital: F. Fumeron, M. Marre, R Roussel; CHU Rennes: F Bonnet; CNRS UMR8090, Lille: S. Cauchi, P. Froguel; Centres d’Examens de Santé: Alençon, Angers, Blois, Caen, Chartres, Chateauroux, Cholet, Le Mans, Orleans Tours; Institute de Recherche Médecine Générale: J. Cogneau; General practitioners of the region; Institute inter-Regional pour la Santé: C. Born, E. Caces, M. Cailleau, N Copin, O Lantieri, J.G. Moreau, F. Rakotozafy, J. Tichet, S. Vol.

DGI The Botnia (DGI) study have been supported by grants from Folkhälsan Research Foundation, Sigrid Juselius Foundation, Ministry of Education, Nordic Center of Excellence in Disease Genetics, Gyllenberg Foundation, Swedish Cultural Foundation in Finland, Finnish Diabetes Research Foundation, Foundation for Life and Health in Finland, Finnish Medical Society, Paavo Nurmi Foundation, Perklén Foundation, Ollqvist Foundation, Närpes Health Care Foundation, the Municipal Health Care Center and Hospital in Jakobstad, Health Care Centers in Vasa, Närpes and Korsholm. This work was also partially supported by NIH grant R01-DK075787 to JNH.

DIAGEN The DIAGEN study was supported by the Commission of the European Communities, Directorate C - Public Health and Risk Assessment, Health & Consumer Protection, Grant Agreement number - 2004310 and by the Dresden University of Technology Funding Grant, Med Drive. We are grateful to all of the patients who cooperated in this study and to their referring physicians and diabetologists in Saxony.

DNBC Funding support for the Danish National Birth Cohort (DNBC) was provided by the Danish National Research Foundation, the Danish Pharmacists’ Fund, the Egmont Foundation, the March of Dimes Birth Defects Foundation, the Augustinus Foundation and the Health Fund of the Danish Health Insurance Societies. The generation of GWAS genotype data for the DNBC samples was carried out within the GENEVA consortium with funding provided through the NIH Genes, Environment and Health Initiative (GEI) (U01HG004423). Assistance with phenotype harmonization and genotype cleaning, as well as with general study coordination, was provided by the GENEVA Coordinating Center (U01HG004446). Genotyping was performed at Johns Hopkins University Center for Inherited Disease Research, with support from the NIH GEI (U01HG004438).

DPS The DPS has been financially supported by grants from the Academy of Finland (117844 and 40758, 211497, and 118590; The EVO funding of the Kuopio University Hospital from Ministry of Health and Social Affairs (5254), Finnish Funding Agency for Technology and Innovation (40058/07), Nordic Centre of Excellence on Systems biology in controlled dietary interventions and cohort studies, SYSDIET (070014), The Finnish Diabetes Research Foundation, Yrjö Jahnsson Foundation (56358), Sigrid Juselius Foundation, Juho Vainio Foundation and TEKES grants 70103/06 and 40058/07.

DR’S EXTRA The DR's EXTRA Study was supported by the Ministry of Education and Culture of Finland (627;2004-2011), Academy of Finland (102318; 123885), Kuopio University Hospital, Finnish Diabetes Association, Finnish Foundations for Cardiovascular Research, Päivikki and Sakari Sohlberg Foundation, by European Commission FP6 Integrated Project (EXGENESIS); LSHM-CT-2004-005272, City of Kuopio and Social Insurance Institution of Finland (4/26/2010).

EGCUT This work was supported by the Targeted Financing from the Estonian Ministry of Science and Education [SF0180142s08]; the US National Institute of Health [R01DK075787]; the Development Fund of the University of Tartu (grant SP1GVARENG); the European Regional Development Fund to the Centre of Excellence in Genomics (EXCEGEN; grant 3.2.0304.11-0312); and through FP7 grant 313010.

ELY/FENLAND/ We are grateful to all the volunteers and to the staff of St. Mary's Street Surgery, Ely and the study team.

EPIC The Ely Study was funded by the MRC (MC_U106179471) and Diabetes UK. Genotyping in the Ely and

Fenland studies were supported in part by an MRC-GlaxoSmithKline pilot programme grant (G0701863).

The EPIC Norfolk diabetes case cohort study is nested within the EPIC Norfolk Study,which is supported by programme grants from the Medical Research Council, and Cancer Research UK and with additional support from the European Union, Stroke Association, British Heart Foundation, Research into Ageing, Department of Health, The Wellcome Trust and the Food Standards Agency. Genotyping was in part supported by the MRC-GSK pilot programme grant. We acknowledge the contribution of the staff and participants of the EPIC-Norfolk Study. The Fenland Study is funded by the Wellcome Trust and the Medical Research Council (MC_U106179471). We are grateful to all the volunteers for their time and help, and to the General Practitioners and practice staff for assistance with recruitment. We thank the Fenland Study Investigators, Fenland Study Co-ordination team and the Epidemiology Field, Data and Laboratory teams.

EMIL SWABIA Centre of Excellence Baden-Wuerttemberg “Metabolic Disorders” to BOB.

EPOGH- The European Union (HEALTH-2011.2.4.2-2-EU-MASCARA, HEALTH-F7-305507 HOMAGE, and the

FLEMENGHO European Research Council Advanced Researcher Grant-2011-294713-EPLORE) and the Fonds voor Wetenschappelijk Onderzoek Vlaanderen, Ministry of the Flemish Community, Brussels, Belgium (G.0881.13 and G.088013) supported FLEMENGHO.

FAMHS The Family Heart Study was supported by the by grants 1R01DK8925601 and 5R01DK075681 (IBB) Award (079895).

FIN-D2D The FIN-D2D study has been financially supported by the hospital districts of Pirkanmaa, South Ostrobothnia, and Central Finland, the Finnish National Public Health Institute (current National Institute for Health and Welfare), the Finnish Diabetes Association, the Ministry of Social Affairs and Health in Finland, the Academy of Finland (grant number 129293),Commission of the European Communities, Directorate C-Public Health (grant agreement no. 2004310) and Finland’s Slottery Machine Association.

FinGesture (Finnish Genetic Study of Arrhythmic Events) We thank the study participants. We also thank Juhani Junttila, Kari Kaikkonen, and Marja-Leena Kortelainen for study concept and design, and data acquisition and interpretation. The FinGesture cohort is supported by the Juselius Foundation (Helsinki, Finland) and the Council of Health of the Academy of Finland (Helsinki, Finland). Authors would like to thank Sylvain Foisy and Philippe Goyette for their contributions to the design, implementation and analysis of the GWA study of the FinGesture cohort. In addition, we would like to acknowledge the support of the Montreal Heart Institute Foundation.

FRAM This research was conducted in part using data and resources from the Framingham Heart Study of the National Heart Lung and Blood Institute of the National Institutes of Health and Boston University School of Medicine.   The analyses reflect intellectual input and resource development from the Framingham Heart Study investigators participating in the SNP Health Association Resource (SHARe) project. This work was partially supported by the National Heart, Lung and Blood Institute's Framingham Heart Study (Contract No. N01-HC-25195) and its contract with Affymetrix, Inc for genotyping services (Contract No. N02-HL-6-4278). This research was partially supported by grant NIDDK 1R01DK8925601. A portion of this research utilized the Linux Cluster for Genetic Analysis (LinGA-II) funded by the Robert Dawson Evans Endowment of the Department of Medicine at Boston University School of Medicine and Boston Medical Center.

FUSION Support for FUSION was provided by NIH grants R01-DK062370 (to M.B.), R01-DK072193 (to K.L.M.), and intramural project number 1Z01-HG000024 (to F.S.C.). Genome-wide genotyping was conducted by the Johns Hopkins University Genetic Resources Core Facility SNP Center at the Center for Inherited Disease Research (CIDR), with support from CIDR NIH contract no. N01-HG-65403.

GASP-1/GASP-2 The study was supported by grants from the Swedish Research Council, the Swedish Cancer Society, and the National Cancer Institute

GENDIAN The support of the physicians, the patients, and the staff of the Diabetes Zentrum Mergentheim (Head:

Prof. Dr. Thomas Haak), the diabetes outpatient clinic Dr Nusser - Dr Kreisel, the dialysis centers KfH Amberg, KfH Bayreuth, KfH Deggendorf, KfH Donauwörth, KfH Freising, KfH Freyung, KfH Fürth, KfH Hof, KfH Ingolstadt, KfH Kelheim, KfH München Elsenheimerstraße, KfH München-Schwabing, KfH Neumarkt, KfH Neusäß, KfH Oberschleißheim, KfH Passau, KfH Plauen, KfH Regensburg Günzstraße, KfH Regensburg Caritas-Krankenhaus, KfH Straubing, KfH Sulzbach-Rosenberg, KfH Weiden, Dialysezentrum Augsburg Dr. Kirschner, Dialysezentrum Bad Alexandersbad, KfH Bamberg, Dialysezentrum Emmering, Dialysezentrum Klinikum Landshut, Dialysezentrum Landshut, Dialysezentrum Pfarrkirchen, Dialysezentrum Schwandorf, Dr. Angela Götz, the medical doctoral student Johanna Christ and the Study Nurse Ingrid Lugauer. The expert technical assistance of Claudia Strohmeier is gratefully acknowledged. Phenotyping was funded by the Dr. Robert Pfleger-Stiftung (Dr Carsten A. Böger), the MSD Stipend Diabetes (Dr Carsten A. Böger) and the University Hospital of Regensburg (intramural grant ReForM A to Dr. A. Götz, ReForM C to Dr. Carsten Böger). Genome-wide genotyping was funded by the KfH Stiftung Präventivmedizin e.V. (Dr. Carsten A. Böger, Dr. Jens Brüning) and the University Hospital Regensburg (Dr Carsten A. Böger). Data analysis was funded by the Else Kröner-Fresenius Stiftung (Dr. Iris M. Heid and Dr. Carsten A. Böger: 2012_A147; Dr. Carsten A. Böger and Dr. Bernhard K. Krämer: P48/08//A11/08). **GENDIAN Study Group:** Mathias Gorski, Iris M. Heid, Bernhard K. Krämer, Myriam Rheinberger, Michael Broll, Alexander Lammert, Markus Schubert, Jens Brüning, Matthias Olden, Klaus Stark, Claudia Strohmeier, Emilia Ruff, Johanna Christ, Peter Nürnberg, Thomas Haak, Carsten A. Böger.

GEOS The GEOS Study was supported by the National Institutes of Health Genes, Environment and Health Initiative (GEI) Grant U01 HG004436, as part of the GENEVA consortium under GEI, with additional support provided by the Mid-Atlantic Nutrition and Obesity Research Center (P30 DK072488); and the Office of Research and Development, Medical Research Service, and the Baltimore Geriatrics Research, Education, and Clinical Center of the Department of Veterans Affairs. Genotyping services were provided by the Johns Hopkins University Center for Inherited Disease Research (CIDR), which is fully funded through a federal contract from the National Institutes of Health to the Johns Hopkins University (contract number HHSN268200782096C). Assistance with data cleaning was provided by the GENEVA Coordinating Center (U01 HG 004446; PI Bruce S Weir). Study recruitment and collection of datasets were supported by a Cooperative Agreement with the Division of Adult and Community Health, Centers for Disease Control and by grants from the National Institute of Neurological Disorders and Stroke (NINDS) and the NIH Office of Research on Women's Health (R01 NS45012, U01 NS069208-01).

GERMIFS I & II The German MI Family Studies (GerMIFS I-II were supported by the Deutsche Forschungsgemeinschaft and the German Federal Ministry of Education and Research (BMBF) in the context of the German National Genome Research Network (NGFN-2 and NGFN-plus), the EU funded integrated projects Cardiogenics (LSHM-CT-2006-037593) and ENGAGE, and the bi-national BMBF/ANR funded project CARDomics (01KU0908A).

GLACIER We thank the participants for their contributions to the GLACIER Study. We thank the Västerbottens Intervention Programme for data collection and the staff of the Umeå Medical Biobank for preparing the materials. The authors specifically thank K. Enqvist and T. Johansson (Västerbottens County Council, Umeå, Sweden) for DNA preparation and Sarah Edkins, Douglas Simpkin, and staff of the WTSI genotyping facility for genotyping. The GLACIER Study was funded by project grants from Novo Nordisk, the Swedish Heart-Lung Foundation, the Swedish Diabetes Association, the Påhlssons Foundation, the Swedish Research Council, and the Umeå Medical Research Foundation (all to P.W.F.). Inês Barroso acknowledges funding from the Wellcome Trust grant WT098051, United Kingdom NIHR Cambridge Biomedical Research Centre and the MRC Centre for Obesity and Related Metabolic Diseases. [representing authors: FR, SA, IB, PWF].

GOOD Financial support was received from the Swedish Research Council, the Swedish Foundation for Strategic Research, the ALF/LUA research grant in Gothenburg, the Lundberg Foundation, the Torsten and Ragnar Söderberg’s Foundation, the Novo Nordisk Foundation, and the European Commission grant HEALTH-F2-2008-201865-GEFOS.

HBCS We thank all study participants as well as everybody involved in the Helsinki Birth Cohort Study. Helsinki Birth Cohort Study has been supported by grants from the Academy of Finland (130326, 134791, 263924), the Finnish Diabetes Research Society, Folkhälsan Research Foundation, Novo Nordisk Foundation, Finska Läkaresällskapet, Signe and Ane Gyllenberg Foundation, Ahokas Foundation, Emil Aaltonen Foundation, Juho Vainio Foundation, Finnish Foundation for Pediatric Research, Sigrid Juselius Foundation, the Finnish Special Governmental Subsidy for Health Sciences, Samfundet Folkhälsan, Liv och Hälsa, and Wellcome Trust (grant number WT089062). We are grateful for the THL DNA laboratory for its skillful work to produce the DNA samples used in this study. We thank the Sanger Institute genotyping facilities for genotyping the samples.

HEALTH2006 The Health2006 study was financially supported by grants from the Velux Foundation; the Danish Medical Research Council, Danish Agency for Science, Technology and Innovation; the Aase and Ejner Danielsens Foundation; ALK-Abello´ A/S (Hørsholm, Denmark), Timber Merchant Vilhelm Bangs Foundation, MEKOS Laboratories Denmark) and Research Centre for Prevention and Health, the Capital Region of Denmark. This project was also funded by the Lundbeck Foundation and produced by The Lundbeck Foundation Centre for Applied Medical Genomics in Personalised Disease Prediction, Prevention and Care (LuCamp,www.lucamp.org). The Novo Nordisk Foundation Center for Basic Metabolic Research is an independent Research Center at the University of Copenhagen partially funded by an unrestricted donation from the Novo Nordisk Foundation ([www.metabol.ku.dk](http://www.metabol.ku.dk)).

HELIC This work was funded by the Wellcome Trust (098051) and the European Research Council (ERC-2011-StG 280559-SEPI). The MANOLIS cohort is named in honour of Manolis Giannakakis, 1978-2010. We thank the residents of Anogia and surrounding Mylopotamos villages, and of the Pomak villages, for taking part. The HELIC study has been supported by many individuals who have contributed to sample collection (including Antonis Athanasiadis, Olina Balafouti, Christina Batzaki, Georgios Daskalakis, Eleni Emmanouil, Chrisoula Giannakaki, Margarita Giannakopoulou, Anastasia Kaparou, Vasiliki Kariakli, Stella Koinaki, Dimitra Kokori, Maria Konidari, Hara Koundouraki, Dimitris Koutoukidis, Vasiliki Mamakou, Eirini Mamalaki, Eirini Mpamiaki, Maria Tsoukana, Dimitra Tzakou, Katerina Vosdogianni, Niovi Xenaki, Eleni Zengini), data entry (Thanos Antonos, Dimitra Papagrigoriou, Betty Spiliopoulou), sample logistics (Sarah Edkins, Emma Gray), genotyping (Robert Andrews, Hannah Blackburn, Doug Simpkin, Siobhan Whitehead), research administration (Anja Kolb-Kokocinski, Carol Smee, Danielle Walker) and informatics (Martin Pollard, Josh Randall).

HERITAGE The HERITAGE Family Study is supported by the National Heart, Lung, and Blood Institute Grant HL-Family Study 45670.

HUNT2 The Nord-Trøndelag Health Study (The HUNT Study) is collaboration between HUNT Research Centre (Faculty of Medicine, Norwegian University of Science and Technology NTNU), Nord-Trøndelag County Council, Central Norway Health Authority, and the Norwegian Institute of Public Health.

H2000 The Health 2000 Study was funded by the National Institute for Health and Welfare (THL), the Finnish Centre for Pensions (ETK), the Social Insurance Institution of Finland (KELA), the Local Government Pensions Institution (KEVA) and other organizations listed on the website of the survey (http://www.terveys2000.fi ). VS was supported by the Academy of Finland, grant number 139635 and the Finnish Foundation for Cardiovascular Disease. We are grateful for the THL DNA laboratory for its skillful work to produce the DNA samples used in this study. We thank the Sanger Institute genotyping facilities for genotyping the GenMets subcohort.

IMPROVE European Commission (Contract number: QLG1-CT-2002-00896), the Swedish Heart-Lung Foundation, the Swedish Research Council (8691), the Knut and Alice Wallenberg Foundation, the Foundation for Strategic Research, the Torsten and Ragnar Söderberg Foundation, the Strategic Cardiovascular Programme of Karolinska Institutet and the Stockholm County Council and the Stockholm County Council (560183).

InCHIANTI The InCHIANTI study baseline (1998-2000) was supported as a "targeted project" (ICS110.1/RF97.71) by the Italian Ministry of Health and in part by the U.S. National Institute on Aging (Contracts: 263 MD 9164 and 263 MD 821336).

INTER99 The Inter99 was initiated by Torben Jørgensen (PI), Knut Borch-Johnsen (co-PI), Hans Ibsen and Troels F. Thomsen. The steering committee comprises the former two and Charlotta Pisinger. The study was financially supported by research grants from the Danish Research Council, the Danish Centre for Health Technology Assessment, Novo Nordisk Inc., Research Foundation of Copenhagen County, Ministry of Internal Affairs and Health, the Danish Heart Foundation, the Danish Pharmaceutical Association, the Augustinus Foundation, the Ib Henriksen Foundation, the Becket Foundation, and the Danish Diabetes Association. This project was also funded by the Lundbeck Foundation and produced by The Lundbeck Foundation Centre for Applied Medical Genomics in Personalised Disease Prediction, Prevention and Care (LuCamp,www.lucamp.org). The Novo Nordisk Foundation Center for Basic Metabolic Research is an independent Research Center at the University of Copenhagen partially funded by an unrestricted donation from the Novo Nordisk Foundation ([www.metabol.ku.dk](http://www.metabol.ku.dk)).

Johnston The Johnston County Osteoarthritis Project is supported in part by cooperative agreements S043, 1734, County and S3486 from the Centers for Disease Control and Prevention/Association of Schools of Public Health; Osteoarthritis the NIAMS Multipurpose Arthritis and Musculoskeletal Disease Center grant 5-P60-AR30701; and the

NIAMS Multidisciplinary Clinical Research Center grant 5 P60 AR49465-03. Genotyping services were provided by Algynomics company.

KORA The KORA research platform (KORA, Cooperative Health Research in the Region of Augsburg) was initiated and financed by the Helmholtz Zentrum München - German Research Center for Environmental Health, which is funded by the German Federal Ministry of Education and Research and by the State of Bavaria. Furthermore, KORA research was supported  within the Munich Center of Health Sciences (MC Health), Ludwig-Maximilians-Universität, as part of LMUinnovativ.

Korkula/Split The CROATIA-Vis study was funded by grants from the Medical Research Council (UK) and Republic of

Croatia Ministry of Science, Education and Sports research grants to I.R. (108-1080315-0302). We would like to acknowledge the staff of several institutions in Croatia that supported the field work, including but not limited to The University of Split and Zagreb Medical Schools, the Institute for Anthropological Research in Zagreb and Croatian Institute for Public Health. The SNP genotyping for the CROATIA-Vis cohort was performed in the core genotyping laboratory of the Wellcome Trust Clinical Research Facility at the Western General Hospital, Edinburgh, Scotland. We would like to acknowledge the staff of several institutions in Croatia that supported the field work, including but not limited to The University of Split and Zagreb Medical Schools, the Institute for Anthropological Research in Zagreb and Croatian Institute for Public Health.

The CROATIA-Korcula study was funded by grants from the Medical Research Council (UK), European Commission Framework 6 project EUROSPAN (Contract No. LSHG-CT-2006-018947), European Commission Framework 7 project BBMRI-LPC (Contract No. 313010), Croatian Science Council (Grant no. 8875), and Republic of Croatia Ministry of Science, Education and Sports research grants to I.R. (108-1080315-0302). We would like to acknowledge the invaluable contributions of the recruitment team in Korcula, the administrative teams in Croatia and Edinburgh and the people of Korcula. The SNP genotyping for the CROATIA-Korcula cohort was performed in Helmholtz Zentrum München, Neuherberg, Germany.

The CROATIA-Split study was funded by grants from the Medical Research Council (UK), European Commission Framework 6 project EUROSPAN (Contract No. LSHG-CT-2006-018947) and Republic of Croatia Ministry of Science, Education and Sports research grants to I.R. (108-1080315-0302). We would like to acknowledge the staff of several institutions in Croatia that supported the field work, including but not limited to The University of Split and Zagreb Medical Schools and the Croatian Institute for Public Health. The SNP genotyping for the CROATIA-Split cohort was performed by AROS Applied Biotechnology, Aarhus, Denmark.

Lifelines The LifeLines Cohort Study, and generation and management of GWAS genotype data for the LifeLines Cohort Study is supported by the Netherlands Organization of Scientific Research NWO (grant 175.010.2007.006), the Economic Structure Enhancing Fund (FES) of the Dutch government, the Ministry of Economic Affairs, the Ministry of Education, Culture and Science, the Ministry for Health, Welfare and Sports, the Northern Netherlands Collaboration of Provinces (SNN), the Province of Groningen, University Medical Center Groningen, the University of Groningen, Dutch Kidney Foundation and Dutch Diabetes Research Foundation. We thank Behrooz Alizadeh, Annemieke Boesjes, Marcel Bruinenberg, Noortje Festen, Pim van der Harst, Ilja Nolte, Lude Franke, Mitra Valimohammadi for their help in creating the GWAS database, and Rob Bieringa, Joost Keers, René Oostergo, Rosalie Visser, Judith Vonk for their work related to data-collection and validation. The authors are grateful to the study participants, the staff from the LifeLines Cohort Study and Medical Biobank Northern Netherlands, and the participating general practitioners and pharmacists.

LOLIPOP The LOLIPOP study is supported by the National Institute for Health Research (NIHR) ComprehensiveBiomedical Research Centre Imperial College Healthcare NHS Trust, the British Heart Foundation (SP/04/002), the Medical Research Council (G0601966,G0700931), the Wellcome Trust (084723/Z/08/Z) the NIHR (RP-PG-0407-10371),European Union FP7 (EpiMigrant, 279143) and Action on Hearing Loss (G51). The work was carried out in part at the NIHR/Wellcome Trust Imperial Clinical Research Facility. The views expressed are those of the author(s) and not necessarily those of the Imperial College Healthcare NHS Trust, the NIHR or the Department of Health. We thank the participants and research staff who made the study possible.

LURIC We thank the LURIC study team who were either temporarily or permanently involved in patient recruitment as well as sample and data handling, in addition to the laboratory staff at the Ludwigshafen General Hospital and the Universities of Freiburg and Ulm. LURIC has received funding from the 6th Framework Program (integrated project Bloodomics, grant LSHM-CT-2004-503485) and from the 7th Framework Program (Atheroremo, grant agreement number 201668 and RiskyCAD, grant agreement number 305739) of the European Union as well as from the INTERREG IV Oberrhein Program (Project A28, Genetic mechanisms of cardiovascular diseases) with support from the European Regional Development Fund (ERDF) and the Wissenschaftsoffensive TMO.

MALE GOYA This study was conducted as part of the activities of the 'Gene-diet Interactions in Obesity' project (GENDINOB, www.gendinob.dk) and the MRC centre for Causal Analyses in Translational Epidemiology (MRC CAiTE). We thank the staff of the Copenhagen City Heart Study for their skillful examination of the study subjects in collection of baseline and follow-up data.

METSIM The METSIM study was funded by the Academy of Finland (grants no. 77299 and 124243).

MGS The Molecular Genetics of Schizophrenia project was carried out by 10 research sites and PIs: Pablo V. Gejman, Study Coordinator (Department of Psychiatry and Behavioral Sciences, NorthShore University HealthSystem, Evanston, IL, and Department of Psychiatry and Behavioral Sciences, University of Chicago, Chicago, IL), Douglas F. Levinson (Stanford University), Bryan J. Mowry (University of Queensland), Donald Black (University of Iowa), Robert Freedman (University of Colorado), C. Robert Cloninger (Washington University), Jeremy Silverman (Mt. Sinai Medical School), Nancy Buccola (Louisiana State University - New Orleans), William Byerley (University of California at San Francisco), and Farooq Amin (Emory University). This study was supported by NIH R01 grants (MH67257 to N.G.B., MH59588 to B.J.M., MH59571 to P.V.G., MH59565 to R.F., MH59587 to F.A., MH60870 to W.F.B., MH59566 to D.W.B., MH59586 to J.M.S., MH61675 to D.F.L., MH60879 to C.R.C., and MH81800 to P.V.G.), NIH U01 grants (MH79469 to P.V.G., and MH79470 to D.F.L.), NARSAD (National Alliance for Research on Schizophrenia and Depression) Young Investigator Awards (to J.D. and A.R.S.), the Genetic Association Information Network (GAIN), the Walter E. Nichols, M.D., and Eleanor Nichols endowments, at Stanford University, and by The Paul Michael Donovan Charitable Foundation. Genotyping was carried out by the Genotyping and Analysis at the Broad Institute of Harvard and MIT (S. Gabriel and D.B.M.), which is supported by grant U54 RR020278 from the National Center for Research Resources. Genotyping of half of the control sample presented here was carried out with support from GAIN. The GAIN quality control team (G.R. Abecasis and J. Paschall) made important contributions to the project. The statistical analysis team was coordinated by Douglas F. Levinson (Stanford University) and included Jianxin Shi (National Cancer Institute), Frank Dudbridge (London School of Hygiene and Tropical Medicine), Peter Holmans (Cardiff University) and Itsik Pe'er (Columbia University).

MICROS For the MICROS study, we thank the primary care practitioners Raffaela Stocker, Stefan Waldner, Toni

Pizzecco, Josef Plangger, Ugo Marcadent, and the personnel of the Hospital of Silandro (Department of Laboratory Medicine) for their participation and collaboration in the research project.The MICROS study was supported by the Ministry of Health and Department for Promotion of Educational Policies, Universities and Research of the Autonomous Province of Bolzano, South Tyrol, the South Tyrolean Sparkasse Foundation, and the European Union framework program 6 EUROSPAN project (contract no. LSHG-CT-2006-018947).

MIGen National Heart, Lung, and Blood Institute's STAMPEED genomics research program (R01 HL087676) and the National Center for Research Resources (U54 RR020278).

MRC NSHD This work was funded by the Medical Research Council (MC_UU_12019/1), the British Heart Foundation (RG/10/12/28456) and the Wellcome Trust (088869/B/09/Z). We are very grateful to the members of this birth cohort for their continuing interest and participation in the study. We would like to acknowledge the Swallow group, UCL, who performed the DNA extractions (Rousseau, et al 2006). DOI: 10.1111/j.1469-1809.2006.00250.x

NBS The Nijmegen Biomedical Study is a population-based survey conducted at the Department for Health Evidence, and the Department of Laboratory Medicine of the Radboud university medical center. Principal investigators of the Nijmegen Biomedical Study are Lambertus A. Kiemeney, André L.M. Verbeek, Dorine W. Swinkels and Barbara Franke. This work was sponsored by the Stichting Nationale Computerfaciliteiten (National Computing Facilities Foundation, NCF) for the use of supercomputer facilities, with financial support from the Nederlandse Organisatie voor Wetenschappelijk Onderzoek (Netherlands Organization for Scientific Research, NOW).

NELSON This study is supported by Zorg Onderzoek Nederland-Medische Wetenschappen, KWF Kankerbestrijding, Stichting Centraal Fonds Reserves van voormalig Vrijwillige Ziekenfondsverzekeringen, G. Ph. Verhagen Foundation, Rotterdam Oncologic Thoracic Study Group, Erasmus Trust Fund, Foundation against Cancer, Flemish League against Cancer, ITEA2 (project Care4Me), and Lokaal Gezondheids Overleg (LOGO) Leuven and Hageland. Roche Diagnostics provided an unrestricted research grant. Siemens Germany provided four digital workstations and accompanying software but had no other role in the study. COPACETIC (COPD Pathology: Addressing Critical gaps, Early Treatment & Diagnosis and Innovative Concepts) is funded by the European Union FP7 program (grant number: 201379).

NESDA Funding was obtained from the Netherlands Organization for Scientific Research (Geestkracht program grant 10-000-1002); the Center for Medical Systems Biology (CMSB, NWO Genomics), Biobanking and Biomolecular Resources Research Infrastructure (BBMRI-NL), VU University’s Institutes for Health and Care Research (EMGO+) and Neuroscience Campus Amsterdam, University Medical Center Groningen, Leiden University Medical Center, National Institutes of Health (NIH, R01D0042157-01A, MH081802, Grand Opportunity grants 1RC2 MH089951 and 1RC2 MH089995). Part of the genotyping and analyses were funded by the Genetic Association Information Network (GAIN) of the Foundation for the National Institutes of Health. Computing was supported by BiG Grid, the Dutch e-Science Grid, which is financially supported by NWO.

NFBC1966 NFBC1966 received financial support from the Academy of Finland (project grants 104781, 120315, 129269, 1114194, 24300796, Center of Excellence in Complex Disease Genetics and SALVE), University Hospital Oulu, Biocenter, University of Oulu, Finland (75617), NHLBI grant 5R01HL087679-02 through the STAMPEED program (1RL1MH083268-01), NIH/NIMH (5R01MH63706:02), ENGAGE project and grant agreement HEALTH-F4-2007-201413, EU FP7 EurHEALTHAgeing -277849 and the Medical Research Council, UK (G0500539, G0600705, G1002319, PrevMetSyn/SALVE). The DNA extractions, sample quality controls, biobank up-keeping and aliquotting was performed in the National Public Health Institute, Biomedicum Helsinki, Finland and supported financially by the Academy of Finland and Biocentrum Helsinki. We thank the late Professor Paula Rantakallio (launch of NFBC1966), and Ms Outi Tornwall and Ms Minttu Jussila (DNA biobanking). The authors would like to acknowledge the contribution of the late Academian of Science Leena Peltonen. Reedik Mägi was funded by EU FP7 Marie Curie IEF fellowship.

NHS NIH U01CA-098233, R01HL71981, DK091718, DK046200

NSPHS The Northern Sweden Population Health Study (NSPHS) was funded by the Swedish Medical Research Council (project number K2007-66X-20270-01-3), and the Foundation for Strategic Research (SSF). The NSPHS as part of EUROSPAN (European Special Populations Research Network) was also supported by European Commission FP6 STRP grant number 01947 (LSHG-CT-2006-01947). This work was also supported by the Swedish Society for Medical Research (ÅJ). The authors are grateful for the contribution of district nurse Svea Hennix for data collection and Inger Jonasson for logistics and coordination of the health survey. Finally, the authors thank all the community participants for their interest and willingness to contribute to the study.

NTR The NTR study would like to thank all of our study participants for their continuous voluntary contributions to our scientific efforts as well as the SURF SARA institute for their computational resources. Funding was obtained from the Netherlands Organization for Scientific Research (NWO: MagW/ZonMW): Genetic basis of anxiety and depression (904-61-090); Genetics of individual differences in smoking initiation and persistence (NWO 985-10-002); Resolving cause and effect in the association between exercise and well-being (904-61-193); Twin family database for behavior genomics studies (480-04-004); Twin research focusing on behavior (400-05-717); Genetic determinants of risk behavior in relation to alcohol use and alcohol use disorder (Addiction-31160008); Genotype/phenotype database for behavior genetic and genetic epidemiological studies (40-0056-98-9032); Spinozapremie (SPI 56-464-14192); CMSB: Center for Medical Systems Biology (NWO Genomics); NBIC/BioAssist/RK/2008.024); BBMRI –NL: Biobanking and Biomolecular Resources Research Infrastructure (184.021.007); the VU University: Institute for Health and Care Research (EMGO+ ) and Neuroscience Campus Amsterdam (NCA); the European Science Foundation (ESF): Genomewide analyses of European twin and population cohorts (EU/QLRT-2001-01254); European Community's Seventh Framework Program (FP7/2007-2013): ENGAGE (HEALTH-F4-2007-201413); the European Science Council (ERC) Genetics of Mental Illness (230374); Rutgers University Cell and DNA Repository cooperative agreement (NIMH U24 MH068457-06); Collaborative study of the genetics of DZ twinning (NIH R01D0042157-01A); the Genetic Association Information Network, a public–private partnership between the NIH and Pfizer Inc., Affymetrix Inc. and Abbott Laboratories.

ORCADES ORCADES was supported by the Chief Scientist Office of the Scottish Government, the Royal Society, the MRC Human Genetics Unit, Arthritis Research UK and the European Union framework program 6 EUROSPAN project (contract no. LSHG-CT-2006-018947). DNA extractions were performed at the Wellcome Trust Clinical Research Facility in Edinburgh. We would like to acknowledge the invaluable contributions of Lorraine Anderson and the research nurses in Orkney, the administrative team in Edinburgh and the people of Orkney.

PIVUS GWAS Genotyping was performed at the Sanger Institute. Metabochip genotyping was performed by the SNP&SEQ Technology Platform in Uppsala (www.genotyping.se). E.I. is supported by grants from the Swedish Research Council, the Swedish Heart-Lung Foundation, the Swedish Foundation for Strategic Research, and the Royal Swedish Academy of Science. Genotyping and data analyses were supported by grants from the Knut och Alice Wallenberg Foundation (Wallenberg Academy Fellow), European Research Council (ERC starting grant), Swedish Diabetes Foundation (grant no. 2013-024), Swedish Research Council (grant no. 2012-1397), and Swedish Heart-Lung Foundation (20120197).

PLCO/PLCO2 This study within the Prostate, Lung, Colorectal, and Ovarian (PLCO) Cancer Screening Trial was funded by the Intramural Research Program of the Division of Cancer Epidemiology and Genetics, National Cancer Institute, NIH. The authors thank Drs. Christine Berg and Philip Prorok, Division of Cancer Prevention, NCI, the screening center investigators and staff of the PLCO Cancer Screening Trial, Mr. Thomas Riley and staff at Information Management Services, Inc., and Ms. Barbara O’Brien and staff at Westat, Inc. for their contributions to the PLCO. Finally, we are grateful to the study participants for donating their time and making this study possible.

PREVEND The Dutch Kidney Foundation supported the infrastructure of the PREVEND program from 1997 to 2003 (Grant E.033) and from 2006 to 2010 (). The University Medical Center Groningen supported the infrastructure from 2003 to 2006.

PROCARDIS PROCARDIS was supported by the European Community Sixth Framework Program (LSHM-CT- 2007-037273), AstraZeneca, the Swedish Research Council, the Knut and Alice Wallenberg Foundation, the Swedish Heart-Lung Foundation, the Torsten and Ragnar Söderberg Foundation, the Strategic Cardiovascular Program of Karolinska Institutet and Stockholm County Council, the Foundation for Strategic Research and the Stockholm County Council (560283).

PROSPER/PHASE The PROSPER study was supported by an investigator initiated grant obtained from Bristol-Myers Squibb. Prof. Dr. J. W. Jukema is an Established Clinical Investigator of the Netherlands Heart Foundation (grant 2001 D 032). Support for genotyping was provided by the seventh framework program of the European commission (grant 223004) and by the Netherlands Genomics Initiative (Netherlands Consortium for Healthy Aging grant 050-060-810).

QFS The Quebec Family Study (QFS) was funded by multiple grants from the Medical Research Council of Canada and the Canadian Institutes for Health Research. This work was supoorted by a team grant from the Canadian Institutes for Health Reseaerch (FRCN-CCT-83028)

QIMR We acknowledge funding from the Australian National Health and Medical Research Council (NHMRC; 241944, 389875, 389891, 389892, 389938, 442915, 442981, 496739, 496688, 552485, 613672, 613601 and 1011506), the US National Institutes of Health (AA07535, AA10248, AA014041, AA13320, AA13321, AA13326 and DA12854) and the Australian Research Council (ARC; DP0770096 and DP1093502).

RISC The RISC Study is supported by European Union grant QLG1-CT-2001-01252 and AstraZeneca. Laura Pascoe is the recipient of a joint BBSRC and Unilever UK Ltd case PhD studentship.

ROS and MAP The ROS and MAP data used in this analysis was supported by National Institute on Aging grants P30AG10161, R01AG17917, R01AG15819, R01AG30146, the Illinois Department of Public Health, and the Translational Genomics Research Institute.

SCARFSHEEP European Commission (LSHM-CT- 2007- 037273), the Swedish Heart-Lung Foundation, the Swedish Research Council (8691), the Knut and Alice Wallenberg Foundation, the Foundation for Strategic Research, the Torsten and Ragnar Söderberg Foundation, the Strategic Cardiovascular Programme of Karolinska Institutet and the Stockholm County Council and the Stockholm County Council (560183).

RSI/RSII/ERF The Rotterdam Study is funded by Erasmus Medical Center and Erasmus University, Rotterdam, Netherlands Organization for the Health Research and Development (ZonMw), the Research Institute for Diseases in the Elderly (RIDE), the Ministry of Education, Culture and Science, the Ministry for Health, Welfare and Sports, the European Commission (DG XII), and the Municipality of Rotterdam. The authors are grateful to the study participants, the staff from the Rotterdam Study and the participating general practitioners and pharmacists. The generation and management of GWAS genotype data for the Rotterdam Study is supported by the Netherlands Organisation of Scientific Research NWO Investments (nr. 175.010.2005.011, 911-03-012). This study is funded by the Research Institute for Diseases in the Elderly (014-93-015; RIDE2), the Netherlands Genomics Initiative (NGI)/Netherlands Organisation for Scientific Research (NWO) project nr. 050-060-810. We thank Pascal Arp, Mila Jhamai, Marijn Verkerk, Lizbeth Herrera and Marjolein Peters for their help in creating the GWAS database, and Karol Estrada and Maksim V. Struchalin for their support in creation and analysis of imputed data. We would like to thank Karol Estrada, Dr. Fernando Rivadeneira, Dr. Tobias A. Knoch, Anis Abuseiris and Rob de Graaf (Erasmus MC Rotterdam, The Netherlands), for their help in creating GRIMP, and BigGRID and Services@MediGRID/D-Grid, (funded by the German Bundesministerium fuer Forschung und Technology; grants 01 AK 803 A-H, 01 IG 07015 G) for access to their grid computing resources. The Rotterdam Study is funded by Erasmus Medical Center and Erasmus University, Rotterdam, Netherlands Organization for the Health Research and Development (ZonMw), the Research Institute for Diseases in the Elderly (RIDE), the Ministry of Education, Culture and Science, the Ministry for Health, Welfare and Sports, the European Commission (DG XII), and the Municipality of Rotterdam. The authors are grateful to the study participants, the staff from the Rotterdam Study and the participating general practitioners and pharmacists. The generation and management of GWAS genotype data for the Rotterdam Study is supported by the Netherlands Organisation of Scientific Research NWO Investments (nr. 175.010.2005.011, 911-03-012). This study is funded by the Research Institute for Diseases in the Elderly (014-93-015; RIDE2), the Netherlands Genomics Initiative (NGI)/Netherlands Organisation for Scientific Research (NWO) project nr. 050-060-810. We thank Pascal Arp, Mila Jhamai, Marijn Verkerk, Lizbeth Herrera and Marjolein Peters for their help in creating the GWAS database, and Karol Estrada and Maksim V. Struchalin for their support in creation and analysis of imputed data. We would like to thank Karol Estrada, Dr. Fernando Rivadeneira, Dr. Tobias A. Knoch, Anis Abuseiris and Rob de Graaf (Erasmus MC Rotterdam, The Netherlands), for their help in creating GRIMP, and BigGRID and Services@MediGRID/D-Grid, (funded by the German Bundesministerium fuer Forschung und Technology; grants 01 AK 803 A-H, 01 IG 07015 G) for access to their grid computing resources. The ERF study as a part of EUROSPAN (European Special Populations Research Network) was supported by European Commission FP6 STRP grant number 018947 (LSHG-CT-2006-01947) and also received funding from the European Community's Seventh Framework Programme (FP7/2007-2013)/grant agreement HEALTH-F4-2007-201413 by the European Commission under the programme "Quality of Life and Management of the Living Resources" of 5th Framework Programme (no. QLG2-CT-2002-01254). The ERF study was further supported by ENGAGE consortium and CMSB. High-throughput analysis of the ERF data was supported by joint grant from Netherlands Organisation for Scientific Research and the Russian Foundation for Basic Research (NWO-RFBR 047.017.043). ERF was furterh supported by the ZonMw grant (project 91111025). We are grateful to all study participants and their relatives, general practitioners and neurologists for their contributions and to P. Veraart for her help in genealogy, J. Vergeer for the supervision of the laboratory work and P. Snijders for his help in data collection.

SardiNIA We thank all the volunteers who generously participated in this study, Monsignore Piseddu, Bishop of Ogliastra and the mayors and citizens of the Sardinian towns (Lanusei, Ilbono, Arzana, and Elini). This work was supported by the Intramural Research Program of the National Institute on Aging (NIA), National Institutes of Health (NIH). The SardiNIA (“Progenia”) team was supported by Contract NO1-AG-1–2109 from the NIA; the efforts of GRA were supported in part by contract 263-MA-410953 from the NIA to the University of Michigan and by research grant HG002651 and HL084729 from the NIH (to GRA).

SEARCH Cancer Research UK (C490/A10124, C490/A10119). People: Douglas Easton, Paul Pharoah, the SEARCH team.

SHIP/SHIP-TREND SHIP is part of the Community Medicine Research net of the University of Greifswald, Germany, which is

funded by the Federal Ministry of Education and Research (grants no. 01ZZ9603, 01ZZ0103, and 01ZZ0403), the Ministry of Cultural Affairs as well as the Social Ministry of the Federal State of Mecklenburg-West Pomerania, and the network ‘Greifswald Approach to Individualized Medicine (GANI_MED)’ funded by the Federal Ministry of Education and Research (grant 03IS2061A). Genome-wide data have been supported by the Federal Ministry of Education and Research (grant no. 03ZIK012) and a joint grant from Siemens Healthcare, Erlangen, Germany and the Federal State of Mecklenburg- West Pomerania. The University of Greifswald is a member of the ‘Center of Knowledge Interchange’ program of the Siemens AG and the Caché Campus program of the InterSystems GmbH.

STR This work was supported by grants from the Swedish Ministry for Higher Education, the US National Institutes of Health (AG028555, AG08724, AG04563, AG10175, AG08861), the Swedish Research Council. Genotyping was performed by the SNP&SEQ Technology Platform in Uppsala (www.genotyping.se). We thank Tomas Axelsson, Ann-Christine Wiman and Caisa Pöntinen for their excellent assistance with genotyping. The SNP Technology Platform is supported by Uppsala University, Uppsala University Hospital and the Swedish Research Council for Infrastructures.

SORBS This work was supported by grants from the German Research Council (SFB- 1052 "Obesity mechanisms" to Michael Stumvoll, Anke Tönjes and Peter Kovacs), from the German Diabetes Association (to Anke Tönjes and Peter Kovacs) and from the DHFD (Diabetes Hilfs- und Forschungsfonds Deutschland to Michael Stumvoll and Peter Kovacs). Peter Kovacs is funded by the Boehringer Ingelheim Foundation. We thank all those who participated in the study. Sincere thanks are given to Knut Krohn (Microarray Core Facility of the Interdisciplinary Centre for Clinical Research, University of Leipzig) for the genotyping support. Reedik Mägi is funded by European Commission under the Marie Curie Intra-European Fellowship and by Estonian Government (grant #SF0180142s08).

THISEAS Recruitment for THISEAS was partially funded by a research grant (PENED 2003) from the Greek General Secretary of Research and Technology; we thank all the dieticians and clinicians for their contribution to the project.

TRAILS This research is part of the TRacking Adolescents' Individual Lives Survey (TRAILS). Participating centers of TRAILS include various departments of the University Medical Center and University of Groningen, the Erasmus University Medical Center Rotterdam, the University of Utrecht, the Radboud Medical Center Nijmegen, and the Parnassia Bavo group, all in the Netherlands. TRAILS has been financially supported by various grants from the Netherlands Organization for Scientific Research NWO (Medical Research Council program grant GB-MW 940-38-011; ZonMW Brainpower grant 100-001-004; ZonMw Risk Behavior and Dependence grants 60-60600-97-118; ZonMw Culture and Health grant 261-98-710; Social Sciences Council medium-sized investment grants GB-MaGW 480-01-006 and GB-MaGW 480-07-001; Social Sciences Council project grants GB-MaGW 452-04-314 and GB-MaGW 452-06-004; NWO large-sized investment grant 175.010.2003.005; NWO Longitudinal Survey and Panel Funding 481-08-013), the Dutch Ministry of Justice (WODC), the European Science Foundation (EuroSTRESS project FP-006), Biobanking and Biomolecular Resources Research Infrastructure BBMRI-NL (CP 32), and the participating universities. We are grateful to all adolescents, their parents and teachers who participated in this research and to everyone who worked on this project and made it possible. Statistical analyses were carried out on the Genetic Cluster Computer (http://www.geneticcluster.org), which is financially supported by the Netherlands Scientific Organization (NWO 480-05-003) along with a supplement from the Dutch Brain Foundation.

Tromsø University of Tromsø, Norwegian Research Council (project number 185764)

TWINGENE This work was supported by grants from the Ministry for Higher Education, the Swedish Research Council (M-2005-1112 and 2009-2298), GenomEUtwin (EU/QLRT-2001-01254; QLG2-CT-2002-01254), NIH grant DK U01-066134, The Swedish Foundation for Strategic Research (SSF; ICA08-0047), the Swedish Heart-Lung Foundation, the Royal Swedish Academy of Science, and ENGAGE (within the European Union Seventh Framework Programme, HEALTH-F4-2007-201413).

TwinsUK The study was funded by the Wellcome Trust; European Community’s Seventh Framework Programme (FP7/2007-2013). The study also receives support from *the National Institute for Health Research (NIHR) Clinical Research Facility at Guy’s & St Thomas’ NHS Foundation Trust and NIHR Biomedical Research Centre based at Guy's and St Thomas' NHS Foundation Trust and King's College London.* Tim Spector is an NIHR senior Investigator and is holder of an ERC Advanced Principal Investigator award. SNP Genotyping was performed by The Wellcome Trust Sanger Institute and National Eye Institute via NIH/CIDR.

ULSAM Genotyping was performed by the SNP&SEQ Technology Platform in Uppsala (www.genotyping.se). We thank Tomas Axelsson, Ann-Christine Wiman and Caisa Pöntinen for their excellent assistance with genotyping. The SNP Technology Platform is supported by Uppsala University, Uppsala University Hospital and the Swedish Research Council for Infrastructures. E.I. is supported by grants from the Swedish Research Council, the Swedish Heart-Lung Foundation, the Swedish Foundation for Strategic Research, and the Royal Swedish Academy of Science. Genotyping and data analyses were supported by grants from the Knut och Alice Wallenberg Foundation (Wallenberg Academy Fellow), European Research Council (ERC starting grant), Swedish Diabetes Foundation (grant no. 2013-024), Swedish Research Council (grant no. 2012-1397), and Swedish Heart-Lung Foundation (20120197).

WGHS The WGHS is supported by HL043851 and HL080467 from the National Heart, Lung, and Blood Institute and CA047988 from the National Cancer Institute with collaborative scientific support and funding for genotyping provided by Amgen.

WHITEHALL II The WHII study has been supported by grants from the Medical Research Council; British Heart Foundation; Health and Safety Executive; Department of Health; National Heart Lung and Blood Institute (NHLBI: HL36310) and National Institute on Aging (AG13196), US, NIH; Agency for Health Care Policy Research (HS06516); and the John D and Catherine T MacArthur Foundation Research Networks on Successful Midlife Development and Socio-economic Status and Health.

YFS The Young Finns Study has been financially supported by the Academy of Finland: grants 134309 (Eye), 126925, 121584, 124282, 129378 (Salve), 117787 (Gendi), and 41071 (Skidi), the Social Insurance Institution of Finland, Kuopio, Tampere and Turku University Hospital Medical Funds (grant 9M048 and 9N035 for TeLeht), Juho Vainio Foundation, Paavo Nurmi Foundation, Finnish Foundation of Cardiovascular Research and Finnish Cultural Foundation, Tampere Tuberculosis Foundation and

Emil Aaltonen Foundation (T.L). The THL DNA laboratory for its skillful work to produce the DNA

samples used in this study and the expert technical assistance in the statistical analyses by Ville Aalto

and Irina Lisinen are gratefully acknowledged.
